# Social determination in the health of children born preterm: a scoping review ^*^

**DOI:** 10.1590/1518-8345.7720.4605

**Published:** 2025-07-11

**Authors:** Mariana Fuentes Mendoza Rodrigues Soares, Amanda de Azevedo Gomes, Juliana Barony da Silva, Luciano Marques dos Santos, Patrícia Pinto Braga, Elysângela Dittz Duarte

**Affiliations:** 1 Universidade Federal de Minas Gerais, Escola de Enfermagem, Belo Horizonte, MG, Brazil.; 2 Universidade Estadual de Feira de Santana, Departamento de Saúde, Feira de Santana, BA, Brazil.; 3 Universidade Federal de São João del Rei, Curso de Enfermagem, Divinópolis, MG, Brazil.

**Keywords:** Social Determinants of Health, Structural Determinants of Health, Premature Infant, Child Health, Growth and Development, Child Care

## Abstract

to map scientific knowledge on the dimensions of the Social Determinants of Health in the health of children born preterm in the first two years of life.

this is a scoping review guided by the Joanna Briggs Institute and the Preferred Reporting Items for Systematic Reviews and Meta-Analyses extension for Scoping Reviews. From the studies found in five databases and the reference list of those selected, data was extracted using a coding tool based on the Social Determinants of Health theoretical model with the support of the MaxQDA software.

31 publications were included. The most investigated dimensions of the social determinants of children’s health were related to the characteristics of the individual, with emphasis on health conditions and age, sex, and hereditary factors, highlighting the search for factors that have a direct impact on children’s health.

the dimensions of child health most explored were those related to child development. Behavior and living conditions were little investigated. Future studies with a broader approach to the Social Determinants of Health in an integrated way could contribute to the development of care centered on premature infants and early interventions concerning child health outcomes.

## Highlights

**(1)** Studies highlight the influence of individual characteristics on child health. **(2)** Social determinants interfere with development and the demand for health services. **(3)** There are gaps in the investigation of SDH and their effects on child health. **(4)** The need to address the Social Determinants of Health in an integrated manner.

## Introduction

Although the risk factors for prematurity are widely acknowledged^(1)^, the disparities that contribute to preterm birth and its infant health outcomes are still poorly understood. A growing body of evidence highlights the importance of also addressing social and environmental factors to prevent preterm birth and improve the quality of life of families who have children born preterm^(2-3)^.

In addition to the disparities inherent in preterm birth, the circumstances in which individuals find themselves, such as their living and working conditions, have a direct impact on their health situation^(4)^ and are defined as Social Determinants of Health (SDH)^(5)^. These are defined as Social Determinants of Health^(5)^. These factors have been schematized in layers, highlighting the levels of coverage of the SDH on the individual and the other layers, which can be closer^(6)^, being characterized by individual domains such as individual characteristics, sex, and hereditary factors, or more external, and macro-determinants, exemplified by economic, cultural and environmental conditions^(4)^. These components, which include individual characteristics, structural factors, and public policies, modify health, exposure to disease, and access to the necessary care^(7)^.

It is known that children’s health conditions can be affected by perinatal circumstances, including care, nutritional, socioeconomic, and cultural conditions, which influence developmental outcomes in the short and long term, especially in early childhood^(8-9)^. In this context, children’s growth, behavior, and development are not only determined by biological mechanisms but also by the continuous interaction between the individual characteristics of the children, their parents, and the environment in which they live^(8-9)^. This interaction is particularly relevant in prematurity, given that the prevalence of behavioral and emotional problems in children born preterm is associated not only with the intrinsic characteristics of prematurity but also with various family characteristics and behaviors^(10-11)^.

The child’s first two years, the time frame of childhood defined for this review, are considered essential for growth and the development of skills and abilities since it is during this period that the most significant brain growth occurs^(12)^. Therefore, it is essential to provide a welcoming environment, learning opportunities, stimuli, and interactions that contribute to child development and can have repercussions throughout life^(12)^. Considering that prematurity and hospitalization in the Neonatal Intensive Care Unit (NICU) are risk factors for growth and development, families must be monitored by health services for health surveillance and early interventions, to minimize the possible damage caused to children^(12-13)^.

Recognizing the magnitude of preterm birth in the context of global public health, there has been research into its implications for the lives of children and their families^(13-14)^, especially from the individual, biological perspectives, focusing on factors specific to SDH, and infant morbidity and mortality^(15)^. These findings offer a limited understanding of the aspects that can be modified and still make a limited contribution to advances in the care of children born prematurely. Given the complexity of the social determination of health, it is important to clarify the interference of factors such as education, neighborhood, environment, social support network, and access to health services in the lives of children born preterm in the first two years of life. These factors have the potential to influence health outcomes such as mortality, morbidity, life expectancy, health status, health expenditure, and functional limitations^(16)^.

Given the above, it is understood that the domains that constitute SDH offer a basis for an expanded investigation and contribute to a more comprehensive investigation of the effects of the way of life and illness of children born preterm in the first two years of life, taking into account the circumstances to which they are exposed^(17)^.

The scoping review seeks to map the evidence of the SDH’s influence on the health of children born preterm in the first two years. Although the biological factors of prematurity are well documented, social and structural inequalities are still little explored in an integrated way. This approach makes it possible to capture the complexity of SDH, identify gaps in knowledge, and guide future research, broadening understanding of its impact on the development and quality of life of these children and their families. Therefore, this study aimed to map scientific knowledge on the dimensions of SDH in the health of children born preterm in the first two years of life.

## Method

### Study type

This is a scoping review guided by the Joanna Briggs Institute (JBI), Manual for Evidence Synthesis^(18)^, and the international guide Preferred Reporting Items for Systematic Reviews and Meta-Analyses extension for Scoping Reviews (PRISMA-ScR)^(19)^, conducted in five stages: elaboration of the research question, identification of relevant studies, selection of studies, data analysis, and synthesis. This methodology aims to map the available evidence and identify knowledge gaps^(20)^. This review presents the results of one of the research questions drawn up in the review protocol, which has been registered on the Open Science Framework (OSF) platform and can be accessed via the link https://osf.io/p724a/ with DOI 10.17605/OSF.IO/P724A.

The guiding question for this review was elaborated according to the mnemonic Population, Concept, and Context (PCC), with the population being: children born preterm in the first two years of life; the concept: dimensions of SDH; and the context: health of children born preterm in the first two years of life.

The guiding question was: What dimensions of SDH have been addressed in the literature on the health of children born preterm in the first two years of life? Secondary questions were developed to support the achievement of the objectives: What are the studies’ methodological approaches? What are the characteristics of the population studied? What factors have been investigated as SDH? What dimensions of SDH have been covered in the studies? What child health outcomes have been related to SDH?

### Selection criteria

Inclusion criteria were original studies in English, Portuguese, or Spanish, published in the last 13 years (2011 to 2024), which considered children born preterm in the first two years of life in the home context and which addressed SDH and its relationship with health in this period. The time frame was defined to map knowledge in a more recent period, which could reflect the living conditions of the population studied. Opinion articles, editorials, letters, literature reviews, gray literature, and studies that did not answer the research question were excluded.

### Study variables

The variables collected to characterize each study include the title, type of study, country, year of publication, objective, dimensions assessed, and sample. Child variables were also identified, which comprise the SDH codes and subcodes such as economic, social, cultural, and environmental conditions (socioeconomic, environmental, cultural, political, and public policy); living and working conditions (access to food; access to essential services; education; housing conditions; and working conditions); social and community support networks (support networks); individual behavioral and lifestyle factors (family care; kangaroo care; attachment; breastfeeding; and unhealthy habits); individual characteristics (health conditions; age, sex, and hereditary factors; psychological factors; and ethnic and racial factors); and child health outcomes (development; behavior; growth; illnesses; child mortality; and demands for health services).

### Information sources and search strategies

The search strategy was built by a librarian and used in five databases, MEDLINE/PubMed, Virtual Health Library (VHL), Excerpta Medica Database (EMBASE), Scopus, and Web of Science. This stage was carried out in March 2024 using the search strategies described in Figure 1.

**Figure 1 -:** Description of the search strategies used in each database. Belo Horizonte, MG, Brazil, 2024

**Database**	**Search strategy**
VHL*	(“Recém-Nascido Prematuro” OR “Infant, Premature” OR “Recien Nacido Prematuro” OR prématuré OR “Bebê Prematuro” OR “Bebês Prematuros” OR prematuridade OR prematuro OR prematuros OR “Recém-Nascidos Prematuros” OR “Nascimento Prematuro” OR “Premature Birth” OR “Nacimiento Prematuro” OR “Naissance prématurée” OR “Premature Infant” OR “Premature Infants”) AND (“Determinantes Sociais da Saúde” OR “Social Determinants of Health” OR “Determinantes Sociales de la Salud” OR “Déterminants sociaux de la santé” OR “Determinante de Saúde” OR “Determinantes Estruturais da Saúde” OR “Determinantes Estruturais de Saúde” OR “Determinantes Sociais de Saúde” OR “Fatores Socioeconômicos” OR “Socioeconomic Factors” OR “Factores Socioeconómicos” OR “Facteurs socioéconomiques” OR “Aspectos Socioeconômicos” OR “Fatores Sociais” OR “Social Factors” OR “Factores Sociales” OR “Fatores Culturais” OR “Cultural Factors” OR “Factores Culturales” OR “Facteurs Culturels” OR “Fatores Econômicos” OR “Economic Factors” OR “Factores Económicos” OR “Facteurs économiques” OR “Fatores Raciais” OR “Race Factors” OR “Factores Raciales” OR “Facteurs raciaux” OR or “Condições Sociais” OR “Social Conditions” OR “Condiciones Sociales” OR “Conditions sociales” OR “Condição Social” OR “Condições de Vida” OR “Política de Saúde” OR “Health Policy” OR “Política de Salud” OR “Politique de santé” OR “Health Social Determinant” OR “Health Social Determinants” OR “Health Structural Determinant” OR “Health Structural Determinants” OR “Structural Determinants of Health” OR “Living Condition” OR “Living Conditions” OR “Social Condition”) AND (“Cuidado do Lactente” OR “Infant Care” OR “Cuidado del Lactante” OR “Soins du nourrisson” OR família OR family OR familia OR famille) AND ( db:(“LILACS” OR “IBECS” OR “BDENF” OR “INDEXPSI” OR “BINACIS” OR “BBO” OR “CUMED” OR “LIPECS” OR “MedCarib” OR “SES-SP” OR “colecionaSUS” OR “PAHOIRIS” OR “BIGG” OR “MULTIMEDIA” OR “CidSaude” OR “PAHO” OR “PIE” OR “PREPRINT-MEDRXIV” OR “PREPRINT-SCIELO” OR “VETINDEX”))
**MEDLINE/ PubMed**	(“Infant, Premature” OR “Premature Birth” OR “Premature Infant” OR “Premature Infants”) AND (“Social Determinants of Health” OR “Socioeconomic Factors” OR “Social Factors” OR “Cultural Factors” OR “Economic Factors” OR “Race Factors” OR “Social Conditions” OR “Health Policy” OR “Health Social Determinant” OR “Health Social Determinants” OR “Health Structural Determinant” OR “Health Structural Determinants” OR “Structural Determinants of Health” OR “Living Condition” OR “Living Conditions” OR “Social Condition”) AND (“Infant Care” OR Family)
**Scopus** (Via *Portal CAPES* ^†^ )	(“Infant, Premature” OR “Premature Birth” OR “Premature Infant” OR “Premature Infants”) AND (“Social Determinants of Health” OR “Socioeconomic Factors” OR “Social Factors” OR “Cultural Factors” OR “Economic Factors” OR “Race Factors” OR “Social Conditions” OR “Health Policy” OR “Health Social Determinant” OR “Health Social Determinants” OR “Health Structural Determinant” OR “Health Structural Determinants” OR “Structural Determinants of Health” OR “Living Condition” OR “Living Conditions” OR “Social Condition”) AND (“Infant Care” OR Family)
**Web of Science** (Via *Portal CAPES* ^†^ )	(“Infant, Premature” OR “Premature Birth” OR “Premature Infant” OR “Premature Infants”) AND (“Social Determinants of Health” OR “Socioeconomic Factors” OR “Social Factors” OR “Cultural Factors” OR “Economic Factors” OR “Race Factors” OR “Social Conditions” OR “Health Policy” OR “Health Social Determinant” OR “Health Social Determinants” OR “Health Structural Determinant” OR “Health Structural Determinants” OR “Structural Determinants of Health” OR “Living Condition” OR “Living Conditions” OR “Social Condition”) AND (“Infant Care” OR Family)
**EMBASE** ^‡^ (Via *Portal CAPES* ^†^ )	(prematurity) and (‘social determinants of health’ or socioeconomics or ‘social aspect’ or ‘cultural factor’ or ‘economic aspect’ or ‘social status’ or ‘health care policy’) and (‘infant care’ or family)

*VHL = Virtual Health Library; ^†^CAPES = Coordination for the Improvement of Higher Education Personnel; ^‡^EMBASE = Excerpta Medica Database

### Selection of evidence sources and data extraction process

The process of selecting the articles included was conducted by two independent researchers and supported by Rayyan software. Initially, duplicate studies were excluded, and the titles and abstracts of the remaining articles were read according to the eligibility criteria. The remaining studies were read in full, following the same criteria. Any discrepancies identified during the selection process were analyzed by a third researcher.

### Data analysis

The data from the articles was extracted using a coding tool based on the SDH model^(6)^, aligned with the article’s objectives and the PCC. This instrument was structured in the MaxQDA software and guided the search for information to collect the study’s characterization variables, as well as the SDH codes and subcodes.

The economic, social, cultural, and environmental conditions code refers to the economic, cultural, and environmental conditions of society^(4)^, and mainly explores the care scenario in which the child is living. It includes the subcodes: socio-economic factors (availability or not of material resources and opportunities); cultural factors (values, ideals, interests, and behaviors of a given social group; including religion); political factors and public policies (public policies aimed at reducing social inequalities, promoting changes in behavior, easier access to healthy food, programs that improve access to health and education for the population. Also considered are social assistance programs, and institutionalization of care^(4)^).

Living and working conditions mainly elucidate the care context to which the child is exposed and refer to the availability of food and access to environments and essential services, such as health and education^(4)^. This code includes the subcodes: access to food (availability of and access to adequate food); access to essential services (education and health services); education (level of schooling and education of parents and family members of preterm children); housing conditions (housing conditions, including location, structure and basic sanitation), and working conditions (occupation of the family member; whether they have a paid job or are unemployed)^(4)^.

Community social and support networks correspond to community and support networks, family, and religious ties^(7)^. The individual’s behavioral and lifestyle factors include the subcodes: family care (child care offered by the family at home; kangaroo care, skin-to-skin contact, which starts early and grows from touch to the kangaroo position^(21)^; attachment, bonding, lasting ties established between babies and mothers or caregivers^(21)^); breastfeeding (practice of breastfeeding), and unhealthy habits (lifestyle habits of caregivers that can harm children’s health, such as alcohol and other drug consumption, poor diet and sedentary lifestyle).

Individuals’ characteristics include health conditions (circumstances in the newborn’s health that require responses through health care. For example, medical diagnoses assigned); age, sex, and hereditary factors (factors associated with the age of individuals, sex, and heredity); psychological factors (covers aspects of the mental health of individuals present in the family context that can influence child health); ethnic and racial factors (factors related to ethnic and racial groups). Finally, children’s health outcomes were identified when related to the child’s health conditions, growth, and neuropsychomotor development resulting from the influence of SDH.

The final sample of articles was imported into MaxQDA software version 2022, which supported the coding of the information. This process was carried out independently by two authors of this study, and the coded excerpts were validated by a third researcher.

## Results

**Figure 2 -:**
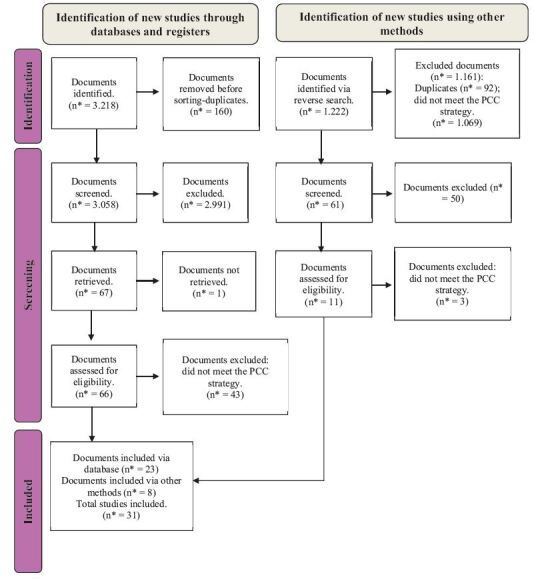
Flow diagram of the article selection process, according to PRISMA-SCR^†^. Belo Horizonte, MG, Brazil, 2024

Figure 2 shows the article selection process. Initially, 3,218 studies were imported, 160 of which were excluded due to duplication. After reading the titles and abstracts, 2,991 were excluded, leaving 67 studies to be read in full and the reverse search carried out, with a further 11 studies having to be read. One of the studies was not found in full, so 77 studies were read in full, and of these, 31 made up the final sample.

*n = Number; ^†^PRISMA-SCR = Preferred Reporting Items for Systematic reviews and Meta-Analyses extension for Scoping Reviews

The 31 articles included in this study are shown in Figure 3, including the title, type of study/country/year of publication, objective, dimensions assessed, and sample.

Of the articles analyzed, the majority (80.65%) took a quantitative approach. As for the continents of origin of the publication, 19 studies were carried out in North America (A3, A4, A5, A6, A8, A9, A10, A11, A12, A13, A16, A17, A19, A20, A22, A23, A25, A27, A31), six in Europe (A1, A2, A7, A18, A28, A29), four in South America (A14, A15, A21, A24) and two in Asia (A26, A30). The year with the highest number of publications was 2021, with nine articles, and there were no publications in 2014. In the other years, the average number of publications was 2.38.

**Figure 3 -:** Characterization of the included studies. Belo Horizonte, MG, Brazil, 2024

**Article code**	**Title**	**Study type, country, (year of publication)**	**Objective**	**Dimensions assessed**	**Sample**
A01 ^( 22 )^	Psychological distress and early lactation performance in mothers of late preterm infants	Control case, Italy, (2011).	To examine the relationship between the psychological distress of mothers who had a late preterm birth and their performance at the beginning of lactation.	Behavioral and lifestyle factors of the individual; characteristics of the individual.	Case group: 42 mothers of PTNB* born with a GA ^†^ between 34 and 36 weeks. Control group: 42 mothers of term NB ^‡^ .
A02 ^( 23 )^	Parenting stress in mothers after very preterm birth and the effect of the Infant Behavioural Assessment and Intervention Program	Control case, Netherlands, (2011).	To examine maternal parental stress as a secondary outcome of the Infant Behavioral Assessment and Intervention Program (IBAIP) ^§^ .	Economic, social, cultural, and environmental conditions; living and working conditions; social and support networks in the community; behavioral and lifestyle factors of the individual; characteristics of the individual.	Intervention group: 86 PTNB* (born with <32 weeks of GA ^†^ and/or birth weight <1500 grams) and 72 parents. Control group: 90 PTNB* (born with < 32 weeks of GA ^†^ and/or birth weight < 1500 grams) and 79 parents.
A03 ^( 24 )^	Evaluation of the ability of neurobiological, neurodevelopmental and socio-economic variables to predict cognitive outcome in premature infants	Longitudinal, United States, (2011).	To re-evaluate the effectiveness of the Neurobiologic Risk Score (NBRS) ^||^ and Neurodevelopmental Risk Exam (NRE) ^¶^ and assess the relative predictive role of socioeconomic risk factors, alone and in combination with known biological and developmental risk factors.	Living and working conditions; social and support networks in the community; characteristics of the individual.	129 PTNB* (born at < 32 weeks of GA ^†^ ).
A04 ^( 25 )^	Effect of ethnicity and race on cognitive and language testing at 18 – 22 months in extremely preterm infants	Retrospective cohort, United States, (2012).	To evaluate the relationship between race/ethnicity and cognitive and language scores on the Bayley Scales of Infant and Toddler Development, Third Edition (BSID-III)** in extreme preterm babies (<28 weeks of GA ^†^ ).	Living and working conditions; characteristics of the individual.	865 extremely preterm NB ^‡^ (born at < 28 weeks of GA ^†^ ).
A05 ^( 26 )^	Parenting stress, social support, and mother-child interactions in families of multiple and singleton preterm toddlers	Longitudinal, United States, (2012).	To investigate family support as a potential moderator of maternal parental stress in families with multiple and single premature children.	Economic, social, cultural, and environmental conditions; living and working conditions; social and support networks in the community; behavioral and lifestyle factors of the individual; characteristics of the individual.	153 families and PTNB* (born at < 37 weeks of GA ^†^ ).
A06 ^( 27 )^	Impact of maternal education on cognitive and language scores at 18 to 24 months among extremely preterm neonates	Cohort, Canada, (2013).	To explore the association between maternal schooling levels and the composite cognitive and language scores of the Bayley Scale III, at 18 to 24 months of corrected age in extremely preterm babies born at < 29 weeks of GA ^†^ .	Living and working conditions; social and support networks in the community; characteristics of the individual.	457 families and extremely premature (born at < 29 weeks of GA ^†^ ).
A07 ^( 28 )^	One-year neurodevelopmental outcome of very and late preterm infants: risk factors and correlation with maternal stress	Longitudinal, Italy, (2015).	To describe the developmental profile of late preterm infants, to compare cognitive, linguistic, and motor development in late preterm and very preterm infants at 12 months corrected age; to explore the relationship between developmental outcome measures and maternal stress in the two subgroups; to analyze the influence of neonatal risk factors and maternal sociodemographic characteristics on developmental outcome measures.	Living and working conditions; characteristics of the individual.	Mothers and 79 PTNB* - 39 late preterm infants (born between 33-36 weeks of GA ^†^ ) - 40 very preterm infants (born ≤ 32 weeks of GA ^†^ ).
A08 ^( 29 )^	Neurodevelopmental profile, growth, and psychosocial environment of preterm infants with difficult feeding behavior at age 2 years	Cohort, United States, (2015).	To examine the association of difficult feeding behaviors in very preterm infants at two years of age with growth and neurodevelopmental outcomes and family factors and functioning.	Living and working conditions; social and support networks in the community; characteristics of the individual.	104 PTNB* (born at ≤ 30 weeks of GA ^†^ ).
A09 ^( 30 )^	Developmental outcomes of extremely preterm infants born to adolescent mothers	Retrospective cohort, United States, (2015).	To evaluate the cognitive, language, and behavioral outcomes of extremely preterm infants born to adolescent mothers (< 20 years) compared to extremely preterm infants born to adult mothers (≥ 20 years); to identify the socioeconomic risk factors that affect outcomes.	Economic, social, cultural, and environmental conditions; living and working conditions; social and support networks in the community; behavioral and lifestyle factors of the individual; characteristics of the individual.	1,934 PTNB* (born at 27 weeks) - 211 PTNB* of teenage mothers - 1,723 PTNB* of adult mothers
A10 ^( 31 )^	Health care use outcomes of an integrated hospital-to-home mother-preterm infant intervention	Randomized controlled trial, United States, (2016).	To compare health care from hospital discharge to six weeks of corrected age in two groups of preterm mother-baby dyads: those who received an intervention, Hospital to Home: Optimizing Premature Infant’s Environment (H-HOPE) ^††^ and a care control group.	Economic, social, cultural and environmental conditions; living and working conditions; social and support networks in the community; characteristics of the individual.	147 mothers and PTNB* (born between 29 and 34 weeks of GA ^†^ ).
A11 ^( 32 )^	Life after discharge: what parents of preterm infants say about their transition to home	Descriptive and exploratory, United States, (2016).	To explore and describe the experiences of parents of premature babies after discharge from hospital.	Economic, social, cultural, and environmental conditions; living and working conditions; social and support networks in the community; behavioral and lifestyle factors of the individual; characteristics of the individual.	52 parents of PTNB* (born < 37 weeks).
A12 ^( 33 )^	A qualitative study: mothers of late preterm infants relate their experiences of community-based care	Descriptive with a phenomenological approach, Canada, (2017).	To explore mothers’ experience of caring for their late preterm babies in the community.	Economic, social, cultural and environmental conditions; living and working conditions; social and support networks in the community; behavioral and lifestyle factors of the individual; characteristics of the individual.	11 mothers of PTNB* (born between 34 and 37 weeks of GA ^†^ ).
A13 ^( 34 )^	Homecare and healthcare utilization errors post–neonatal intensive care unit discharge	Cohort, United States, (2017).	To identify and describe home care and health care utilization errors in high-risk babies after discharge from the NICU ^‡‡^ .	Economic, social, cultural, and environmental conditions; living and working conditions; social and support networks in the community; behavioral and lifestyle factors of the individual; characteristics of the individual.	241 PTNB* - 163 born with <32 weeks of GA ^†^ or 1,500 grams - 78 born with > 32 weeks of GA ^†^ and more than 1500 grams
A14 ^( 35 )^	*Situación socioeconómica familiar y neurodesarrollo de prematuros de muy bajo peso al nacer a los dos años de edad*	Retrospective cohort, Chile, (2018).	To characterize the families where very low birth weight premature infants with low and medium-low socioeconomic status grow up; to explore the possible association between socio-family characteristics and neurodevelopment at two years of age.	Economic, social, cultural and environmental conditions; living and working conditions; social and support networks in the community; behavioral and lifestyle factors of the individual; characteristics of the individual.	162 families of very low birth weight PTNB* (born < 32 weeks of GA ^†^ or < 1500 grams).
A15 ^( 36 )^	*Intervención basada en rutinas como apoyo a la participación familiar para el desarrollo del lenguaje en bebés prematuros*	Case study, Ecuador, (2020).	To present the impact that a Routine-Based Intervention (RBI) ^§§^ can have on family participation in the language development of their premature babies.	Economic, social, cultural, and environmental conditions; living and working conditions; social and support networks in the community; behavioral and lifestyle factors of the individual; characteristics of the individual.	11 families and 11 PTNB* (born at < 37 weeks of GA ^†^ ).
A16 ^( 37 )^	Disparities in preterm infant emergency room utilization and rehospitalization by maternal immigrant status	Exploratory, United States, (2020).	To evaluate the effects of immigrant motherhood and risk factors on the rates of emergency room visits and readmissions of premature babies up to 90 days after discharge.	Economic, social, cultural, and environmental conditions; living and working conditions; social and support networks in the community; behavioral and lifestyle factors of the individual; characteristics of the individual.	732 mothers and 866 PTNB* (born at < 37 weeks of GA ^†^ ). - 176 immigrant mothers and 203 PTNB*. - 556 native mothers and 663 PTNB*.
A17 ^( 38 )^	Home visiting for Neonatal Intensive Care Unit (NICU) ^‡‡^ graduates: impacts of Following Baby Back Home	Cohort, United States, (2021).	To examine whether the Following Baby Back Home (FBBH) ^||||^ intervention, compared to the control group, demonstrated increased health care and immunizations and lower infant mortality from discharge from the NICU ^‡‡^ until the first year of life.	Economic, social, cultural, and environmental conditions; living and working conditions; social and support networks in the community; behavioral and lifestyle factors of the individual; characteristics of the individual.	498 PTNB* low birth weight (< 2500 grams and < 37 weeks of GA ^†^ ).
A18 ^( 39 )^	The effects of prematurity and socioeconomic deprivation on early speech perception: a story of two different delays	Longitudinal, United Kingdom, (2020).	To understand the interaction between physical/cognitive maturation and environmental exposure to language in the early language development.	Economic, social, cultural, and environmental conditions; living and working conditions.	38 PTNB* (born ≤ 33 weeks of GA ^†^ ).
A19 ^( 40 )^	Maternal post-traumatic stress and depression symptoms and outcomes after NICU ^‡‡^ discharge in a low-income sample: a cross-sectional study	Transversal, United States, (2021).	To describe the prevalence of positive tests for acute post-traumatic stress (PTS) ^¶¶^ and symptoms of depression among low-income families after discharge from the NICU ^‡‡^ . To examine the adjusted association of PTS ^¶¶^ and symptoms of depression and child health and neurodevelopmental outcomes. To evaluate the adjusted association of PTS ^¶¶^ and symptoms of depression and maternal quality of life.	Economic, social, cultural, and environmental conditions; living and working conditions; characteristics of the individual.	150 families of PTNB* (born < 37 weeks of GA ^†^ ).
A20 ^( 41 )^	The association of care transitions measure-15 score and outcomes after discharge from the NICU ^‡‡^	Transversal, United States, (2021).	To describe Care Transitions Measure (CTM)*** scores among caregivers of premature infants after discharge from the NICU ^‡‡^ . To describe the association of CTM*** scores with readmissions, enrollment in public assistance programs, and caregiver quality of life scores.	Economic, social, cultural, and environmental conditions; living and working conditions; characteristics of the individual.	169 parents of PTNB* (born with <37 weeks of GA ^†^ ).
A21 ^( 42 )^	Factors associated with the socioemotional development of preterm infants	Longitudinal observational, Brazil, (2021).	To compare the difference in socio-emotional development between hospital discharge and at six months of corrected age of infants born ≤ 32 weeks and to evaluate the impact of maternal and infant factors on the rate of socio-emotional development over a six-month period.	Economic, social, cultural, and environmental conditions; living and working conditions; social and support networks in the community; behavioral and lifestyle factors of the individual; characteristics of the individual.	72 mothers and PTNB* (born at ≤ 32 weeks of GA ^†^ ).
A22 ^( 11 )^	Maternal depressive symptoms, poverty, and young motherhood increase the odds of early depressive and anxiety disorders for children born prematurely	Longitudinal observational, United States, (2021).	To identify the prevalence of depressive and anxiety disorders at two years of age among children born prematurely and to determine the extent to which poverty, maternal depressive symptoms, or young motherhood increase the likelihood of these disorders.	Economic, social, cultural, and environmental conditions; living and working conditions; social and support networks in the community; behavioral and lifestyle factors of the individual; characteristics of the individual.	105 mothers and PTNB* (born at < 37 weeks of GA ^†^ ).
A23 ^( 43 )^	Risk factors for re-hospitalization following neonatal discharge of extremely preterm infants in Canada	Prospective cohort, Canada, (2021).	To identify the neonatal, sociodemographic, and geographic characteristics that predict readmission in extremely preterm newborns from newborns.	Economic, social, cultural, and environmental conditions; living and working conditions; characteristics of the individual.	2,275 PTNB* (born between 22 and 28 weeks of GA ^†^ ).
A24 ^( 44 )^	Risk factors for fine and gross motor development in preterm and term infants	Observational, Brazil, (2021).	To investigate the association of sociodemographic, obstetric, and psychosocial factors with delayed fine and gross motor development in preterm and term infants aged between three months and one day and 12 months and 29 days.	Economic, social, cultural and environmental conditions; living and working conditions; social and community support networks; behavioral and lifestyle factors of the individual; characteristics of the individual.	Term and preterm NB ^‡^ (< 37 weeks). Stage 1 (3 - four months and 29 days): 103 term and 62 PTNB* Stage 2 (8 - nine months and 29 days): 84 term and 46 PTNB* Stage 3 (11 - 12 months and 29 days): 69 term and 33 PTNB*.
A25 ^( 45 )^	Parent-reported health status of preterm survivors in a Canadian cohort	Prospective cohort, Canada, (2022).	To describe the health status reported by parents in a large national cohort of children born extremely prematurely at pre-school age and to identify the clinical and sociodemographic variables associated with health status.	Social and support networks in the community; behavioral and lifestyle factors of the individual; characteristics of the individual.	811 PTNB* (born < 29 weeks of GA ^†^ ).
A26 ^( 14 )^	Neurodevelopmental outcomes at 6, 12, and 24 months of age in preterm infants with very low birth weights in Taiwan	Cohort, Taiwan, (2021).	Identify the perinatal history associated with neurodevelopmental impairment for very low birth weight preterm infants at 6, 12, and 24 months of age and the stability of neurodevelopmental assessments.	Economic, social, cultural and environmental conditions; living and working conditions; social and support networks in the community; characteristics of the individual.	8,517 very low birth weight PTNB* (between 24 and 32 weeks of GA ^†^ ).
A27 ^( 46 )^	Association of inotrope use with neurodevelopmental outcomes in infants <29 weeks gestation: a retrospective cohort study	Retrospective cohort Canada, (2021).	Comparing neurodevelopmental outcomes at 18-24 months in preterm infants’ development outcomes at 18-24 months in preterm infants <29 weeks gestational age who received versus those who did not receive inotropes in the first week of life.	Living and working conditions; individual behavioral and lifestyle factors; individual characteristics.	2,615 PTNB* (<29 weeks of GA ^†^ ).
A28 ^( 47 )^	Association of preterm birth and socioeconomic status with neonatal brain structure	Cohort, United Kingdom, (2023).	To investigate associations between gestational age at birth and socioeconomic status with neonatal brain morphology.	Economic, social, cultural and environmental conditions; living and working conditions; behavioral and lifestyle factors of the individual; characteristics of the individual.	170 PTNB* (< 33 weeks of GA ^†^ ) and 91 term NB ^‡^ .
A29 ^( 48 )^	Cortisol reactivity and negative affect among preterm infants at 12 months during a mother-infant interaction task	Longitudinal, Portugal, (2023).	To investigate the correlations between the cortisol reactivity of premature babies and the association with the baby’s negative affect.	Economic, social, cultural, and environmental conditions; living and working conditions; behavioral and lifestyle factors of the individual; characteristics of the individual.	48 PTNB*
A30 ^( 49 )^	Early developmental support for preterm infants based on exploratory behaviors: a parallel randomized controlled study	Randomized controlled trial Turkey, (2023).	To investigate the effectiveness of the new Explorer Baby early intervention program on the motor, cognitive, and language skills of premature babies.	Living and working conditions; individual behavioral and lifestyle factors; individual characteristics.	57 PTNB* (< 33 weeks of GA ^†^ ).
A31 ^( 50 )^	Language outcomes at 18–24 months of preterm infants from US Spanish- and English-speaking homes	Retrospective cohort United States, (2023).	To evaluate the association between mild to moderate expressive and receptive language delay and the main language (Spanish and English) spoken at home by preterm infants using the BSID-III** and to examine the association with socioeconomic factors.	Economic, social, cultural, and environmental conditions; living and working conditions; social and support networks in the community; behavioral and lifestyle factors of the individual; characteristics of the individual.	1,125 PTNB* (< 32 weeks of GA ^†)^ .

*PTNB = Preterm Newborn; ^†^GA = Gestational Age; ^‡^NB = Newborn; ^§^IBAIP = Infant Behavioral Assessment and Intervention Program; ^||^NBRS = Neurobiologic Risk Score; ^¶^NRE = Neurodevelopmental Risk Exam; **BSID-III = Bayley Scales of Infant and Toddler Development, Third Edition; ^††^H-HOPE = Hospital to Home: Optimizing Premature Infant’s Environment; ^‡‡^NICU = Neonatal Intensive Care Unit; ^§§^IBR = Intervention Based on Routines; ^||||^FBBH = Following Baby Back Home; ^¶¶^PTS = Post-Traumatic Stress; ***CTM = Care Transitions Measure

The process of coding the studies made it possible to identify the variables congruent with the SDH dimensions (Table 1) according to the codes and subcodes described in the data analysis section.

The articles covered all the SDH macro-dimensions: living and working conditions, behavioral and lifestyle factors, and characteristics of the individual concerning children born preterm in the first two years of life, the latter being explored by all the articles.

**Table 1 -:** Variables by subcode and quantified for the studies. Belo Horizonte, MG, Brazil, 2024

**Subcode**	n*	**%** ^†^ (n*/total)
**Economic, social, cultural and environmental conditions**	**22**	**70.97%** ^†^
Socio-economic factors	18	58.06% ^†^
Cultural factors	15	48.39% ^†^
Environmental factors	0	0.00% ^†^
Political factors and public policies	7	22.58% ^†^
**Living and working conditions**	**29**	**93.55%** ^†^
Access to food	0	0.00% ^†^
Access to essential services	14	45.16% ^†^
Education	27	87.10% ^†^
Housing conditions	10	32.26% ^†^
Working conditions	10	32.26% ^†^
**Social and community support networks**	**21**	**67.74%** ^†^
Support networks	21	67.74% ^†^
**Individual behavioral and lifestyle factors**	**19**	61.29% ^†^
Family care	12	38.71% ^†^
Breastfeeding	7	22.58% ^†^
Unhealthy habits	8	25.81% ^†^
**Characteristics of the individual**	**31**	**100%** ^†^
Health condition	31	100% ^†^
Age, sex, and hereditary factors	31	100% ^†^
Psychological factors	15	48.39% ^†^
Ethnic and racial factors	16	51.61% ^†^
**Child health outcomes**	**31**	**100%** ^†^
Development	19	61.29% ^†^
Behavioral	5	16.13% ^†^
Growth	4	12.90% ^†^
Diseases	5	16.13% ^†^
Infant mortality	1	3.23% ^†^
Demand for health services	9	29.03% ^†^

*n = Number; ^†^% = Percentage of studies

The variables investigated in the articles analyzed are represented specifically according to the macro and micro dimensions of SDH in Table 2.

**Table 2 -:** Variables covered in the studies and children’s health outcomes related to the codes and subcodes. Belo Horizonte, MG, Brazil, 2024

**Subcode**	**Articles**	Qty* (n ^†^ **)**	**%** ^‡^ **(n** ^†^ **/total)**
**Socio-economic factors**			
Poverty	A05, A15, A16, A22, A23	5	16.13% ^‡^
Family income	A05, A10, A11, A12, A15, A17, A18, A19, A20, A22, A24, A28, A29	13	41.94% ^‡^
Socio-economic conditions	A14, A18, A21, A26, A28	5	16.13% ^‡^
Durable consumer goods	A05	1	3.23% ^‡^
Socio-demographic risk index	A05	1	3.23% ^‡^
**Cultural factors**			
Nationality	A11, A12, A16, A22, A23, A24	6	19.35% ^‡^
Mother tongue	A02, A09, A10, A13, A16, A18, A19, A20, A31	9	29.03% ^‡^
Quality of life	A19, A20, A28	3	9.68% ^‡^
**Political factors and public policies**			
Transitional care program	A11	1	3.23% ^‡^
Food supplementation program	A17, A20	2	6.45% ^‡^
Social benefits	A14, A20, A22	3	9.68% ^‡^
Child protection service	A16	1	3.23% ^‡^
Domestic abuse	A16	1	3.23% ^‡^
State supervision	A09	1	3.23% ^‡^
**Access to essential services**			
Access to health services (prenatal, maternity, intervention, specialization)	A09, A11, A16, A20, A24, A31	6	19.35% ^‡^
Access to health intervention	A02, A10, A13, A17, A20, A30, A31	7	22.58% ^†^
Social assistance	A23	1	3.23% ^‡^
Health insurance (public or private)	A09, A11, A16, A17, A31	5	16.13% ^‡^
Access to specialized care or outpatient follow-up	A09, A10, A03, A12, A31	5	16.13% ^‡^
Post-discharge follow-up in the NICU ^§^	A13	1	3.23% ^‡^
**Education**			
Level of education of caregivers	A02, A03, A04, A05, A06, A07, A08, A09, A10, A11, A12, A13, A14, A16, A17, A18, A19, A20, A22, A23, A24, A26, A27, A28, A29, A30, A31	27	87.10% ^‡^
**Housing condition**			
Place of residence/housing	A09, A10, A14, A17, A23, A28	6	19.35% ^‡^
Number of inhabitants/children in the home/overcrowding	A09, A10, A14, A16, A23, A24, A29	7	22.58% ^†^
Distance between place of residence and health service	A23	1	3.23% ^‡^
Foster home	A08	1	3.23% ^‡^
**Working conditions**			
Employment situation	A08, A10, A14, A15, A23, A24, A25, A27, A28, A29	10	32.26% ^‡^
Provider’s occupation	A08	1	3.23% ^‡^
**Support networks**			
Caregiver’s marital status	A02, A03, A05, A06, A08, A09, A11, A12, A13, A14, A16, A17, A21, A24, A25, A26, A27, A31	18	58.06% ^‡^
Family composition/functioning	A09, A22	2	6.45% ^‡^
Support from family/friends	A05, A09, A12, A15	4	12.90% ^‡^
Type of caregiver (paid/babysitting or primary, daycare)	A09, A11, A14, A21	4	12.90% ^‡^
Social support	A10, A24	2	6.45% ^‡^
**Family care**			
Organization/routine of care	A11, A15, A24, A30	4	12.90% ^‡^
Maternal support for solving problems for the child	A05	1	3.23% ^‡^
Inadequate care related to consultation, administration of medication, feeding, use of equipment	A13	1	3.23% ^‡^
Family and professional care (e.g. day care, home care, professional caregiver or nanny)	A09, A31	2	6.45% ^‡^
Kangaroo care	A21, A30	2	6.45% ^‡^
Attachment (mother-infant interaction)	A02, A05, A21, A22, A29, A30	6	19.35% ^‡^
**Breastfeeding**			
Breastfeeding situation	A01, A16, A17, A21, A28, A31	6	19.35% ^‡^
Breastfeeding challenges (e.g. lactation problems, latch-on, others)	A11	1	3.23% ^‡^
Breastfeeding at hospital discharge	A16	1	3.23% ^‡^
**Unhealthy habits**			
Alcohol consumption A12, A14 2 6.45% ^‡^	A12, A14	2	6.45% ^‡^
Use of substances/medications during pregnancy A16, A24, A25 3 9.68% ^‡^	A16, A24, A25	3	9.68% ^‡^
Smoking A01, A17, A28 3 9.68% ^‡^	A01, A17, A28	3	9.68% ^‡^
Health condition			
Birth weight	A01, A02, A03, A04, A05, A06, A07, A08, A09, A10, A11, A12, A13, A14, A16, A17, A18, A19, A20, A21, A23, A24, A26, A27, A28, A29, A30, A31	28	90.32% ^‡^
Anthropometric measurements (weight, height, head circumference)	A08, A14, A28, A31	4	12.90% ^‡^
Changes in growth	A19, A20, A26	3	9.68% ^‡^
Apgar score	A01, A05, A06, A10, A12, A17, A26, A27, A29, A31	10	32.26% ^‡^
Route of delivery	A01, A06, A07, A12, A24	5	16.13% ^‡^
Degree of prematurity	A16, A31	2	6.45% ^‡^
Neonatal transfer after birth	A26	1	3.23% ^‡^
Jaundice	A12	1	3.23% ^‡^
Twinning	A02, A04, A05, A06, A07, A08, A10, A11, A15, A16, A23, A28, A29	13	41.94% ^‡^
Use of devices (trachea/gastrostomy, walking aid, use of oxygen and mechanical ventilation, CPAP ^||^ )	A03, A05, A14, A17, A19, A20, A23, A24, A26, A29	10	32.26% ^‡^
Gastroesophageal reflux	A29	1	3.23% ^‡^
Birth order	A02, A05	2	6.45% ^‡^
Resuscitation at birth	A26	1	3.23% ^‡^
Use of oxygen	A02, A14, A16, A19, A20, A23, A29, A31	8	25.81% ^‡^
Sepsis	A02, A04, A07, A09, A12, A14, A16, A26, A27	9	29.03% ^‡^
Neurological injury (e.g. intracerebral and/or intraventricular and/or periventricular hemorrhage; periventricular leukomalacia; cerebral palsy)	A02, A03, A04, A06, A07, A08, A09, A14, A16, A17, A19, A20, A21, A23, A25, A26, A27, A31	18	58.06% ^‡^
Days alive at discharge	A13	1	3.23% ^‡^
Circulatory compromise	A26	1	3.23% ^‡^
Duration of mechanical ventilation	A09	1	3.23% ^‡^
Blood pH	A03	1	3.23% ^‡^
Seizures	A03, A14, A17, A26, A31	5	16.13% ^‡^
Hypothermia	A12,	1	3.23% ^‡^
Hypoglycemia	A03, A12	2	6.45% ^‡^
Neurodevelopmental impairment	A03, A25	2	6.45% ^‡^
Neurological assessment	A03, A31	2	6.45% ^‡^
Blindness	A04, A31	2	6.45% ^‡^
Motor function	A04, A30	2	6.45% ^‡^
Length of stay	A05, A07, A09, A13, A15, A16, A21, A23, A24, A26, A27, A29, A30, A31	14	45.16% ^‡^
Respiratory changes (apnea, respiratory disease, respiratory problem, respiratory distress syndrome, chronic lung disease, bronchopulmonary dysplasia, surfactant deficiency)	A04, A05, A06, A07, A09, A12, A16, A17, A19, A20, A23, A25, A26, A27, A29, A30, A31	17	54.84% ^‡^
Cardiovascular changes	A07, A12, A17	3	9.68% ^‡^
ROP ^¶^ /ROP ^¶^ III-V	A07, A09, A15, A19, A20, A25, A26, A27	8	25.81% ^‡^
Health condition at hospital discharge	A05, A06, A07, A25	4	12.90% ^‡^
Type of diet	A24	1	3.23% ^‡^
Feeding difficulties	A08, A12, A24	3	9.68% ^‡^
Pre/postnatal steroid use	A01, A04, A06, A09, A26, A27	6	19.35% ^‡^
Hydrocephalus	A14, A26, A31	3	9.68% ^‡^
Chromosomal abnormality	A17	1	3.23% ^‡^
Demand for health services (≥ 2 appointments/month, routine care or illness, hospital readmission, surgeries, length of readmission, post-discharge diagnosis)	A10, A11, A17, A19, A20	5	16.13% ^‡^
Feeding by gavage	A19, A20, A23	3	9.68% ^‡^
Level of cognitive and motor development	A22, A30, A31	1	3.23% ^‡^
Level of language ability	A30, A31	2	6.45% ^‡^
Mortality	A27	1	3.23% ^‡^
Neonatal or infant morbidity	A10, A22, A26	3	9.68% ^‡^
Infectious changes (infection, pneumonia, enterocolitis)	A03, A09, A14, A16, A19, A20, A23, A26, A27, A31	10	32.26% ^‡^
Hearing loss/deafness	A04, A31	1	3.23% ^‡^
Maternal diseases and obstetric history	A01	1	3.23% ^‡^
Chronic diseases	A01	1	3.23% ^‡^
Premature rupture of membranes	A09, A26	2	6.45% ^‡^
Premature placental abruption	A26	1	3.23% ^‡^
Parity	A01, A09, A10, A12, A13, A16, A21, A24, A26, A31	10	32.26% ^‡^
Miscarriage	A24	1	3.23% ^‡^
Pre- and post-partum complications	A24	1	3.23% ^‡^
Hypertension	A06, A09, A26, A27, A31	5	16.13% ^‡^
Diabetes Mellitus/Gestational Diabetes Mellitus	A26, A27	2	6.45% ^‡^
Pre-eclampsia	A09, A26	2	6.45% ^‡^
Chorioamnionitis	A06, A09, A26, A27, A31	5	16.13% ^‡^
Restricted intrauterine growth	A26	1	3.23% ^‡^
Route of delivery	A09, A26, A24, A27, A31	5	16.13% ^‡^
Antenatal administration of corticosteroids	A09, A23, A26, A27	4	12.90% ^‡^
Twinning	A09, A12, A26, A27	4	12.90% ^‡^
Prepartum hemorrhage	A26	1	3.23% ^‡^
Placenta previa	A26	1	3.23% ^‡^
Prenatal magnesium sulfate administration	A27	1	3.23% ^‡^
Assisted reproductive technology	A27	1	3.23% ^‡^
Oligohydramnios and polyhydramnios	A26	1	3.23% ^‡^
**Age, sex, and hereditary factors**			
Gestational age	A01, A02, A03, A04, A05, A06, A07, A08 A09, A10, A11, A12, A13, A14, A15, A16, A17, A18, A19, A20, A21, A22, A23, A24, A25, A26, A27, A28, A29, A30, A31	31	100% ^‡^
Sex	A02, A03, A04, A05, A06, A07, A08, A09, A10, A12, A13, A14, A15, A16, A17, A18, A21, A22, A23, A24, A25, A26, A27, A28, A29, A30, A31	27	87.10% ^‡^
**Psychological factors**			
Depression and anxiety	A08, A22	2	6.45% ^‡^
Anxiety/trait	A01, A08, A10, A12, A21, A29	5	16.13% ^‡^
Depression/symptoms	A01, A05, A08, A10, A12, A19, A21, A22, A29	8	25.81% ^‡^
Stress/post-traumatic stress	A01, A02, A05, A07, A08, A11, A12, A19, A29, A30	10	32.26% ^‡^
Fatigue/exhaustion	A11	1	3.23% ^‡^
Guilt	A11	1	3.23% ^‡^
Difficulty sharing feelings	A11	1	3.23% ^‡^
Social isolation/loneliness	A11	1	3.23% ^‡^
Mental health diagnosis	A16,	1	3.23% ^‡^
Psychological factors (history of depression, planned pregnancy, feelings about motherhood)	A24	1	3.23% ^‡^
Psychological distress	A02	1	3.23% ^‡^
**Ethnic and racial factors**			
Race	A04, A05, A09, A10, A13, A16, A17, A19, A20, A22, A31	11	35.49% ^‡^
Ethnicity	A02, A04, A05, A08, A09, A10, A12, A13, A16, A17, A19, A20, A22, A23, A28, A31	16	51.61% ^‡^
**Children’s health outcomes**			
**Development**	-	**19**	-
Cognitive	A03, A04, A06, A07, A08, A09, A19, A25, A27, A30, A31	11	-
Linguistic	A04, A06, A07, A08, A09, A18, A25, A30, A31	9	-
Motor	A07, A08, A09, A19, A24, A25, A27	7	-
Neurological	A09, A14, A25	3	-
Socio-emotional	A08, A09, A21, A22, A29	5	-
General	A15	1	-
Communication, socialization and daily living skills	A19, A30, A31	3	-
Mental development	A26	1	-
Psychomotor development	A26	1	-
Brain	A28	1	-
**Behavioral**		**5**	-
Children’s behavior	A02, A05, A08, A09, A21	5	-
**Growth**		**4**	-
Head circumference	A09	1	-
Growth/weight	A09, A11, A12, A13	4	-
**Affections**		**5**	-
Eating difficulties	A08	1	-
Cerebral palsy	A09	1	-
Visual or sensorineural/auditory impairments	A09, A25, A27	3	-
State of health (healthy or not)	A01	1	-
**Infant mortality**	A17	1	-
**Demand for health services** (routine consultation, immunization, readmission, emergency, emergency room or intensive care)	A09, A10, A11, A13, A16, A17, A19, A20, A23	9	-

*Qty = Quantity; ^†^n = Number; ^‡^% = Percentage of studies; ^§^NICU = Neonatal Intensive Care Unit; ^||^CPAP = Continuous Positive Airway Pressure; ^¶^ROP = Retinopathy of Prematurity

Regarding the Individual Characteristics dimension, all the studies investigated “Health Conditions”, with emphasis on the birth weight variable, which was covered in 28 articles (90.32%). All the studies also used the variables to investigate the Age, Sex, and Hereditary Factors dimension. In this dimension, the least addressed aspects were the “psychological factors” in 15 studies (48.39%) and the “Ethnic and Racial Factors” addressed in 16 studies (51.61%).

As for the Living and Working Conditions dimension, 14 articles (45.16%) dealt with aspects related to “access to essential services”, while 10 (32.26%) dealt with “housing conditions”. Variables were also identified for Behavioral and Individual Lifestyle Factors, with seven (22.58%) articles addressing “breastfeeding” and 12 (38.71%) addressing “family care”. No variables were identified to explore the dimensions of SDH relating to “environmental factors” and “access to food”.

The child health outcomes investigated (Table 2) were mostly (n=19) related to development, measured using different scales, such as the Bayley Scales of Infant and Toddler Development, Third Edition (BSID-III) (linguistic, cognitive and motor development), Cognitive Adaptive Test/Clinical Linguistic and Auditory Milestone Scale (CAT/CLAMS) (cognitive development), DENVER (motor development), Gross Motor Function Classification System (GMFCS) and Bayley Scales of Infant Development, Second Edition (BSID-II) (neurological development), Brief Infant-Toddler Social and Emotional Assessment (BITSEA) and Mother-baby Interaction Observation Protocol (POIMB) (socio-emotional/behavioral development), Ages and Stages Questionnaires 3 (ASQ-3) (general development). Of the total number of studies, only four investigated growth-related outcomes (weight, head circumference, and growth).

## Discussion

The analysis of the studies made it possible to highlight the aspects that are being addressed in scientific productions that contribute to knowledge about children born preterm in the first two years of life and that consider the dimensions of SDH^(6)^.

Although the studies address variables that contribute to learning about SDH, none use the theoretical framework^(6)^ for their development. The use of this framework can contribute to understanding children’s ways of living and becoming ill. It is known that understanding the context in which the child is inserted is fundamental for a comprehensive approach, enables a broader view, and allows for the formulation of more effective individualized care and the planning of public policies.

Concerning the Economic, Social, Cultural, and Environmental Conditions dimension, the socioeconomic factor variable was addressed in 18 articles, seeking to measure family income, poverty, and socioeconomic conditions in particular. One of the studies analyzed pointed out the importance of developing strategies to mitigate the adverse effects of unfavorable socioeconomic family levels, which had a direct impact on the neurodevelopment of premature children and their educational outcomes^(47)^. Furthermore, unfavorable social conditions such as low family income and poverty were associated with symptoms of anxiety and greater symptoms of depression, respectively^(11)^, influencing the process of child illness.

Cultural factors were predominantly examined based on language and nationality, showing a greater chance of readmission and length of hospital stay among the children of immigrant mothers compared to those of native mothers^(37)^. It is known that people who live as immigrants can be exposed to situations of vulnerability and insecurity, and in this context of care, their children can present a greater health risk.

Issues related to political factors and public policies were identified as social benefits, protection programs, and services, among others. These programs and social benefits help with access to information and have a positive impact on children’s health, as demonstrated by the Following Baby Back Home program, which optimizes the search for health care, reducing mortality rates for preterm children with low birth weight^(38)^. On the other hand, state supervision, which has been pointed out as a predominant indicator of abuse and neglect among teenage mothers, has succeeded in increasing the risks of adverse cognitive, language, and motor effects^(30)^.

Although the studies in this review did not identify aspects related to access to food, research has shown that food-insecure families need at least two minimum wages to meet their monthly expenses^(51)^. Exploring the socio-environmental conditions to which children are subjected is an aspect that needs to be explored in further studies since these determinants can modify growth and development in prematurity^(17)^. Vulnerability related to sanitation conditions and access to food increases the risk of malnutrition, and gastrointestinal infections and, consequently, increases the need to go to the health service^(52-53)^.

Following on from the Living and Working Conditions dimension, access to essential services was investigated from the perspective of health insurance, access to interventions, and services such as maternity care and outpatient follow-up. Special services, such as the home visit program or access to social services, were pointed out as great allies for the health of children born preterm since they increase health care, reducing the risk of mortality and mitigating adverse effects on development^(30,38)^. Limited access to health services can be related to issues within the system itself, such as a shortage of professionals and inadequate organization, as well as social issues such as schooling and socioeconomic factors^(54)^. Therefore, it is inferred that professionals must identify the barriers that may hinder the family’s access to services from prenatal care onwards to develop strategies to improve perinatal outcomes.

Thus, it is known that children discharged from the NICU, especially preterm children, are potentially at risk of altered growth and development and have an increased need to visit health services than usual due to complications^(12,31)^. Therefore, post-discharge follow-up in outpatient services must be well established, to provide a multidisciplinary assessment of the child and, consequently, minimize possible damage through early identification of alterations and care guidelines^(12)^.

Education has shown important outcomes, such as the impact of maternal schooling on the neurodevelopmental levels of children born preterm, especially cognitive and language development^(27)^. Another study in the literature identified that economic levels and the availability of resources in the home environment, such as toys and activities carried out with parents, improved cognitive development between 24 and 42 months^(55)^. These results reinforce the importance of exploring the child’s environment.

Regarding how children live, studies that explore housing^(43)^ and family organization for care^(44)^ stand out. Concerning housing conditions, the studies in this review found that the number of inhabitants in the home increased the chances of readmission^(43)^, which may be associated with the transmission of diseases. On the other hand, the presence of more people in the household and having siblings was associated with a lower chance of delays in fine and gross motor skills, respectively^(44)^, since other family members stimulate the child’s development^(36)^. However, when it comes to the mother’s working conditions, the child being cared for by a mother who has some kind of professional activity was associated with a greater chance of the child having delayed fine motor skills^(44)^.

In this scoping review, the Social and Community Support Networks dimension covered 21 articles and identified components such as marital status, family composition, support from family/friends, and paid caregiver, among others. The importance of the support network is noteworthy, as it supports the mother and consequently provides favorable contexts for child development^(36)^. In addition, maternal family figures such as grandparents are seen as knowledge references based on their experiences and can be influenced by their beliefs and values^(56)^.

The Behavioral and Lifestyle Factors dimension was the least explored by the studies. These results may have been influenced by the home context of the investigations, however, the literature shows that support for kangaroo care through home visits was related to a reduction in neonatal and infant mortality among low birth weight babies^(57)^. In addition, children born preterm at an early age present risks for the favorable establishment of breastfeeding^(22)^, increasing the chances of early weaning, which can be circumvented through guidance on the initiation and establishment of breastfeeding^(57-58)^.

Family care interferes with the child’s development and proper growth and can expand or limit health promotion according to the SDH present in the family’s reality^(36)^. Therefore, considering the family care variable in future research could provide a better understanding of the feelings experienced and the challenges faced by caregivers, highlighting the need to develop support strategies for families^(59)^. Care can also be investigated from the perspective of a professional caregiver in educational institutions such as day-care centers which, combined with family care at home, favor child development^(55)^.

Attachment refers to the bonds established between caregivers and children, found in this review as maternal-child interaction. Some studies have shown that the interaction was influenced by the mother’s emotional health in cases of stress, anxiety, and depression, leading to less stimulation for the child and, consequently, an increase in developmental risks^(10,26)^. Given these impacts, further research into attachment is essential, enabling the identification of risk factors where professionals can create strategies to guide and support families, and protective factors that support healthy child growth and development.

Concerning unhealthy habits, this review showed that children living with someone who consumes alcohol abusively are five times more likely to have delayed neurodevelopment^(35)^. Considering that these habits are modifiable factors, one study pointed to the need to develop policies focused on reducing alcohol and tobacco consumption, since these have been associated with inadequate development in early childhood^(60)^.

All the studies investigated the Individual Characteristics dimension, using the variables age, sex, and hereditary factors; health conditions often associated with obstetric history, birth condition, growth, development, and needs for health services. The impairments caused by these variables have the potential to increase the demand for care throughout the child’s life. Thus, identifying the variables being studied allows us to understand the impact of each of them, such as the association between longer hospital stays and impaired socio-emotional, cognitive, and motor development^(42,61)^.

Another point that needs to be discussed in the context of prematurity is the approach to psychological factors, dealt with in 15 articles. Motherhood is a phase of vulnerability. When added to prematurity, this context imposes challenges on the family and especially on the mother, as it becomes a risk factor for cases of depression^(10)^. In addition, the limited support provided by professionals to these families after discharge results in fatigue and social isolation, increasing health costs due to the avoidable complications of failed care for premature babies^(32)^. However, a literature review revealed that maternal mental suffering is not always identified and can have repercussions on the child’s overall health^(62)^. This highlights the need to adopt patient- and family-centered care to monitor potentially influential factors and establish strategies to promote well-being. One study showed that patient- and family-centered care promotes positive impacts, such as increased family satisfaction, better outcomes, and quality of life for children^(63)^.

Ethnic and racial factors expose a social gap in the face of the social inequalities intertwined with this issue, with consequences for the process of child development and growth in the face of structural socioeconomic and racial conditions^(7,64)^. Some reviewed studies have exemplified this by bringing up the association between race, adolescent mother, and language^(30)^, for example, black and Hispanic-white children have a higher risk of language delay compared to white children^(25)^.

It can be seen that the child’s health outcomes most closely related to SDH and most studied have been development, especially cognitive, linguistic, and motor development, followed by demands for health services, whether or not related to the disorders. These results reflect the importance of monitoring children in the context in which they live, especially concerning the criticality of the first two years of life due to neuroplasticity, an opportune time for interventions, and developmental stimuli^(12)^. This reinforces the need for preventive action on modifiable factors that represent a risk to full growth and development, compromising children’s integral health.

In this study, it was understood that the variables contribute to identifying the dimensions of SDH explored in scientific research, however, it is recognized that they are limited in representing these dimensions in their entirety and this was considered in the discussion of the data analyzed. In addition, the study has limitations related to the age of the children, since it excludes studies that extended the investigation to those over two years old and restricts the compression of SDH to this age group. Care settings other than the home and the inclusion of gray literature could provide important information on child health.

## Conclusion

Identifying the health of premature children using the SDH theoretical framework highlighted the protective and threatening aspects of the environment in which children live. The studies analyzed showed a greater interest in exploring variables relating to the characteristics of the individual, with emphasis on health conditions and age, sex, and hereditary factors, highlighting the search for factors that have a direct impact on children’s health. On the other hand, few studies investigated the variables of the dimensions of Economic, Social, Cultural, and Environmental Conditions, Community Social and Support Networks, and Individual Behavioral and Lifestyle Factors, indicating gaps in the understanding of SDH in child health.

It is noteworthy that the studies investigated the variables in isolation from the SDH dimensions, making it difficult to identify inequities and analyze their repercussions on the health of preterm infants. It is fundamental that future studies approach SDH in an integrated way, allowing an understanding of the living conditions of the population studied and the impacts of the way of living and getting sick of children born preterm in the first two years of life on child growth, development, and behavior. This broader approach will facilitate the development of person-centered care and allow for early interventions concerning child health outcomes.
