# Supplementary material for: Social determination in the health of children born preterm: a scoping review
Source: Rev Lat Am Enfermagem. 2025 Jul 11;33:e4605. doi: 10.1590/1518-8345.7720.4605 (PMC12259221; doi:10.1590/1518-8345.7720.4605)
Supplement: Translation

# Translation: Determinação social na saúde de crianças nascidas pré-termo: revisão de escopo ^*^

**DOI:** 10.1590/1518-8345.7720.4606

**Authors:** Mariana Fuentes Mendoza Rodrigues Soares, Amanda de Azevedo Gomes, Juliana Barony da Silva, Luciano Marques dos Santos, Patrícia Pinto Braga, Elysângela Dittz Duarte

**Affiliations:** 1 Universidade Federal de Minas Gerais, Escola de Enfermagem, Belo Horizonte, MG, Brasil.; 2 Universidade Estadual de Feira de Santana, Departamento de Saúde, Feira de Santana, BA, Brasil.; 3 Universidade Federal de São João del Rei, Curso de Enfermagem, Divinópolis, MG, Brasil.

**Keywords:** Determinantes Sociais de Saúde, Determinantes Estruturais da Saúde, Recém-Nascido Prematuro, Saúde da Criança, Crescimento e Desenvolvimento, Cuidado da Criança

## Abstract

mapear o conhecimento científico sobre as dimensões dos Determinantes Sociais de Saúde na saúde de crianças nascidas pré-termo nos primeiros dois anos de vida.

trata-se de uma *scoping review* orientada pelo *Joanna Briggs Institute* e pelo *Preferred Reporting Items for Systematic reviews and Meta-Analyses extension for Scoping Reviews*. Dos estudos encontrados em cinco bases de dados e lista de referência daqueles selecionados, foram extraídos os dados aplicando-se um instrumento de codificação elaborado a partir do modelo teórico Determinantes Sociais de Saúde com apoio do *software* MaxQDA.

foram incluídas 31 publicações. As dimensões dos determinantes sociais para a saúde da criança mais investigados foram relacionadas às características do indivíduo, com destaque para as condições de saúde e idade, sexo e fatores hereditários, realçando a busca por fatores de repercussão direta na saúde infantil.

as dimensões da saúde da criança mais exploradas foram as relacionadas ao desenvolvimento da criança. O comportamento e as condições de vida foram pouco explorados. Futuros estudos com uma abordagem ampliada dos Determinantes Sociais de Saúde de forma integrada poderão contribuir para a elaboração de cuidados centrados nos prematuros e intervenções precoce frente aos desfechos de saúde infantil.

## Destaques

**(1)** Estudos destacam a influência das características individuais na saúde infantil. **(2)** Determinantes sociais interferem no desenvolvimento e nas demandas por serviços de saúde. **(3)** Verificam-se as lacunas na investigação dos DSS e seus efeitos na saúde infantil. **(4)** Necessidade de abordar os Determinantes Sociais de Saúde de forma integrada.

## Introdução

Embora os fatores de risco para a prematuridade sejam amplamente reconhecidos^(1)^, as disparidades que contribuem para o parto prematuro e seus desfechos em saúde infantil ainda são pouco compreendidos. Um crescente corpo de evidências destaca a importância de abordar também os fatores sociais e ambientais para prevenir o parto prematuro e melhorar a qualidade de vida das famílias que têm crianças nascidas pré-termo^(2-3)^.

Além das disparidades inerentes ao nascimento pré-termo, as circunstâncias nas quais o indivíduo se encontra, como suas condições de vida e trabalho, repercutem diretamente em sua situação de saúde^(4)^, e são definidas como Determinantes Sociais de Saúde (DSS)^(5)^. Tais fatores foram esquematizados em camadas, destacando os níveis de abrangência dos DSS sobre o indivíduo e as demais camadas, podendo ser mais próximo^(6)^, sendo caracterizado pelos domínios individuais como: características individuais, sexo e fatores hereditários, ou mais externos, e os macrodeterminantes, exemplificados pelas condições econômicas, culturais e ambientais^(4)^. Esses componentes, que incluem características individuais, fatores estruturais e políticas públicas, modificam a saúde, sua exposição à doença e o acesso aos cuidados necessários^(7)^.

Sabe-se que as condições de saúde infantil podem ser afetadas pelas circunstâncias perinatais, incluindo as condições assistenciais, nutricionais, socioeconômicas e culturais, as quais influenciam os resultados do desenvolvimento a curto e longo prazo, especialmente na primeira infância^(8-9)^. Nesse contexto, o crescimento, o comportamento e o desenvolvimento infantil não são determinados apenas por mecanismos biológicos, mas também pela interação contínua entre as características individuais das crianças, dos pais e do contexto em que estão inseridas^(8-9)^. Esta interação é particularmente relevante na prematuridade, dado que a prevalência de problemas comportamentais e emocionais em crianças nascidas pré-termo está associada não só às características intrínsecas da prematuridade, mas às diversas características e comportamentos familiares^(10-11)^.

Os primeiros dois anos da criança, recorte temporal da infância definido para esta revisão, são considerados como essenciais para o crescimento e desenvolvimento de competências e habilidades, uma vez que é neste período que ocorre o maior crescimento cerebral^(12)^. Portanto, é essencial proporcionar um ambiente acolhedor, oportunidades de aprendizado, estímulos e interações que contribuam para o desenvolvimento infantil e que podem causar repercussão ao longo da vida^(12)^. Ao considerar que a prematuridade e a internação em Unidade de Terapia Intensiva Neonatal (UTIN) são fatores de risco para o crescimento e o desenvolvimento, é fundamental que as famílias recebam o acompanhamento em serviços de saúde para a vigilância à saúde e intervenções precoce, visando minimizar os possíveis prejuízos causados às crianças^(12-13)^.

Ao reconhecer a magnitude do nascimento prematuro no contexto da saúde pública global, tem-se investigado sobre as suas implicações na vida da criança e seus familiares^(13-14)^, especialmente sob as perspectivas individuais, biológicas, focadas em fatores específicos dos DSS, e na morbimortalidade infantil^(15)^. Esses achados oferecem uma compreensão limitada sobre os aspectos que podem ser modificados e contribuem ainda de forma limitada para os avanços no cuidado das crianças nascidas prematuras. Dada a complexidade da determinação social da saúde, é importante esclarecer as interferências dos fatores como: educação, vizinhança, ambiente, rede de suporte social e acesso aos serviços de saúde na vida de crianças nascidas pré-termo nos primeiros dois anos de vida. Esses fatores têm o potencial de influenciar resultados em saúde como mortalidade, morbidade, expectativa de vida, condição de saúde, gastos em saúde e limitações de função^(16)^.

Diante do exposto, parte-se do entendimento de que os domínios que se constituem como DSS oferecem uma base para uma investigação ampliada e contribuem para explorar de forma mais abrangente os efeitos do modo de viver e adoecer de crianças nascidas pré-termo nos primeiros dois anos de vida considerando o contexto ao qual estão expostas^(17)^.

A revisão de escopo busca mapear as evidências sobre a influência dos DSS na saúde de crianças nascidas pré-termo nos primeiros dois anos. Embora os fatores biológicos da prematuridade sejam bem documentados, as desigualdades sociais e estruturais ainda são pouco exploradas de forma integrada. Essa abordagem permite captar a complexidade dos DSS, identificar as lacunas no conhecimento e orientar futuras pesquisas, ampliando a compreensão sobre seu impacto no desenvolvimento e na qualidade de vida dessas crianças e suas famílias. Assim, o objetivo deste estudo foi mapear o conhecimento científico sobre as dimensões dos DSS na saúde de crianças nascidas pré-termo nos primeiros dois anos de vida.

## Método

### Tipo do estudo

Trata-se de uma *Scoping Review* orientada pelo *Joanna Briggs Institute* (JBI), *Manual for Evidence Synthesis* ^(18)^ e pelo guia internacional *Preferred Reporting Items for Systematic reviews and Meta-Analyses extension for Scoping Reviews* (PRISMA-ScR)^(19)^, conduzida em cinco etapas: elaboração da questão de pesquisa, identificação dos estudos relevantes, seleção dos estudos, análise e síntese dos dados. Esta metodologia visa mapear as evidências disponíveis e identificar lacunas de conhecimento^(20)^. Esta revisão apresenta os resultados de uma das perguntas de pesquisa elaboradas no protocolo de revisão, que foi registrado na plataforma *Open Science Framework* (OSF) e pode ser acessado pelo link https://osf.io/p724a/ com DOI 10.17605/OSF.IO/P724A.

A pergunta norteadora desta revisão foi elaborada segundo o mnemônico População, Conceito e Contexto (PCC), sendo população: crianças nascidas pré-termo nos primeiros dois anos de vida; o conceito: dimensões dos DSS; e o contexto: saúde das crianças nascidas pré-termo nos primeiros dois anos de vida.

A questão norteadora foi: Quais dimensões dos DSS foram abordadas na literatura sobre a saúde das crianças nascidas pré-termo nos primeiros dois anos de vida? Foram elaboradas questões secundárias para apoiar o alcance dos objetivos, sendo elas: Quais as abordagens metodológicas dos estudos? Quais são as características da população estudada? Quais os fatores têm sido investigados como DSS? Quais as dimensões dos DSS têm sido contempladas nos estudos? Quais os resultados em saúde da criança têm sido relacionados aos DSS?

### Critérios de seleção

Foram considerados critérios de inclusão: estudos originais em inglês, português ou espanhol, publicados nos últimos 13 anos (2011 a 2024), que considerassem crianças nascidas pré-termo nos primeiros dois anos de vida no contexto domiciliar e que abordassem os DSS e sua relação com a saúde nesse período. O recorte temporal foi definido visando mapear o conhecimento em um período mais recente, que pudesse refletir as condições de vida da população estudada. Foram excluídos os artigos de opinião, editoriais, cartas, revisões de literatura, a literatura cinzenta e os estudos que não respondiam à pergunta de pesquisa.

### Variáveis do estudo

As variáveis coletadas para a caracterização de cada estudo englobam o título, tipo de estudo, país, ano de publicação, objetivo, dimensões avaliadas e amostra. Ainda foram identificadas variáveis infantis que compreendem os códigos e subcódigos dos DSS como condições econômicas, sociais, culturais e ambientais (socioeconômicos; ambientais; culturais; políticos e políticas públicas); condições de vida e trabalho (acesso a alimentação; acesso a serviços essenciais; educação; condição de habitação; e condição de trabalho); redes sociais e de apoio da comunidade (redes de apoio); fatores comportamentais e de estilo de vida do indivíduo (cuidado familiar; cuidado canguru; apego; aleitamento materno; e hábitos não saudáveis); características do indivíduo (condições de saúde; idade, sexo e fatores hereditários; fatores psicológicos; e fatores étnicos e raciais); e resultados em saúde infantil (desenvolvimento; comportamental; crescimento; afecções; mortalidade infantil; e demandas por serviços de saúde).

### Fontes de informação e estratégia de busca

A estratégia de busca foi construída por uma bibliotecária e utilizada em cinco bases de dados, MEDLINE/*PubMed*, Biblioteca Virtual em Saúde (BVS), *Excerpta Medica Database* (EMBASE), *Scopus* e *Web of Science*. Essa etapa foi realizada em março de 2024 utilizando as estratégias de busca descritas na Figura 1.

**Figura 1 -:** Descrição das estratégias de busca utilizadas em cada base de dados. Belo Horizonte, MG, Brasil, 2024

**Base**	**Estratégia de busca**
BVS*	(“Recém-Nascido Prematuro” OR “Infant, Premature” OR “Recien Nacido Prematuro” OR prématuré OR “Bebê Prematuro” OR “Bebês Prematuros” OR prematuridade OR prematuro OR prematuros OR “Recém-Nascidos Prematuros” OR “Nascimento Prematuro” OR “Premature Birth” OR “Nacimiento Prematuro” OR “Naissance prématurée” OR “Premature Infant” OR “Premature Infants”) AND (“Determinantes Sociais da Saúde” OR “Social Determinants of Health” OR “Determinantes Sociales de la Salud” OR “Déterminants sociaux de la santé” OR “Determinante de Saúde” OR “Determinantes Estruturais da Saúde” OR “Determinantes Estruturais de Saúde” OR “Determinantes Sociais de Saúde” OR “Fatores Socioeconômicos” OR “Socioeconomic Factors” OR “Factores Socioeconómicos” OR “Facteurs socioéconomiques” OR “Aspectos Socioeconômicos” OR “Fatores Sociais” OR “Social Factors” OR “Factores Sociales” OR “Fatores Culturais” OR “Cultural Factors” OR “Factores Culturales” OR “Facteurs Culturels” OR “Fatores Econômicos” OR “Economic Factors” OR “Factores Económicos” OR “Facteurs économiques” OR “Fatores Raciais” OR “Race Factors” OR “Factores Raciales” OR “Facteurs raciaux” OR or “Condições Sociais” OR “Social Conditions” OR “Condiciones Sociales” OR “Conditions sociales” OR “Condição Social” OR “Condições de Vida” OR “Política de Saúde” OR “Health Policy” OR “Política de Salud” OR “Politique de santé” OR “Health Social Determinant” OR “Health Social Determinants” OR “Health Structural Determinant” OR “Health Structural Determinants” OR “Structural Determinants of Health” OR “Living Condition” OR “Living Conditions” OR “Social Condition”) AND (“Cuidado do Lactente” OR “Infant Care” OR “Cuidado del Lactante” OR “Soins du nourrisson” OR família OR family OR familia OR famille) AND ( db:(“LILACS” OR “IBECS” OR “BDENF” OR “INDEXPSI” OR “BINACIS” OR “BBO” OR “CUMED” OR “LIPECS” OR “MedCarib” OR “SES-SP” OR “colecionaSUS” OR “PAHOIRIS” OR “BIGG” OR “MULTIMEDIA” OR “CidSaude” OR “PAHO” OR “PIE” OR “PREPRINT-MEDRXIV” OR “PREPRINT-SCIELO” OR “VETINDEX”))
**MEDLINE/** PubMed	(“Infant, Premature” OR “Premature Birth” OR “Premature Infant” OR “Premature Infants”) AND (“Social Determinants of Health” OR “Socioeconomic Factors” OR “Social Factors” OR “Cultural Factors” OR “Economic Factors” OR “Race Factors” OR “Social Conditions” OR “Health Policy” OR “Health Social Determinant” OR “Health Social Determinants” OR “Health Structural Determinant” OR “Health Structural Determinants” OR “Structural Determinants of Health” OR “Living Condition” OR “Living Conditions” OR “Social Condition”) AND (“Infant Care” OR Family)
Scopus (Via Portal Capes ^†^ )	(“Infant, Premature” OR “Premature Birth” OR “Premature Infant” OR “Premature Infants”) AND (“Social Determinants of Health” OR “Socioeconomic Factors” OR “Social Factors” OR “Cultural Factors” OR “Economic Factors” OR “Race Factors” OR “Social Conditions” OR “Health Policy” OR “Health Social Determinant” OR “Health Social Determinants” OR “Health Structural Determinant” OR “Health Structural Determinants” OR “Structural Determinants of Health” OR “Living Condition” OR “Living Conditions” OR “Social Condition”) AND (“Infant Care” OR Family)
*Web of Science* (Via Portal CAPES ^†^ )	(“Infant, Premature” OR “Premature Birth” OR “Premature Infant” OR “Premature Infants”) AND (“Social Determinants of Health” OR “Socioeconomic Factors” OR “Social Factors” OR “Cultural Factors” OR “Economic Factors” OR “Race Factors” OR “Social Conditions” OR “Health Policy” OR “Health Social Determinant” OR “Health Social Determinants” OR “Health Structural Determinant” OR “Health Structural Determinants” OR “Structural Determinants of Health” OR “Living Condition” OR “Living Conditions” OR “Social Condition”) AND (“Infant Care” OR Family)
**EMBASE** ^‡^ (Via Portal CAPES ^†^ )	(prematurity) and (‘social determinants of health’ or socioeconomics or ‘social aspect’ or ‘cultural factor’ or ‘economic aspect’ or ‘social status’ or ‘health care policy’) and (‘infant care’ or family)

*BVS = Biblioteca Virtual em Saúde; ^†^CAPES = Coordenação de Aperfeiçoamento de Pessoal de Nível Superior; ^‡^EMBASE = *Excerpta Medica Database*

### Seleção das fontes de evidências e processo de extração dos dados

O processo de seleção dos artigos incluídos foi realizado por duas pesquisadoras independentes e apoiado pelo *softwareRayyan*. Inicialmente, foram excluídos os estudos duplicados, lidos os títulos e resumos dos demais artigos seguindo os critérios de elegibilidade. Os estudos restantes foram lidos na íntegra, seguindo os mesmos critérios. As divergências identificadas durante o processo de seleção foram analisadas por uma terceira pesquisadora.

### Análise dos dados

Os dados dos artigos foram extraídos por meio de um instrumento de codificação elaborado com base no modelo de DSS^(6)^, alinhado aos objetivos do artigo e ao PCC. Este instrumento foi estruturado no *software* MaxQDA e orientou a busca de informações para a coleta das variáveis de caracterização do estudo, bem como dos códigos e subcódigos dos DSS.

O código condições econômicas, sociais, culturais e ambientais diz respeito às condições econômicas, culturais e ambientais da sociedade^(4)^, e explora, principalmente, o contexto de cuidado em que a criança está inserida. Inclui os subcódigos: fatores socioeconômicos (disponibilidade ou não de recursos e oportunidades materiais); fatores culturais (valores, ideais, interesses e comportamentos de um determinado grupo social; inclui religião); fatores políticos e políticas públicas (políticas públicas que visam a redução das desigualdades sociais, que promovam mudanças de comportamento, acesso facilitado a alimentos saudáveis, programas que melhorem o acesso da população à saúde e à educação. Foram considerados também os programas de assistência social e institucionalização do cuidado^(4)^).

As condições de vida e trabalho elucidam, sobretudo, o contexto de cuidado a que a criança está exposta, e se referem à disponibilidade de alimentos e acesso aos ambientes e serviços essenciais, como saúde e educação^(4)^. Este código inclui os subcódigos: acesso à alimentação (disponibilidade e acesso à alimentação adequada); acesso aos serviços essenciais (serviços de educação e de saúde); educação (nível de escolaridade e instrução dos pais e familiares das crianças pré-termo); condições de habitação (condições de moradia, incluindo localização, estrutura e saneamento básico), e condições de trabalho (ocupação do familiar; se possui um trabalho remunerado ou está desempregado)^(4)^.

As redes sociais e de apoio da comunidade correspondem às redes comunitárias e de apoio, laços familiares e religiosos^(7)^. Os fatores comportamentais e de estilo de vida do indivíduo incluem os subcódigos: cuidado familiar (cuidado à criança ofertado pela família no âmbito do domicílio; cuidado canguru, contato pele a pele, que começa de forma precoce e crescente desde o toque evoluindo até a posição canguru^(21)^; apego, vínculo, laços duradouros estabelecidos entre os bebês e as mães ou cuidadores^(21)^); aleitamento materno (prática do aleitamento materno), e hábitos não saudáveis (hábitos de vida dos cuidadores que podem prejudicar a saúde infantil, como consumo de álcool e outras drogas, má alimentação e sedentarismo).

As características dos indivíduos compreendem as condições de saúde (circunstâncias na saúde do recém-nascido que exigem respostas por meio de assistência à saúde. Ex.: diagnósticos médicos atribuídos); idade, sexo e fatores hereditários (fatores associados à idade dos indivíduos, ao sexo e à hereditariedade); fatores psicológicos (abrange aspectos da saúde mental dos indivíduos presentes no contexto familiar que podem influenciar na saúde infantil); fatores étnicos e raciais (fatores relacionados a grupos étnicos e raciais). Por fim, os resultados em saúde das crianças foram identificados quando relacionados às condições de saúde, ao crescimento e ao desenvolvimento neuropsicomotor da criança decorrente da influência dos DSS.

A amostra final dos artigos foi importada para o *software* MaxQDA versão 2022, o qual suportou a codificação das informações. Este processo foi realizado de forma independente por duas autoras deste estudo, e os trechos codificados foram validados por uma terceira pesquisadora.

## Resultados

A Figura 2 apresenta o processo de seleção dos artigos. Foram inicialmente importados 3.218 estudos, sendo 160 excluídos por duplicidade. Após a leitura dos títulos e resumos, 2.991 foram excluídos, restando 67 estudos para a leitura na íntegra e a realização da busca reversa, sendo necessário ler mais 11 estudos. Um dos estudos não foi encontrado na íntegra, dessa forma, 77 estudos foram lidos na íntegra e destes, 31 compuseram a amostra final.

**Figura 2 -:** Fluxograma do processo de seleção dos artigos, segundo PRISMA-SCR^†^. Belo Horizonte, MG, Brasil, 2024

*n = Número; ^†^PRISMA-SCR = *Preferred Reporting Items for Systematic reviews and Meta-Analyses extension for Scoping Reviews*

Os 31 artigos incluídos neste estudo são apresentados na Figura 3, incluindo o título, tipo de estudo/país/ano de publicação, objetivo, dimensões avaliadas e amostra.

Dentre os artigos analisados, a maioria (80,65%) apresentou abordagem quantitativa. Quanto aos continentes de origem da publicação, 19 estudos foram realizados na América do Norte (A3, A4, A5, A6, A8, A9, A10, A11, A12, A13, A16, A17, A19, A20, A22, A23, A25, A27, A31), seis na Europa (A1, A2, A7, A18, A28, A29), quatro na América do Sul (A14, A15, A21, A24) e dois na Ásia (A26, A30). O ano com maior número de publicação foi 2021, com nove artigos, enquanto 2014 não registrou nenhuma publicação. Nos demais anos, a média de publicações foi de 2,38 artigos.

**Figura 3 -:** Caracterização dos estudos incluídos. Belo Horizonte, MG, Brasil, 2024

**Código** **do artigo**	**Título**	**Tipo de estudo, país, (ano de publicação)**	**Objetivo**	**Dimensões avaliadas**	**Amostra**
A01 ^( 22 )^	*Psychological distress and early lactation performance in mothers of late preterm infants*	Caso-controle, Itália, (2011).	Examinar a relação entre o sofrimento psicológico de mães que tiveram parto prematuro tardio e o desempenho no início da lactação.	Fatores comportamentais e de estilo de vida do indivíduo; características do indivíduo.	Grupo caso: 42 mães de RNPT* nascidos com IG ^†^ entre 34 e 36 semanas. Grupo controle: 42 mães de RN ^‡^ termo.
A02 ^( 23 )^	*Parenting stress in mothers after very preterm birth and the effect of the Infant* *Behavioural Assessment and Intervention Program*	Caso-controle, Holanda, (2011).	Examinar o estresse parental materno como um resultado secundário do *Infant Behavioral Assessment and Intervention Program* (IBAIP) ^§^ .	Condições econômicas, sociais, culturais e ambientais; condições de vida e trabalho; redes sociais e de apoio da comunidade; Fatores comportamentais e de estilo de vida do indivíduo; características do indivíduo.	Grupo intervenção: 86 RNPT* (nascidos com < 32 emanas de IG ^†^ e/ou peso ao nascer <1500 gramas) e 72 pais. Grupo controle: 90 RNPT* (nascidos com < 32 semanas de IG ^†^ e/ou peso ao nascer < 1500 gramas) e 79 pais.
A03 ^( 24 )^	*Evaluation of the ability of neurobiological, neurodevelopmental and socio-economic variables to predict cognitive outcome in premature infants*	Longitudinal, Estados Unidos, (2011).	Reavaliar a eficácia da *Neurobiologic Risk Score* (NBRS) ^||^ e N *eurodevelopmental Risk Exam* (NRE) ^¶^ e avaliar o papel preditivo relativo de fatores de risco socioeconômicos, isolados e combinados com fatores de risco biológicos e de desenvolvimento conhecidos.	Condições de vida e trabalho; redes sociais e de apoio da comunidade; características do indivíduo.	129 RNPT* (nascidos com < 32 semanas de IG ^†^ ).
A04 ^( 25 )^	*Effect of ethnicity and race on cognitive and language testing at 18 – 22 months in extremely preterm infants*	Coorte retrospectiva, Estados Unidos, (2012).	Avaliar a relação de raça/etnia com os escores cognitivos e de linguagem na *Bayley Scales of Infant and Toddler Development, Third Edition* (BSID-III)** em bebês prematuros extremos (<28 semanas de IG ^†^ ).	Condições de vida e trabalho; características do indivíduo.	865 RN ^‡^ extremamente prematuros (nascidos com < 28 semanas de IG ^†^ ).
A05 ^( 26 )^	*Parenting stress, social support, and mother-child interactions in families of multiple and singleton preterm toddlers*	Longitudinal, Estados Unidos, (2012).	Investigar o apoio familiar como um potencial moderador do estresse parental materno em famílias com filhos prematuros múltiplos e únicos.	Condições econômicas, sociais, culturais e ambientais; condições de vida e trabalho; redes sociais e de apoio da comunidade; fatores comportamentais e de estilo de vida do indivíduo; características do indivíduo.	153 famílias e RNPT* (nascidos com < 37 semanas de IG ^†^ ).
A06 ^( 27 )^	*Impact of maternal education on cognitive and language scores at 18 to 24 months among extremely preterms neonates*	Coorte, Canadá, (2013).	Explorar a associação entre os níveis de escolaridade materna e os escores compostos cognitivos e de linguagem da Escala Bayley III, aos 18 a 24 meses de idade corrigida em bebês prematuros extremos nascidos com < 29 semanas de IG ^†^ .	Condições de vida e trabalho; redes sociais e de apoio da comunidade; características do indivíduo.	457 famílias e RN ^‡^ extremamente prematuros (nascidos com < 29 semanas de IG ^†^ ).
A07 ^( 28 )^	*One-year neurodevelopmental outcome of very and late preterm infants: risk factors and correlation with maternal stress*	Longitudinal, Itália, (2015).	Descrever o perfil de desenvolvimento de lactentes pré-termo tardio, comparar o desenvolvimento cognitivo, linguístico e motor em lactentes pré-termo tardio e muito pré-termo aos 12 meses de idade corrigida; explorar a relação entre medidas de resultados de desenvolvimento e estresse materno nos dois subgrupos; analisar a influência dos fatores de risco neonatais e das características sociodemográficas maternas nas medidas dos resultados do desenvolvimento.	Condições de vida e trabalho; características do indivíduo.	Mães e 79 RNPT* - 39 prematuros tardios (nascidos entre 33-36 semanas de IG ^†^ ) - 40 muito prematuros (nascidos com ≤ 32 semanas de IG ^†^ ).
A08 ^( 29 )^	*Neurodevelopmental profile, growth, and psychosocial environment of preterm infants with difficult feeding behavior at age 2 years*	Coorte, Estados Unidos, (2015).	Examinar a associação de comportamentos alimentares difíceis em bebês muito prematuros aos dois anos de idade com resultados de crescimento e neurodesenvolvimento e fatores familiares e funcionamento.	Condições de vida e trabalho; redes sociais e de apoio da comunidade; características do indivíduo.	104 RNPT* (nascidos com ≤ 30 semanas de IG ^†^ ).
A09 ^( 30 )^	*Developmental outcomes of extremely preterm infants born to adolescente mothers*	Coorte retrospectiva, Estados Unidos, (2015).	Avaliar os resultados cognitivos, de linguagem e comportamento de bebês extremamente prematuros nascidos de mães adolescentes (< 20 anos) em comparação com bebês extremamente prematuros nascidos de mães adultas (≥ 20 anos); identificar os fatores de risco socioeconômicos que afetam os resultados.	Condições econômicas, sociais, culturais e ambientais; condições de vida e trabalho; redes sociais e de apoio da comunidade; fatores comportamentais e de estilo de vida do indivíduo; características do indivíduo.	1.934 RNPT* (nascidos com 27 semanas) - 211 RNPT* de mães adolescentes - 1.723 RNPT* de mães adultas.
A10 ^( 31 )^	*Health care use outcomes of an integrated hospital-to-home mother-preterm infant intervention*	Estudo controlado randomizado, Estados Unidos, (2016).	Comparar os cuidados de saúde desde a alta hospitalar até seis semanas de idade corrigida em dois grupos de díades mãe-bebê prematuro: aqueles que receberam uma intervenção, *Hospital to Home: Optimizing Premature Infant’s Environment* (H-HOPE) ^††^ e um grupo de controle de atenção.	Condições econômicas, sociais, culturais e ambientais; condições de vida e trabalho; redes sociais e de apoio da comunidade; características do indivíduo.	147 mães e RNPT* (nascidos entre 29 e 34 semanas de IG ^†^ ).
A11 ^( 32 )^	*Life after discharge: what parents of preterm infants say about their transition to home*	Descritivo e exploratório, Estados Unidos, (2016).	Explorar e descrever as experiências dos pais de bebês prematuros após a alta hospitalar.	Condições econômicas, sociais, culturais e ambientais; condições de vida e trabalho; redes sociais e de apoio da comunidade; fatores comportamentais e de estilo de vida do indivíduo; características do indivíduo.	52 pais de RNPT* (nascidos < 37 semanas).
A12 ^( 33 )^	*A qualitative study: mothers of late preterm infants relate their experiences of community-based care*	Descritivo de abordagem fenomenológica, Canadá, (2017).	Explorar a experiência das mães de cuidar de seus bebês prematuros tardios na comunidade.	Condições econômicas, sociais, culturais e ambientais; condições de vida e trabalho; redes sociais e de apoio da comunidade; fatores comportamentais e de estilo de vida do indivíduo; características do indivíduo.	11 mães de RNPT* tardios (nascidos entre 34 e 37 semanas de IG ^†^ ).
A13 ^( 34 )^	*Homecare and healthcare utilization errors post–neonatal intensive care unit discharge*	Coorte prospectiva, Estados Unidos, (2017).	Identificar e descrever os erros de cuidados domiciliares e de utilização de cuidados de saúde em bebês de alto risco após a alta da UTIN ^‡‡^ .	Condições econômicas, sociais, culturais e ambientais; condições de vida e trabalho; redes sociais e de apoio da comunidade; fatores comportamentais e de estilo de vida do indivíduo; características do indivíduo.	241 RNPT* - 163 nascidos com <32 semanas de IG ^†^ ou 1.500 gramas - 78 nascidos com > 32 semanas de IG ^†^ e mais de 1500 gramas
A14 ^( 35 )^	*Situación socioeconómica familiar y neurodesarrollo de prematuros de muy bajo peso al nacer a los dos años de edad*	Coorte retrospectiva, Chile, (2018).	Caracterizar as famílias onde crescem os prematuros de muito baixo peso ao nascer com nível socioeconômico baixo e médio baixo; explorar a possível associação entre as características sociofamiliares e o neurodesenvolvimento aos dois anos.	Condições econômicas, sociais, culturais e ambientais; condições de vida e trabalho; redes sociais e de apoio da comunidade; fatores comportamentais e de estilo de vida do indivíduo; características do indivíduo.	162 famílias de RNPT* de muito baixo peso ao nascer (nascidos < 32 semanas de IG ^†^ ou < 1500 gramas).
A15 ^( 36 )^	*Intervención basada en rutinas como apoyo a la participación familiar para el desarrollo del lenguaje en bebés prematuros*	Estudo de caso, Equador, (2020).	Apresentar o impacto que uma Intervenção Baseada em Rotinas (IBR) ^§§^ pode ter na participação da família para o desenvolvimento da linguagem de seus bebês prematuros.	Condições econômicas, sociais, culturais e ambientais; condições de vida e trabalho; redes sociais e de apoio da comunidade; fatores comportamentais e de estilo de vida do indivíduo; características do indivíduo.	11 famílias e 11 RNPT* (nascidos com < 37 semanas de IG ^†^ ).
A16 ^( 37 )^	*Disparities in preterm infant emergency room utilization and rehospitalization by maternal immigrant status*	Exploratório, Estados Unidos, (2020).	Avaliar os efeitos da condição de mãe imigrante e dos fatores de risco nas taxas de visitas ao pronto-socorro e reinternações de bebês prematuros até 90 dias após a alta.	Condições econômicas, sociais, culturais e ambientais; condições de vida e trabalho; redes sociais e de apoio da comunidade; fatores comportamentais e de estilo de vida do indivíduo; características do indivíduo.	732 mães e 866 RNPT* (nascidos com < 37 semanas de IG ^†^ ). - 176 mães imigrantes e 203 RNPT* - 556 mães nativas e 663 RNPT*.
A17 ^( 38 )^	*Home visiting for Neonatal Intensive Care Unit (NICU)* ^||||^ *graduates: impacts of Following Baby Back Home*	Coorte, Estados Unidos, (2021).	Examinar se a intervenção *Following Baby Back Home* (FBBH) ^¶¶^ , em comparação ao grupo controle, demonstraram aumento de cuidados de saúde e imunizações e menor mortalidade infantil desde a alta da UTIN ^‡‡^ até o primeiro ano de vida.	Condições econômicas, sociais, culturais e ambientais; condições de vida e trabalho; redes sociais e de apoio da comunidade; fatores comportamentais e de estilo de vida do indivíduo; características do indivíduo.	498 RNPT* baixo peso ao nascer (< 2500 gramas e < 37 semanas de IG ^†^ ).
A18 ^( 39 )^	*The effects of prematurity and socioeconomic deprivation on early speech perception: a story of two different delays*	Longitudinal, Reino Unido, (2020).	Compreender a interação entre a maturação física/cognitiva e a exposição ambiental à linguagem no desenvolvimento inicial da linguagem.	Condições econômicas, sociais, culturais e ambientais; condições de vida e trabalho.	38 RNPT* (nascidos ≤ 33 semanas de IG ^†^ ).
A19 ^( 40 )^	*Maternal post-traumatic stress and depression symptoms and outcomes after NICU* ^||||^ *discharge in a low-income sample: a cross-sectional study*	Transversal, Estados Unidos, (2021).	Descrever a prevalência de exames positivos para estresse pós-traumático (PTS)*** agudo e sintomas de depressão entre as famílias de baixa renda após a alta da UTIN ^‡‡^ . Examinar a associação ajustada de PTS*** e sintomas de depressão e resultados em saúde e neurodesenvolvimento infantil. Avaliar a associação ajustada de PTS*** e sintomas de depressão e qualidade de vida materna.	Condições econômicas, sociais, culturais e ambientais; condições de vida e trabalho; características do indivíduo.	150 famílias de RNPT* (nascidos < 37 semanas de IG ^†^ ).
A20 ^( 41 )^	*The association of care transitions measure-15 score and outcomes after discharge from the NICU* ^||||^	Transversal, Estados Unidos, (2021).	Descrever os escores do *Care Transitions Measure* (CTM) ^†††^ entre os cuidadores de prematuros após alta da UTIN ^‡‡^ . Descrever a associação de pontuações do CTM ^†††^ com readmissões, matrículas em programas de assistência pública e pontuações de qualidade de vida do cuidador.	Condições econômicas, sociais, culturais e ambientais; condições de vida e trabalho; características do indivíduo.	169 pais de RNPT* (nascidos com <37 semanas de IG ^†^ ).
A21 ^( 42 )^	*Factors associated with the socioemotional development of preterm infants*	Observacional longitudinal, Brasil, (2021).	Comparar a diferença no desenvolvimento socioemocional entre a alta hospitalar e aos seis meses de idade corrigida de lactentes nascidos ≤ 32 semanas e avaliar o impacto de fatores maternos e infantis na taxa de desenvolvimento socioemocional em um período de seis meses.	Condições econômicas, sociais, culturais e ambientais; condições de vida e trabalho; redes sociais e de apoio da comunidade; fatores comportamentais e de estilo de vida do indivíduo; características do indivíduo.	72 mães e RNPT* (nascidos com ≤ 32 semanas de IG ^†^ ).
A22 ^( 11 )^	*Maternal depressive symptoms, poverty, and young motherhood increase the odds of early depressive and anxiety disorders for children born prematurely*	Observacional longitudinal, Estados Unidos, (2021).	Identificar a prevalência de transtornos depressivos e de ansiedade aos dois anos de idade entre crianças nascidas prematuras e determinar até que ponto a pobreza, os sintomas depressivos maternos ou a maternidade jovem aumentam a probabilidade desses transtornos	Condições econômicas, sociais, culturais e ambientais; condições de vida e trabalho; redes sociais e de apoio da comunidade; fatores comportamentais e de estilo de vida do indivíduo; características do indivíduo.	105 mães e RNPT* (nascidos com < 37 semanas de IG ^†^ ).
A23 ^( 43 )^	*Risk factors for re-hospitalization following neonatal discharge of extremely preterm infants in Canada*	Coorte prospectiva, Canadá, (2021).	Identificar as características neonatais, sociodemográficas e geográficas que predizem a reinternação em recém-nascidos prematuros extremos canadenses.	Condições econômicas, sociais, culturais e ambientais; condições de vida e trabalho; características do indivíduo.	2.275 RNPT* (nascidos entre 22 e 28 semanas de IG ^†^ ).
A24 ^( 44 )^	*Risk factors for fine and gross motor development in preterm and term infants*	Observacional, Brasil, (2021).	Investigar a associação de fatores sociodemográficos, obstétricos e psicossociais com atraso no desenvolvimento motor fino e grosso em lactentes prematuros e a termo, na faixa etária de três meses e um dia a 12 meses e 29 dias.	Condições econômicas, sociais, culturais e ambientais; condições de vida e trabalho; redes sociais e de apoio da comunidade; fatores comportamentais e de estilo de vida do indivíduo; características do indivíduo.	RN ^‡^ a termo e pré-termo (< 37 semanas). Etapa 1 (3 – quatro meses e 29 dias): 103 termo e 62 RNPT* Etapa 2 (8 – nove meses e 29 dias): 84 termo e 46 RNPT* Etapa 3 (11- 12 meses e 29 dias): 69 termo e 33 RNPT*.
A25 ^( 45 )^	*Parent-reported health status of preterm survivors in a Canadian cohort*	Coorte prospectiva, Canadá, (2022).	Descrever o estado de saúde relatado pelos pais em uma grande coorte nacional de crianças nascidas prematuras extremas em idade pré-escolar e identificar as variáveis clínicas e sociodemográficas associadas ao estado de saúde.	Redes sociais e de apoio da comunidade; fatores comportamentais e de estilo de vida do indivíduo; características do indivíduo.	811 RNPT* (nascidos < 29 semanas de IG ^†^ ).
A26 ^( 14 )^	*Neurodevelopmental outcomes at 6, 12, and 24 months of age in preterm infants with very low birth weights in Taiwan*	Coorte, Taiwan, (2021).	Identificar os antecedentes perinatais associados à deficiência de neurodesenvolvimento para bebês prematuros de muito baixo peso aos 6, 12 e 24 meses de idade e a estabilidade das avaliações do neurodesenvolvimento.	Condições econômicas, sociais, culturais e ambientais; condições de vida e trabalho; redes sociais e de apoio da comunidade; características do indivíduo.	8.517 RNPT* de muito baixo peso (entre 24 e 32 semanas de IG ^†^ ).
A27 ^( 46 )^	*Association of inotrope use with neurodevelopmental outcomes in infants <29 weeks gestation: a retrospective cohort study*	Coorte retrospectiva, Canadá, (2021).	Comparar os resultados do neurodesenvolvimento aos 18-24 meses em bebês prematuros <29 semanas de idade gestacional que receberam *versus* aqueles que não receberam inotrópicos na primeira semana de vida.	Condições de vida e trabalho; fatores comportamentais e de estilo de vida do indivíduo; características do indivíduo.	2.615 RNPT* (<29 semanas de IG ^†^ ).
A28 ^( 47 )^	*Association of preterm birth and socioeconomic status with neonatal brain structure*	Coorte, Reino Unido, (2023).	Investigar associações entre a idade gestacional ao nascimento e o *status* socioeconômico com a morfologia cerebral neonatal.	Condições econômicas, sociais, culturais e ambientais; condições de vida e trabalho; fatores comportamentais e de estilo de vida do indivíduo; características do indivíduo.	170 RNPT* (< 33 semanas de IG ^†^ ) e 91 RN ^‡^ termo.
A29 ^( 48 )^	*Cortisol reactivity and negative affect among preterm infants at 12 months during a mother-infant interaction task*	Longitudinal, Portugal, (2023).	Investigar as correlações da reatividade ao cortisol do bebê prematuro e a associação com o afeto negativo do bebê.	Condições econômicas, sociais, culturais e ambientais; condições de vida e trabalho; fatores comportamentais e de estilo de vida do indivíduo; características do indivíduo.	48 RNPT*
A30 ^( 49 )^	*Early developmental support for preterm infants based on exploratory behaviors:a parallel randomized controlled study*	Estudo controlado randomizado, Turquia, (2023).	Investigar a eficiência do novo programa de intervenção precoce *Explorer Baby* nas habilidades motoras, cognitivas e de linguagem de bebês prematuros.	Condições de vida e trabalho; fatores comportamentais e de estilo de vida do indivíduo; características do indivíduo.	57 RNPT* (< 33 semanas de IG ^†^ ).
A31 ^( 50 )^	*Language outcomes at 18–24 months of preterm infants from US Spanish- and English-speaking homes*	Coorte retrospectiva, Estados Unidos, (2023).	Avaliar a associação entre o atraso de linguagem expressiva e receptiva leve a moderado e o idioma principal (espanhol e inglês) falado em casa de crianças prematuras usando o BSID-III** e examinar a associação com os fatores socioeconômicos.	Condições econômicas, sociais, culturais e ambientais; condições de vida e trabalho; redes sociais e de apoio da comunidade; fatores comportamentais e de estilo de vida do indivíduo; características do indivíduo.	1.125 RNPT* (< 32 semanas de IG ^†^ ).

*RNPT = Recém-Nascido Pré-Termo; ^†^IG = Idade Gestacional; ^‡^RN = Recém-Nascido; ^§^IBAIP = *Infant Behavioral Assessment and Intervention Program*; ^||^NBRS = *Neurobiologic Risk Score*; ^¶^NRE = N*eurodevelopmental Risk Exam*; **BSID-III = *Bayley Scales of Infant and Toddler Development, Third Edition*; ^††^H-HOPE = *Hospital to Home: Optimizing Premature Infant’s Environment*; ^‡‡^UTIN = Unidade de Terapia Intensiva Neonatal; ^§§^IBR = Intervenção Baseada em Rotinas; ^||||^NICU = *NeonatalIntensive Care Unit*; ^¶¶^FBBH = *Following Baby Back Home*; ***PTS = Estresse Pós-Traumático; ^†††^CTM = *Care Transitions Measure*

O processo de codificação dos estudos permitiu identificar as variáveis congruentes às dimensões dos DSS (Tabela 1) de acordo com os códigos e subcódigos descritos na seção análise dos dados.

**Tabela 1 -:** Variáveis por subcódigos e quantificadas para os estudos. Belo Horizonte, MG, Brasil, 2024

**Subcódigo**	n*	**%** ^†^ (n*/total)
**Condições econômicas, sociais, culturais e ambientais**	**22**	**70,97%** ^†^
Fatores socioeconômicos	18	58,06% ^†^
Fatores culturais	15	48,39% ^†^
Fatores ambientais	0	0,00% ^†^
Fatores políticos e políticas públicas	7	22,58% ^†^
**Condições de vida e trabalho**	**29**	**93,55%** ^†^
Acesso a alimentação	0	0,00% ^†^
Acesso a serviços essenciais	14	45,16% ^†^
Educação	27	87,10% ^†^
Condição de habitação	10	32,26% ^†^
Condição de trabalho	10	32,26% ^†^
**Redes sociais e de apoio da comunidade**	**21**	**67,74%** ^†^
Redes de apoio	21	67,74% ^†^
**Fatores comportamentais e de estilo de vida do indivíduo**	**19**	61,29% ^†^
Cuidado familiar	12	38,71% ^†^
Aleitamento materno	7	22,58% ^†^
Hábitos não saudáveis	8	25,81% ^†^
**Características do indivíduo**	**31**	**100%** ^†^
Condição de saúde	31	100% ^†^
Idade, sexo e fatores hereditários	31	100% ^†^
Fatores psicológicos	15	48,39% ^†^
Fatores étnicos e raciais	16	51,61% ^†^
**Resultados em saúde infantil**	**31**	**100%** ^†^
Desenvolvimento	19	61,29% ^†^
Comportamental	5	16,13% ^†^
Crescimento	4	12,90% ^†^
Afecções	5	16,13% ^†^
Mortalidade infantil	1	3,23% ^†^
Demandas por serviços de saúde	9	29,03% ^†^

*n = Número; ^†^ % = Porcentagem de estudos

Os artigos contemplaram todas as macrodimensões dos DSS condições de vida e trabalho, fatores comportamentais e de estilo de vida e características do indivíduo relativo às crianças nascidas pré-termo nos primeiros dois anos de vida, sendo este último explorado por todos os artigos.

As variáveis investigadas nos artigos analisados se encontram representadas especificamente de acordo com as macro e micro dimensões dos DSS na Tabela 2. As variáveis investigadas nos artigos analisados se encontram representadas especificamente de acordo com as macro e micro dimensões dos DSS na Tabela 2.

**Tabela 2 –:** Variáveis abordadas nos estudos e resultados em saúde das crianças relacionados aos códigos e subcódigos. Belo Horizonte, MG, Brasil, 2024

**Subcódigo**	**Artigos**	Qtd* (n ^†^ **)**	**%** ^‡^ **(n** ^†^ **/total)**
**Fatores socioeconômicos**			
Pobreza	A05, A15, A16, A22, A23	5	16,13% ^‡^
Renda familiar	A05, A10, A11, A12, A15, A17, A18, A19, A20, A22, A24, A28, A29	13	41,94% ^‡^
Condições socioeconômicas	A14, A18, A21, A26, A28	5	16,13% ^‡^
Bens de consumo duráveis	A05	1	3,23% ^‡^
Índice de risco sociodemográfico	A05	1	3,23% ^‡^
**Fatores culturais**			
Nacionalidade	A11, A12, A16, A22, A23, A24	6	19,35% ^‡^
Língua materna	A02, A09, A10, A13, A16, A18, A19, A20, A31	9	29,03% ^‡^
Qualidade de vida	A19, A20, A28	3	9,68% ^‡^
**Fatores políticos e políticas p** úblicas			
Programa de cuidado de transição	A11	1	3,23% ^‡^
Programa de suplementação alimentar	A17, A20	2	6,45% ^‡^
Benefícios sociais	A14, A20, A22	3	9,68% ^‡^
Serviço de proteção à criança	A16	1	3,23% ^‡^
Abuso doméstico	A16	1	3,23% ^‡^
Supervisão do Estado	A09	1	3,23% ^‡^
**Acesso aos serviços essenciais**			
Acesso aos serviços de saúde (pré-natal, maternidade, intervenção, especialização)	A09, A11, A16, A20, A24, A31	6	19,35% ^‡^
Acesso à intervenção de saúde	A02, A10, A13, A17, A20, A30, A31	7	22,58% ^†^
Assistência social	A23	1	3,23% ^‡^
Seguro saúde (público ou privado)	A09, A11, A16, A17, A31	5	16,13% ^‡^
Acesso aos atendimentos especializados ou seguimento ambulatorial	A09, A10, A03, A12, A31	5	16,13% ^‡^
Acompanhamento pós-alta na UTIN ^§^	A13	1	3,23% ^‡^
**Educação**			
Nível de escolaridade dos cuidadores	A02, A03, A04, A05, A06, A07, A08, A09, A10, A11, A12, A13, A14, A16, A17, A18, A19, A20, A22, A23, A24, A26, A27, A28, A29, A30, A31	27	87,10% ^‡^
**Condição de habitação**			
Local de residência/habitação	A09, A10, A14, A17, A23, A28	6	19,35% ^‡^
Número de habitantes/crianças na residência/superlotação	A09, A10, A14, A16, A23, A24, A29	7	22,58% ^†^
Distância entre o local de residência e o serviço em saúde	A23	1	3,23% ^‡^
Lar adotivo	A08	1	3,23% ^‡^
**Condições de trabalho**			
Situação de emprego	A08, A10, A14, A15, A23, A24, A25, A27, A28, A29	10	32,26% ^‡^
Ocupação do provedor	A08	1	3,23% ^‡^
**Redes de apoio**			
Situação marital do cuidador	A02, A03, A05, A06, A08, A09, A11, A12, A13, A14, A16, A17, A21, A24, A25, A26, A27, A31	18	58,06% ^‡^
Composição/funcionamento familiar	A09, A22	2	6,45% ^‡^
Apoio de familiares/amigos	A05, A09, A12, A15	4	12,90% ^‡^
Tipo de cuidador (remunerado/babá ou primário, creche)	A09, A11, A14, A21	4	12,90% ^‡^
Suporte social	A10, A24	2	6,45% ^‡^
**Cuidado familiar**			
Organização/rotina de cuidado	A11, A15, A24, A30	4	12,90% ^‡^
Suporte materno para a resolução de problemas pela criança	A05	1	3,23% ^‡^
Cuidados inadequados relacionados à consulta, administração de medicamentos, alimentação, uso de equipamentos	A13	1	3,23% ^‡^
Cuidado familiar e profissional (ex: creche, cuidado em casa, cuidadora profissional ou babá)	A09, A31	2	6,45% ^‡^
Cuidado Canguru	A21, A30	2	6,45% ^‡^
Apego (interação materno-infantil)	A02, A05, A21, A22, A29, A30	6	19,35% ^‡^
**Aleitamento materno**			
Situação do aleitamento	A01, A16, A17, A21, A28, A31	6	19,35% ^‡^
Desafios na amamentação (ex: problemas de lactação, pega, outros)	A11	1	3,23% ^‡^
Aleitamento materno à alta hospitalar	A16	1	3,23% ^‡^
**Hábitos não saudáveis**			
Consumo de álcool	A12, A14	2	6,45% ^‡^
Uso de substâncias/medicações durante a gestação	A16, A24, A25	3	9,68% ^‡^
Tabagismo	A01, A17, A28	3	9,68% ^‡^
**Condição de saúde**			
Peso ao nascer	A01, A02, A03, A04, A05, A06, A07, A08, A09, A10, A11, A12, A13, A14, A16, A17, A18, A19, A20, A21, A23, A24, A26, A27, A28, A29, A30, A31	28	90,32% ^‡^
Medidas antropométricas (peso, estatura, perímetro cefálico)	A08, A14, A28, A31	4	12,90% ^‡^
Alterações de crescimento	A19, A20, A26	3	9,68% ^‡^
Escore de Apgar	A01, A05, A06, A10, A12, A17, A26, A27, A29, A31	10	32,26% ^‡^
Via de parto	A01, A06, A07, A12, A24	5	16,13% ^‡^
Grau de prematuridade	A16, A31	2	6,45% ^‡^
Transferência neonatal após o nascimento	A26	1	3,23% ^‡^
Icterícia	A12	1	3,23% ^‡^
Gemelaridade	A02, A04, A05, A06, A07, A08, A10, A11, A15, A16, A23, A28, A29	13	41,94% ^‡^
Uso de dispositivos (traque/gastrostomia, tutor para locomoção, uso de oxigênio e ventilação mecânica, CPAP ^||^ )	A03, A05, A14, A17, A19, A20, A23, A24, A26, A29	10	32,26% ^‡^
Refluxo gastroesofágico	A29	1	3,23% ^‡^
Ordem de nascimento	A02, A05	2	6,45% ^‡^
Reanimação ao nascimento	A26	1	3,23% ^‡^
Uso de oxigênio	A02, A14, A16, A19, A20, A23, A29, A31	8	25,81% ^‡^
Sepse	A02, A04, A07, A09, A12, A14, A16, A26, A27	9	29,03% ^‡^
Lesão neurológica (ex.: hemorragia intracerebral e/ou intraventricular e/ou periventricular; leucomalacia periventricular; paralisia cerebral)	A02, A03, A04, A06, A07, A08, A09, A14, A16, A17, A19, A20, A21, A23, A25, A26, A27, A31	18	58,06% ^‡^
Dias de vida à alta	A13	1	3,23% ^‡^
Comprometimento circulatório	A26	1	3,23% ^‡^
Tempo de ventilação mecânica	A09	1	3,23% ^‡^
pH sanguíneo	A03	1	3,23% ^‡^
Convulsões	A03, A14, A17, A26, A31	5	16,13% ^‡^
Hipotermia	A12,	1	3,23% ^‡^
Hipoglicemia	A03, A12	2	6,45% ^‡^
Comprometimento do neurodesenvolvimento	A03, A25	2	6,45% ^‡^
Avaliação neurológica	A03, A31	2	6,45% ^‡^
Cegueira	A04, A31	2	6,45% ^‡^
Função motora	A04, A30	2	6,45% ^‡^
Tempo de internação	A05, A07, A09, A13, A15, A16, A21, A23, A24, A26, A27, A29, A30, A31	14	45,16% ^‡^
Alterações respiratórias (apneia, doença respiratória, problema respiratório, síndrome do desconforto respiratório, doença pulmonar crônica, displasia broncopulmonar, deficiência de surfactante)	A04, A05, A06, A07, A09, A12, A16, A17, A19, A20, A23, A25, A26, A27, A29, A30, A31	17	54,84% ^‡^
Alterações cardiovasculares	A07, A12, A17	3	9,68% ^‡^
ROP ^¶^ /ROP ^¶^ III-V	A07, A09, A15, A19, A20, A25, A26, A27	8	25,81% ^‡^
Condição de saúde à alta hospitalar	A05, A06, A07, A25	4	12,90% ^‡^
Tipo de alimentação	A24	1	3,23% ^‡^
Dificuldades de alimentação	A08, A12, A24	3	9,68% ^‡^
Uso de esteroides pré/pós-natal	A01, A04, A06, A09, A26, A27	6	19,35% ^‡^
Hidrocefalia	A14, A26, A31	3	9,68% ^‡^
Anormalidade cromossômica	A17	1	3,23% ^‡^
Demanda por serviço de saúde (≥ 2 agendamentos/mês, atendimento de rotina ou adoecimento, readmissão hospitalar, cirurgias, tempo de reinternação, diagnóstico pós-alta)	A10, A11, A17, A19, A20	5	16,13% ^‡^
Alimentação por gavagem	A19, A20, A23	3	9,68% ^‡^
Nível de desenvolvimento cognitivo e motor	A22, A30, A31	1	3,23% ^‡^
Nível de habilidade de linguagem	A30, A31	2	6,45% ^‡^
Mortalidade	A27	1	3,23% ^‡^
Morbidade neonatal ou infantil	A10, A22, A26	3	9,68% ^‡^
Alterações infecciosas (infecção, pneumonia, enterocolite)	A03, A09, A14, A16, A19, A20, A23, A26, A27, A31	10	32,26% ^‡^
Perda auditiva/surdez	A04, A31	1	3,23% ^‡^
Doenças maternas e história obstétrica	A01	1	3,23% ^‡^
Doenças crônicas	A01	1	3,23% ^‡^
Ruptura prematura de membranas	A09, A26	2	6,45% ^‡^
Descolamento prematuro de placenta	A26	1	3,23% ^‡^
Paridade	A01, A09, A10, A12, A13, A16, A21, A24, A26, A31	10	32,26% ^‡^
Aborto	A24	1	3,23% ^‡^
Complicações pré e pós-parto	A24	1	3,23% ^‡^
Hipertensão	A06, A09, A26, A27, A31	5	16,13% ^‡^
Diabetes *Mellitus* /Diabetes *Mellitus* Gestacional	A26, A27	2	6,45% ^‡^
Pré-eclâmpsia	A09, A26	2	6,45% ^‡^
Corioamnionite	A06, A09, A26, A27, A31	5	16,13% ^‡^
Crescimento intrauterino restrito	A26	1	3,23% ^‡^
Via de parto	A09, A26, A24, A27, A31	5	16,13% ^‡^
Administração antenatal de corticosteroides	A09, A23, A26, A27	4	12,90% ^‡^
Gemelaridade	A09, A12, A26, A27	4	12,90% ^‡^
Hemorragia pré-parto	A26	1	3,23% ^‡^
Placenta prévia	A26	1	3,23% ^‡^
Administração de sulfato de magnésio pré-natal	A27	1	3,23% ^‡^
Tecnologia reprodutiva assistida	A27	1	3,23% ^‡^
Oligoidrâmnio e Polidrâmnio	A26	1	3,23% ^‡^
**Idade, sexo e fatores hereditários**			
Idade/idade gestacional	A01, A02, A03, A04, A05, A06, A07, A08 A09, A10, A11, A12, A13, A14, A15, A16, A17, A18, A19, A20, A21, A22, A23, A24, A25, A26, A27, A28, A29, A30, A31	31	100% ^‡^
Sexo	A02, A03, A04, A05, A06, A07, A08, A09, A10, A12, A13, A14, A15, A16, A17, A18, A21, A22, A23, A24, A25, A26, A27, A28, A29, A30, A31	27	87,10% ^‡^
**Fatores psicológicos**			
Depressão e ansiedade	A08, A22	2	6,45% ^‡^
Ansiedade/traço	A01, A08, A10, A12, A21, A29	5	16,13% ^‡^
Depressão/sintomas	A01, A05, A08, A10, A12, A19, A21, A22, A29	8	25,81% ^‡^
Estresse/estresse pós-traumático	A01, A02, A05, A07, A08, A11, A12, A19, A29, A30	10	32,26% ^‡^
Fadiga/exaustão	A11	1	3,23% ^‡^
Culpa	A11	1	3,23% ^‡^
Dificuldade em partilhar Sentimentos	A11	1	3,23% ^‡^
Isolamento social/solidão	A11	1	3,23% ^‡^
Diagnóstico de saúde mental	A16	1	3,23% ^‡^
Fatores psicológicos (história de depressão, gravidez planejada, sentimentos sobre a maternidade)	A24	1	3,23% ^‡^
Sofrimento psicológico	A02	1	3,23% ^‡^
**Fatores** é **tnicos e raciais**			
Raça	A04, A05, A09, A10, A13, A16, A17, A19, A20, A22, A31	11	35,49% ^‡^
Etnia	A02, A04, A05, A08, A09, A10, A12, A13, A16, A17, A19, A20, A22, A23, A28, A31	16	51,61% ^‡^
**Resultado em saúde das crianças**			
**Desenvolvimento**	-	**19**	-
Cognitivo	A03, A04, A06, A07, A08, A09, A19, A25, A27, A30, A31	11	-
Linguístico	A04, A06, A07, A08, A09, A18, A25, A30, A31	9	-
Motor	A07, A08, A09, A19, A24, A25, A27	7	-
Neurológico	A09, A14, A25	3	-
Socioemocional	A08, A09, A21, A22, A29	5	-
Geral	A15	1	-
Comunicação, socialização e habilidades de vida diária	A19, A30, A31	3	-
Desenvolvimento mental	A26	1	-
Desenvolvimento psicomotor	A26	1	-
Cerebral	A28	1	-
**Comportamental**		**5**	-
Comportamento infantil	A02, A05, A08, A09, A21	5	-
**Crescimento**		**4**	-
Perímetro cefálico	A09	1	-
Crescimento/peso	A09, A11, A12, A13	4	-
**Afecções**		**5**	-
Dificuldades alimentares	A08	1	-
Paralisia cerebral	A09	1	-
Deficiências visuais ou neurossensoriais/auditivas	A09, A25, A27	3	-
Estado de saúde (saudável ou não)	A01	1	-
**Mortalidade infantil**	A17	1	-
**Demandas por serviço de saúde** (consulta de rotina, imunização, reinternação, emergência, pronto-socorro ou cuidados intensivos)	A09, A10, A11, A13, A16, A17, A19, A20, A23	9	-

*Qtd = Quantidade; ^†^n = Número; ^‡^% = Porcentagem de estudos; ^§^UTIN = Unidade de Terapia Intensiva Neonatal; ^||^CPAP = *Continuous Positive Airway Pressure*; ^¶^ROP = Retinopatia da Prematuridade

Em relação à dimensão de *Características Individuais*, a totalidade dos estudos investigaram as “Condições de Saúde”, com ênfase para a variável peso ao nascer, que foi abordada em 28 artigos (90,32%). Todos os estudos também se utilizaram das variáveis para investigar a dimensão *Idade, Sexo e Fatores Hereditário*s. Nesta dimensão os aspectos menos investigados foram os “fatores psicológicos” em 15 estudos (48,39%) e os “Fatores Étnicos e Raciais” abordados em 16 estudos (51,61%).

Quanto à dimensão de *Condições de Vida e Trabalho*, 14 artigos (45,16%) abordaram os aspectos relacionados ao “acesso aos serviços essenciais”, enquanto 10 (32,26%) investigaram as “condições de habitação”. Também foram identificadas variáveis contemplando os *Fatores Comportamentais e de Estilo de Vida do Indivíduo* com sete (22,58%) artigos abordando o “aleitamento materno” e 12 (38,71%), o “cuidado familiar”. Não foram identificadas variáveis que permitissem explorar as dimensões dos DSS referente aos “fatores ambientais” e ao “acesso à alimentação”.

Os resultados em saúde da criança investigados (Tabela 2) estiveram em sua maioria (n=19) relacionados ao desenvolvimento, mensurado a partir de diferentes escalas, como a *Bayley Scales of Infant and Toddler Development, Third Edition* (BSID-III) (desenvolvimento linguístico, cognitivo e motor), *Cognitive Adaptive Test*/*Clinical Linguistic and Auditory Milestone Scale* (CAT/CLAMS) (desenvolvimento cognitivo), DENVER (desenvolvimento motor), *Gross Motor Function Classification System* (GMFCS) e *Bayley Scales of Infant Development, Second Edition* (BSID-II) (desenvolvimento neurológico), *Brief Infant-Toddler Social and Emotional Assessment* (BITSEA) e *Mother-baby Interaction Observation Protocol* (POIMB) (desenvolvimento socioemocional/comportamental), *Ages and Stages Questionnaires 3* (ASQ-3) (desenvolvimento geral). Do total de estudos, apenas quatro investigaram os resultados relacionados ao crescimento (peso, perímetro cefálico e crescimento).

## Discussão

A análise dos estudos permitiu evidenciar os aspectos que estão sendo abordados nas produções científicas que contribuem para o conhecimento sobre as crianças nascidas pré-termo nos primeiros dois anos de vida e que considerem as dimensões dos DSS^(6)^.

Embora os estudos abordem variáveis que contribuem para conhecer os DSS, nenhum emprega o referencial teórico^(6)^ para o seu desenvolvimento. A utilização deste referencial pode contribuir para a compreensão dos modos de viver e adoecer das crianças. Sabe-se que compreender o contexto em que a criança está inserida é fundamental para uma abordagem abrangente, possibilita uma visão ampliada e permite a formulação de cuidados individualizados mais eficazes e planejamento de políticas públicas.

Em relação à dimensão *Condições Econômicas, Sociais, Culturais e Ambientais*, identifica-se que a variável fator socioeconômico foi abordada em 18 artigos, buscando medir especialmente a renda familiar, pobreza e condições socioeconômicas. Um dos estudos analisados apontou a importância de elaborar estratégias para amenizar os efeitos adversos de níveis familiares socioeconômicos desfavoráveis, que impactavam diretamente no neurodesenvolvimento de crianças prematuras e seus resultados educacionais^(47)^. Outrossim, as condições sociais desfavoráveis como baixa renda familiar e pobreza, foram associadas aos sintomas de ansiedade e maiores sintomas de depressão, respectivamente^(11)^, influenciando no processo de adoecimento infantil.

Os fatores culturais foram predominantemente examinados a partir do idioma e da nacionalidade, evidenciando uma maior chance de reinternação e tempo de permanência hospitalar entre os filhos de mães imigrantes, se comparados aos de mães nativas^(37)^. Sabe-se que as pessoas que vivem como imigrantes podem ser expostas às situações de vulnerabilidade e inseguranças, e nesse contexto de cuidado suas crianças podem apresentar maior risco de saúde.

As questões relacionadas aos fatores políticos e políticas públicas foram identificadas como benefícios sociais, programas e serviços de proteção, dentre outros. Tais programas e benefícios sociais auxiliam no acesso às informações e afetam positivamente a saúde infantil, como demonstrado pelo programa *Following Baby Back Home*, que otimiza a busca pelos cuidados de saúde, diminuindo as taxas de mortalidade de crianças nascidas pré-termo com baixo peso ao nascer^(38)^. Por outro lado, a supervisão estatal, apontada como indicador de abuso e negligência predominante entre as mães adolescentes, sucedeu o aumento dos riscos de efeitos adversos cognitivos, de linguagem e motor^(30)^.

Embora os estudos desta revisão não tenham identificado os aspectos relacionados ao acesso à alimentação, as pesquisas mostraram que as famílias em situação de insegurança alimentar necessitam de no mínimo dois salários-mínimos para suprir suas despesas mensais^(51)^. Explorar as condições socioambientais às quais as crianças estão submetidas é um aspecto que precisa ser explorado em estudos posteriores, visto que esses determinantes podem modificar o crescimento e o desenvolvimento na prematuridade^(17)^. A vulnerabilidade relacionada às condições de saneamento e o acesso à alimentação aumenta os riscos de desnutrição, infecções gastrointestinais e, consequentemente, aumenta a necessidade de idas ao serviço de saúde^(52-53)^.

Ao seguir a dimensão *Condições de Vida e de Trabalho*, o acesso aos serviços essenciais foi investigado na perspectiva do seguro saúde, do acesso às intervenções e dos serviços, como maternidade e seguimento ambulatorial. Os serviços especiais, como o programa de visita domiciliar ou acesso ao serviço social, foram apontados como grandes aliados para a saúde das crianças nascidas pré-termo, uma vez que aumentam os cuidados de saúde, reduzindo o risco de mortalidade e amenizando os efeitos adversos para o desenvolvimento^(30,38)^. A limitação do acesso aos serviços de saúde pode estar relacionada às questões do próprio sistema, como escassez de profissionais e a organização inadequada, além de questões sociais, como a escolaridade e os fatores socioeconômicos^(54)^. Assim, infere-se que é necessário que desde o pré-natal os profissionais busquem identificar as barreiras que podem dificultar o acesso da família aos serviços, de forma a desenvolver estratégias que melhorem os desfechos perinatais.

Desse modo, sabe-se que crianças egressas da UTIN, especialmente as pré-termo, têm potencial risco de alteração no crescimento, desenvolvimento e necessidade aumentada de idas aos serviços de saúde acima do habitual devido às intercorrências^(12,31)^. Assim, é necessário que o acompanhamento pós-alta em serviços ambulatoriais seja bem estabelecido, com vistas a proporcionar uma avaliação multidisciplinar à criança e, consequentemente, minimizar os possíveis danos por meio da identificação precoce de alterações e orientações de cuidado^(12)^.

A educação evidenciou importantes desfechos, como o impacto do nível de escolaridade materna nos níveis de neurodesenvolvimento de crianças nascidas pré-termo, com destaque para o cognitivo e de linguagem^(27)^. Outro estudo da literatura identificou que níveis econômicos e disponibilidade de recursos no ambiente domiciliar, como brinquedos e atividades realizadas com os pais, melhoraram o desenvolvimento cognitivo entre 24 a 42 meses^(55)^. Estes resultados reforçam a importância de explorar o ambiente em que a criança está inserida.

Em relação a como as crianças vivem, destacam-se estudos que exploram a habitação^(43)^ e organização familiar para o cuidado^(44)^. Em relação às *Condições de Habitação*, os estudos desta revisão apontaram que o número de habitantes na residência aumentou as chances de reinternação^(43)^, o que pode estar relacionado à transmissão de doenças. Por outro lado, a presença de mais pessoas no domicílio e ter irmãos esteve associada a menor chance de atraso na motricidade fina e grossa, respectivamente^(44)^, uma vez que os demais membros da família estimulam o desenvolvimento da criança^(36)^. Entretanto, quando o aspecto é a condição de trabalho materna, a criança ser cuidada por uma mãe que exerce algum tipo de atividade profissional relacionou-se à maior chance do filho apresentar atraso na motricidade fina^(44)^.

Nesta revisão de escopo a dimensão *Redes sociais e de Apoio da Comunidade* contemplou 21 artigos e identificou componentes como: situação marital, composição familiar, apoio de familiares/amigos, cuidador remunerado, entre outros. Destaca-se a importância da rede de apoio, uma vez que ampara a mãe e, consequentemente, proporciona contextos favoráveis para o desenvolvimento infantil^(36)^. Ademais, figuras maternas familiares como avós são vistas como referências de conhecimento com base em suas vivências, e pode ser influenciada por suas crenças e valores^(56)^.

A dimensão *Fatores Comportamentais e de Estilo de Vida do Indivíduo* foi a menos explorada pelos estudos. Estes resultados podem ter sido influenciados pelo contexto domiciliar das investigações, entretanto, a literatura aponta que o apoio ao cuidado canguru por meio de visitas domiciliares esteve relacionado à redução na mortalidade neonatal e infantil entre os bebês com baixo peso ao nascer^(57)^. Além disso, as crianças nascidas pré-termo precoces apresentam riscos para o estabelecimento favorável do aleitamento materno^(22)^, aumentando as chances de desmame precoce, que podem ser contornados por meio de orientações quanto ao início e ao estabelecimento da amamentação^(57-58)^.

O cuidado familiar interfere no desenvolvimento e no crescimento adequado da criança, e pode ampliar ou limitar a promoção da saúde de acordo com os DSS presentes na realidade da família^(36)^. Portanto, considerar a variável cuidado familiar em investigações futuras poderá permitir uma melhor compreensão sobre os sentimentos vividos e os desafios enfrentados pelos cuidadores, evidenciando a necessidade de elaboração de estratégias de apoio às famílias^(59)^. O cuidado também pode ser investigado na perspectiva de um cuidador profissional em instituições educacionais como creches que, associadas ao cuidado familiar no domicílio, favorecem o desenvolvimento infantil^(55)^.

O apego diz respeito aos vínculos estabelecidos entre os cuidadores e as crianças, encontrados nesta revisão como interação materno-infantil. Alguns estudos revelaram que a interação foi influenciada pela saúde emocional materna em casos de estresse, ansiedade e depressão, levando a oferta de menos estímulo para a criança e, consequentemente, o aumento dos riscos para o desenvolvimento^(10,26)^. Diante destes impactos, torna-se indispensável uma maior investigação do apego, permitindo identificar os fatores de risco onde os profissionais podem criar estratégias que orientem e deem suporte às famílias, e os fatores de proteção que apoiem o crescimento e o desenvolvimento saudável infantil.

Em relação aos hábitos não saudáveis, nesta revisão ficou evidente que as crianças vivendo com alguém que consome álcool de forma abusiva tem cinco vezes mais chances de apresentar atraso no neurodesenvolvimento^(35)^. Ao se considerar que estes hábitos são fatores modificáveis, um estudo apontou a necessidade do desenvolvimento de políticas focadas na redução do consumo de álcool e tabaco, visto que estes têm sido associados ao desenvolvimento inadequado na primeira infância^(60)^.

Todos os estudos investigaram a dimensão *Características Individuais*, empregando as variáveis idade, sexo e fatores hereditários; condições de saúde frequentemente associada à história obstétrica, condição de nascimento, crescimento, desenvolvimento e necessidades por serviços de saúde. Os comprometimentos causados em razão destas variáveis têm potencial para aumentar a demanda de cuidados ao longo da vida da criança. Assim, a identificação das variáveis que estão sendo estudadas permite compreender o impacto de cada uma delas, como é o caso da associação entre o maior tempo de internação e o comprometimento do desenvolvimento socioemocional, cognitivo e motor^(42,61)^.

Outro ponto que se faz necessário discutir frente ao contexto da prematuridade é a abordagem dos fatores psicológicos, tratados em 15 artigos. A maternidade apresenta-se como uma fase de vulnerabilidade. Ao somar-se à prematuridade, contexto que impõe desafios à família e, principalmente, à mãe, pois, torna-se um fator de risco para casos de depressão^(10)^. Ademais, o suporte limitado dos profissionais a estas famílias após a alta resulta em fadiga e isolamento social, aumentando os custos de saúde frente às complicações evitáveis do cuidado falho ao prematuro^(32)^. No entanto, uma revisão de literatura revelou que nem sempre o sofrimento mental materno é identificado, e, pode causar desdobramentos na saúde integral da criança^(62)^. Diante disso, ressalta-se a necessidade de adotar o cuidado centrado no paciente e na família, a fim de se acompanhar os fatores com potencial influência, bem como estabelecer estratégias que promovam o bem-estar. Um estudo evidenciou que o cuidado centrado no paciente e na família promove impactos positivos, como o aumento da satisfação da família, os melhores resultados e qualidade de vida para as crianças^(63)^.

Os fatores étnicos e raciais expõem uma lacuna social frente às desigualdades sociais entrelaçadas a esta temática, trazendo consequências para o processo de desenvolvimento e crescimento infantil frente às condições socioeconômicas e raciais estruturais^(7,64)^. Alguns estudos revisados exemplificaram isso ao trazer a associação entre raça, mãe adolescentes e linguagem^(30)^, como por exemplo, crianças negras e hispânico-brancas possuem maior risco de atraso de linguagem em comparação às crianças brancas^(25)^.

Observa-se que os desfechos para a saúde da criança mais relacionados aos DSS e mais estudados têm sido o desenvolvimento, em especial cognitivo, linguístico e motor, seguido pelas demandas por serviços de saúde, relacionadas ou não às afecções. Estes resultados refletiram a relevância do acompanhamento da criança no contexto em que se encontra inserida, principalmente no que diz respeito à criticidade dos primeiros dois anos de vida em razão da neuroplasticidade, momento oportuno para as intervenções e estímulos ao desenvolvimento^(12)^. Dessa forma, é reforçada a necessidade de atuação preventiva sobre os fatores modificáveis que representam um risco para o crescimento e desenvolvimento pleno, comprometendo a saúde integral infantil.

Neste estudo entendeu-se que as variáveis contribuem para a identificação das dimensões dos DSS exploradas nas pesquisas científicas, contudo, reconhece-se sua limitação para representar estas dimensões em sua totalidade e isso foi considerado na discussão dos dados analisados. Ademais, o estudo apresenta limitações relacionadas à idade das crianças, uma vez que exclui os estudos que ampliaram a investigação para maiores de dois anos e restringe a compressão dos DSS a esta faixa etária. Outros contextos de cuidado para além do domiciliar e a inclusão de literatura cinzenta poderiam oferecer informações importantes sobre a saúde infantil.

## Conclusão

A identificação sobre a saúde da criança prematura por meio do referencial teórico dos DSS evidenciou os aspectos protetores e ameaçadores do ambiente no qual as crianças vivem. Os estudos analisados mostraram um maior interesse em explorar as variáveis sobre as características do indivíduo, com destaque para as condições de saúde e idade, sexo e fatores hereditários, realçando a busca por fatores de impacto direto na saúde infantil. Por outro lado, poucos estudos investigaram as variáveis das dimensões *Condições Econômicas, Sociais, Culturais e Ambientais, Redes Sociais e de Apoio da Comunidade* e *Fatores Comportamentais e de Estilo de Vida do Indivíduo*, indicando lacunas existentes na compreensão dos DSS na saúde infantil.

Destaca-se que os estudos investigaram as variáveis de forma isolada das dimensões dos DSS, dificultando a identificação de iniquidades e a análise da sua repercussão na saúde dos prematuros. É fundamental que os futuros estudos abordem os DSS de forma integrada, permitindo uma compreensão das condições de vida da população estudada e dos impactos do modo de viver e adoecer das crianças nascidas pré-termo nos primeiros dois anos de vida no crescimento, desenvolvimento e comportamento infantil. Essa abordagem ampliada facilitará a elaboração de cuidados centrados no indivíduo e permitirá intervenções precoces frente aos desfechos de saúde infantil.

# Translation: Determinación social en la salud de niños nacidos pretérmino: revisión de alcance ^*^

**DOI:** 10.1590/1518-8345.7720.4604

**Authors:** Mariana Fuentes Mendoza Rodrigues Soares, Amanda de Azevedo Gomes, Juliana Barony da Silva, Luciano Marques dos Santos, Patrícia Pinto Braga, Elysângela Dittz Duarte

**Affiliations:** 1 Universidade Federal de Minas Gerais, Escola de Enfermagem, Belo Horizonte, MG, Brasil.; 2 Universidade Estadual de Feira de Santana, Departamento de Saúde, Feira de Santana, BA, Brasil.; 3 Universidade Federal de São João del Rei, Curso de Enfermagem, Divinópolis, MG, Brasil.

**Keywords:** Determinantes Sociales de la Salud, Determinantes Estructurales de la Salud, Recien Nacido Prematuro, Salud Infantil, Crecimiento y Desarrollo, Cuidado del Niño

## Abstract

mapear el conocimiento científico sobre las dimensiones de los Determinantes Sociales de la Salud en la salud de niños nacidos pretérmino en los primeros dos años de vida.

se trata de una *scoping review* orientada por el *Joanna Briggs Institute* y por el *Preferred Reporting Items for Systematic Reviews and Meta-Analyses extension for Scoping Reviews*. De los estudios encontrados en cinco bases de datos y en la lista de referencias de aquellos seleccionados, se extrajeron los datos aplicando un instrumento de codificación elaborado a partir del modelo teórico de los Determinantes Sociales de la Salud con el apoyo del *software* MaxQDA.

se incluyeron 31 publicaciones. Las dimensiones de los determinantes sociales para la salud infantil más investigadas estuvieron relacionadas con las características del individuo, destacándose las condiciones de salud, edad, sexo y factores hereditarios, lo que resalta la búsqueda de factores con repercusión directa en la salud infantil.

las dimensiones de la salud infantil más exploradas fueron las relacionadas con el desarrollo del niño. El comportamiento y las condiciones de vida fueron poco explorados. Futuros estudios con un enfoque ampliado de los Determinantes Sociales de la Salud de forma integrada podrán contribuir a la elaboración de cuidados centrados en los prematuros y a intervenciones tempranas frente a los desenlaces de la salud infantil.

## Destacados

**(1)** Estudios destacan la influencia de las características individuales en la salud infantil. **(2)** Los determinantes sociales interfieren en el desarrollo y en la demanda de servicios de salud. **(3)** Se constatan las brechas en la investigación de los DSS y sus efectos en la salud infantil. **(4)** Se requiere abordar los Determinantes Sociales de la Salud de manera integrada.

## Introducción

Aunque los factores de riesgo para la prematuridad son ampliamente reconocidos^(1)^, las disparidades que contribuyen al parto prematuro y sus desenlaces en la salud infantil aún se comprenden poco. Un creciente cuerpo de evidencias destaca la importancia de abordar también los factores sociales y ambientales para prevenir el parto prematuro y mejorar la calidad de vida de las familias que tienen niños nacidos pretérmino^(2-3)^. Además de las disparidades inherentes al nacimiento pretérmino, las circunstancias en que se encuentra el individuo, como sus condiciones de vida y trabajo, repercuten directamente en su situación de salud^(4)^, y se definen como Determinantes Sociales de la Salud (DSS)^(5)^. Dichos factores fueron esquematizados en capas, destacando los niveles de alcance de los DSS sobre el individuo y las demás capas, pudiendo ser más cercanos^(6)^, siendo caracterizados por los dominios individuales como: características individuales, sexo y factores hereditarios, o más externos, y los macrodeterminantes, ejemplificados por las condiciones económicas, culturales y ambientales^(4)^. Estos componentes, que incluyen las características individuales, los factores estructurales y las políticas públicas, modifican la salud, su exposición a la enfermedad y el acceso a los cuidados necesarios^(7)^.

Se sabe que las condiciones de salud infantil pueden verse afectadas por las circunstancias perinatales, incluyendo las condiciones asistenciales, nutricionales, socioeconómicas y culturales, las cuales influyen en los resultados del desarrollo a corto y largo plazo, especialmente en la primera infancia^(8-9)^. En este contexto, el crecimiento, el comportamiento y el desarrollo infantil no están determinados únicamente por mecanismos biológicos, sino también por la interacción continua entre las características individuales de los niños, de los padres y del contexto en el que están inmersos^(8-9)^. Esta interacción es particularmente relevante en la prematuridad, dado que la prevalencia de problemas conductuales y emocionales en niños nacidos pretérmino está asociada no solo a las características intrínsecas de la prematuridad, sino a las diversas características y comportamientos familiares^(10-11)^.

Los primeros dos años del niño, recorte temporal de la infancia definido para esta revisión, se consideran esenciales para el crecimiento y el desarrollo de competencias y habilidades, ya que es en este periodo cuando ocurre el mayor crecimiento cerebral^(12)^. Por lo tanto, es esencial proporcionar un ambiente acogedor, oportunidades de aprendizaje, estímulos e interacciones que contribuyan al desarrollo infantil y que puedan repercutir a lo largo de la vida^(12)^. Al considerar que la prematuridad y la hospitalización en la Unidad de Cuidados Intensivos Neonatales (UCIN) son factores de riesgo para el crecimiento y el desarrollo, es fundamental que las familias reciban el seguimiento en los servicios de salud para la vigilancia de la salud e intervenciones tempranas, con el fin de minimizar los posibles perjuicios causados a los niños^(12-13)^.

Al reconocer la magnitud del nacimiento pretérmino en el contexto de la salud pública global, se ha investigado sobre sus implicaciones en la vida del niño y sus familiares^(13-14)^, especialmente desde las perspectivas individuales, biológicas, enfocadas en factores específicos de los DSS, y en la morbimortalidad infantil^(15)^. Estos hallazgos ofrecen una comprensión limitada sobre los aspectos que pueden ser modificados y contribuyen aún de forma limitada a los avances en el cuidado de los niños nacidos pretérmino. Dada la complejidad de la determinación social de la salud, es importante aclarar las interferencias de factores como: educación, vecindario, ambiente, red de apoyo social y acceso a los servicios de salud en la vida de niños nacidos pretérmino en los primeros dos años de vida. Estos factores tienen el potencial de influir en los resultados en salud, tales como la mortalidad, morbilidad, esperanza de vida, condición de salud, gastos en salud y limitaciones funcionales^(16)^.

Dado lo expuesto, se parte del entendimiento de que los dominios que se constituyen como DSS ofrecen una base para una investigación ampliada y contribuyen a explorar de forma más integral los efectos del modo de vivir y de enfermar de niños nacidos pretérmino en los primeros dos años de vida, considerando el contexto al que están expuestos^(17)^.

La *scoping review* busca mapear las evidencias sobre la influencia de los DSS en la salud de niños nacidos pretérmino en los primeros dos años. Aunque los factores biológicos de la prematuridad están bien documentados, las desigualdades sociales y estructurales aún son poco exploradas de forma integrada. Este enfoque permite captar la complejidad de los DSS, identificar las brechas en el conocimiento y orientar futuras investigaciones, ampliando la comprensión sobre su impacto en el desarrollo y en la calidad de vida de estos niños y sus familias. Así, el objetivo de este estudio fue mapear el conocimiento científico sobre las dimensiones de los DSS en la salud de niños nacidos pretérmino en los primeros dos años de vida.

## Método

### Tipo de estudio

Se trata de una *Scoping review* orientada por el *Joanna Briggs Institute* (JBI), *Manual for Evidence Synthesis* ^(18)^ y por la guía internacional *Preferred Reporting Items for Systematic Reviews and Meta-Analyses extension for Scoping Reviews* (PRISMA-ScR)^(19)^, conducida en cinco etapas: elaboración de la pregunta de investigación, identificación de los estudios relevantes, selección de los estudios, análisis y síntesis de los datos. Esta metodología tiene como objetivo mapear las evidencias disponibles e identificar lagunas en el conocimiento^(20)^. Esta revisión presenta los resultados de una de las preguntas de investigación elaboradas en el protocolo de revisión, que fue registrado en la plataforma *Open Science Framework* (OSF) y puede ser accedido en el enlace https://osf.io/p724a/ con DOI 10.17605/OSF.IO/P724A.

La pregunta orientadora de esta revisión fue elaborada según el mnemónico Población, Concepto y Contexto (PCC), siendo población: los niños nacidos pretérmino en los primeros dos años de vida; el concepto: dimensiones de los DSS; y el contexto: salud de los niños nacidos pretérmino en los primeros dos años de vida.

La pregunta orientadora fue: ¿Cuáles dimensiones de los DSS han sido abordadas en la literatura sobre la salud de los niños nacidos pretérmino en los primeros dos años de vida? Se elaboraron preguntas secundarias para apoyar el logro de los objetivos, siendo ellas: ¿Cuáles son los enfoques metodológicos de los estudios? ¿Cuáles son las características de la población estudiada? ¿Qué factores han sido investigados como DSS? ¿Qué dimensiones de los DSS han sido contempladas en los estudios? ¿Qué resultados en salud infantil han sido relacionados con los DSS?

### Criterios de selección

Se consideraron criterios de inclusión: estudios originales en inglés, portugués o español, publicados en los últimos 13 años (2011 a 2024), que consideraran niños nacidos pretérmino en los primeros dos años de vida en el contexto domiciliario, y que abordaran los DSS y su relación con la salud en ese período. El recorte temporal fue definido con el objetivo de mapear el conocimiento en un período más reciente, que pudiera reflejar las condiciones de vida de la población estudiada. Se excluyeron los artículos de opinión, editoriales, cartas, revisiones de literatura, la literatura gris y los estudios que no respondieran a la pregunta de investigación.

### Variables del estudio

Las variables recolectadas para la caracterización de cada estudio engloban el título, tipo de estudio, país, año de publicación, objetivo, dimensiones evaluadas y muestra. También se identificaron variables infantiles que comprenden los códigos y subcódigos de los DSS como condiciones económicas, sociales, culturales y ambientales (socioeconómicas; ambientales; culturales; políticos y políticas públicas); condiciones de vida y trabajo (acceso a la alimentación; acceso a los servicios esenciales; educación; condición de la vivienda; y condición de trabajo); redes sociales y de apoyo comunitario (redes de apoyo); factores conductuales y de estilo de vida del individuo (cuidado familiar; cuidado canguro; apego; lactancia materna; y hábitos no saludables); características del individuo (condiciones de salud; edad, sexo y factores hereditarios; factores psicológicos; y factores étnicos y raciales); y resultados en salud infantil (desarrollo; conductual; crecimiento; afecciones; mortalidad infantil; y demandas por servicios de salud).

### Fuentes de información y estrategia de búsqueda

La estrategia de búsqueda fue construida por una bibliotecaria y utilizada en cinco bases de datos, MEDLINE/*PubMed*, *Biblioteca Virtual em Saúde* (BVS), *Excerpta Medica Database* (EMBASE), *Scopus* y *Web of Science*. Esta etapa se realizó en marzo de 2024 utilizando las estrategias de búsqueda descritas en la Figura 1.

**Figura 1 -:** Descripción de las estrategias de búsqueda utilizadas en cada base de datos. Belo Horizonte, MG, Brasil, 2024

**Base**	**Estrategia de búsqueda**
BVS*	(“Recém-Nascido Prematuro” OR “Infant, Premature” OR “Recien Nacido Prematuro” OR prématuré OR “Bebê Prematuro” OR “Bebês Prematuros” OR prematuridade OR prematuro OR prematuros OR “Recém-Nascidos Prematuros” OR “Nascimento Prematuro” OR “Premature Birth” OR “Nacimiento Prematuro” OR “Naissance prématurée” OR “Premature Infant” OR “Premature Infants”) AND (“Determinantes Sociais da Saúde” OR “Social Determinants of Health” OR “Determinantes Sociales de la Salud” OR “Déterminants sociaux de la santé” OR “Determinante de Saúde” OR “Determinantes Estruturais da Saúde” OR “Determinantes Estruturais de Saúde” OR “Determinantes Sociais de Saúde” OR “Fatores Socioeconômicos” OR “Socioeconomic Factors” OR “Factores Socioeconómicos” OR “Facteurs socioéconomiques” OR “Aspectos Socioeconômicos” OR “Fatores Sociais” OR “Social Factors” OR “Factores Sociales” OR “Fatores Culturais” OR “Cultural Factors” OR “Factores Culturales” OR “Facteurs Culturels” OR “Fatores Econômicos” OR “Economic Factors” OR “Factores Económicos” OR “Facteurs économiques” OR “Fatores Raciais” OR “Race Factors” OR “Factores Raciales” OR “Facteurs raciaux” OR or “Condições Sociais” OR “Social Conditions” OR “Condiciones Sociales” OR “Conditions sociales” OR “Condição Social” OR “Condições de Vida” OR “Política de Saúde” OR “Health Policy” OR “Política de Salud” OR “Politique de santé” OR “Health Social Determinant” OR “Health Social Determinants” OR “Health Structural Determinant” OR “Health Structural Determinants” OR “Structural Determinants of Health” OR “Living Condition” OR “Living Conditions” OR “Social Condition”) AND (“Cuidado do Lactente” OR “Infant Care” OR “Cuidado del Lactante” OR “Soins du nourrisson” OR família OR family OR familia OR famille) AND ( db:(“LILACS” OR “IBECS” OR “BDENF” OR “INDEXPSI” OR “BINACIS” OR “BBO” OR “CUMED” OR “LIPECS” OR “MedCarib” OR “SES-SP” OR “colecionaSUS” OR “PAHOIRIS” OR “BIGG” OR “MULTIMEDIA” OR “CidSaude” OR “PAHO” OR “PIE” OR “PREPRINT-MEDRXIV” OR “PREPRINT-SCIELO” OR “VETINDEX”))
**MEDLINE/** *PubMed*	(“Infant, Premature” OR “Premature Birth” OR “Premature Infant” OR “Premature Infants”) AND (“Social Determinants of Health” OR “Socioeconomic Factors” OR “Social Factors” OR “Cultural Factors” OR “Economic Factors” OR “Race Factors” OR “Social Conditions” OR “Health Policy” OR “Health Social Determinant” OR “Health Social Determinants” OR “Health Structural Determinant” OR “Health Structural Determinants” OR “Structural Determinants of Health” OR “Living Condition” OR “Living Conditions” OR “Social Condition”) AND (“Infant Care” OR Family)
***Scopus*** (Vía *Portal CAPES* **^†^** )	(“Infant, Premature” OR “Premature Birth” OR “Premature Infant” OR “Premature Infants”) AND (“Social Determinants of Health” OR “Socioeconomic Factors” OR “Social Factors” OR “Cultural Factors” OR “Economic Factors” OR “Race Factors” OR “Social Conditions” OR “Health Policy” OR “Health Social Determinant” OR “Health Social Determinants” OR “Health Structural Determinant” OR “Health Structural Determinants” OR “Structural Determinants of Health” OR “Living Condition” OR “Living Conditions” OR “Social Condition”) AND (“Infant Care” OR Family)
***Web of Science*** (Vía *Portal CAPES* **^†^** )	(“Infant, Premature” OR “Premature Birth” OR “Premature Infant” OR “Premature Infants”) AND (“Social Determinants of Health” OR “Socioeconomic Factors” OR “Social Factors” OR “Cultural Factors” OR “Economic Factors” OR “Race Factors” OR “Social Conditions” OR “Health Policy” OR “Health Social Determinant” OR “Health Social Determinants” OR “Health Structural Determinant” OR “Health Structural Determinants” OR “Structural Determinants of Health” OR “Living Condition” OR “Living Conditions” OR “Social Condition”) AND (“Infant Care” OR Family)
**EMBASE** ^‡^ (Vía *Portal CAPES* **^†^** )	(prematurity) and (‘social determinants of health’ or socioeconomics or ‘social aspect’ or ‘cultural factor’ or ‘economic aspect’ or ‘social status’ or ‘health care policy’) and (‘infant care’ or family)

*BVS = *Biblioteca Virtual em Saúde*; ^†^CAPES = *Coordenação de Aperfeiçoamento de Pessoal de Nível Superior*; ^‡^EMBASE = *Excerpta Medica Database*

### Selección de las fuentes de evidencia y proceso de extracción de los datos

El proceso de selección de los artículos incluidos fue realizado por dos investigadoras independientes y apoyado por el *software Rayyan*. Inicialmente, se excluyeron los estudios duplicados, se leyeron los títulos y resúmenes de los demás artículos siguiendo los criterios de elegibilidad. Los estudios restantes se leyeron en su totalidad, siguiendo los mismos criterios. Las divergencias identificadas durante el proceso de selección fueron analizadas por una tercera investigadora.

### Análisis de los datos

Los datos de los artículos se extrajeron mediante un instrumento de codificación elaborado a partir del modelo de DSS^(6)^, alineado a los objetivos del artículo y al PCC. Este instrumento fue estructurado en el *software* MaxQDA y orientó la búsqueda de información para la recopilación de las variables de caracterización del estudio, así como de los códigos y subcódigos de los DSS.

El código condiciones económicas, sociales, culturales y ambientales se refiere a las condiciones económicas, culturales y ambientales de la sociedad^(4)^, y explora, principalmente, el contexto de cuidado en el que se encuentra el niño. Incluye los subcódigos: factores socioeconómicos (disponibilidad o no de recursos y oportunidades materiales); factores culturales (valores, ideales, intereses y comportamientos de un determinado grupo social; incluye religión); factores políticos y políticas públicas (políticas públicas que buscan la reducción de las desigualdades sociales, que promuevan cambios de comportamiento, acceso facilitado a alimentos saludables, programas que mejoren el acceso a la salud y a la educación de la población. Se consideran también los programas de asistencia social, institucionalización del cuidado^(4)^).

Las condiciones de vida y trabajo esclarecen, sobre todo, el contexto de cuidado al que el niño está expuesto, y se refieren a la disponibilidad de alimentos y al acceso a los ambientes y a los servicios esenciales, como la salud y la educación^(4)^. Este código incluye los subcódigos: acceso a la alimentación (disponibilidad y acceso a una alimentación adecuada); acceso a los servicios esenciales (servicios de educación y de salud); educación (nivel de escolaridad e instrucción de los padres y familiares de los niños pretérmino); condiciones de habitabilidad (condiciones de la vivienda, incluyendo localización, estructura y saneamiento básico); y condiciones de trabajo (ocupación del familiar; si posee un trabajo remunerado o está desempleado)^(4)^.

Las redes sociales y de apoyo comunitario corresponden a las redes comunitarias y de apoyo, lazos familiares y religiosos^(7)^. Los factores conductuales y de estilo de vida del individuo incluyen los subcódigos: cuidado familiar (cuidado al niño ofrecido por la familia en el ámbito domiciliario; cuidado canguro, contacto piel a piel, que comienza de forma temprana y creciente desde el toque, evolucionando hasta la posición canguro^(21)^; apego, vínculo, lazos duraderos establecidos entre los bebés y las madres o cuidadores^(21)^); lactancia materna (práctica de la lactancia materna); y hábitos no saludables (hábitos de vida de los cuidadores que pueden perjudicar la salud infantil, como el consumo de alcohol y otras drogas, mala alimentación y sedentarismo).

Las características de los individuos comprenden las condiciones de salud (circunstancias en la salud del recién nacido que requieren respuestas a través de la asistencia sanitaria, por ejemplo, diagnósticos médicos asignados); edad, sexo y factores hereditarios (factores asociados a la edad de los individuos, al sexo y a la herencia); factores psicológicos (abarca los aspectos de la salud mental de los individuos presentes en el contexto familiar que pueden influir en la salud infantil); y factores étnicos y raciales (factores relacionados con los grupos étnicos y raciales). Por último, los resultados en salud del niño fueron identificados cuando se relacionaron con las condiciones de salud, el crecimiento y el desarrollo neuropsicomotor derivado de la influencia de los DSS.

La muestra final de los artículos fue importada al *software* MaxQDA versión 2022, el cual soportó la codificación de la información. Este proceso fue realizado de forma independiente por dos autoras de este estudio, y los fragmentos codificados fueron validados por una tercera investigadora.

## Resultados

La Figura 2 presenta el proceso de selección de los artículos. Se importaron inicialmente 3.218 estudios, de los cuales 160 fueron excluidos por duplicidad. Tras la lectura de títulos y resúmenes, se excluyeron 2.991, quedando 67 estudios para la lectura íntegra y la realización de la búsqueda inversa, siendo necesario leer 11 estudios adicionales. Uno de los estudios no se encontró en su totalidad; de este modo, se leyeron 77 estudios en su totalidad y, de estos, 31 conformaron la muestra final.

**Figura 2 -:** Diagrama de flujo del proceso de selección de los artículos, según PRISMA-SCR^†^. Belo Horizonte, MG, Brasil, 2024

*n = Número; ^†^PRISMA-SCR = *Preferred Reporting Items for Systematic reviews and Meta-Analyses extension for Scoping Reviews*

Los 31 artículos incluidos en este estudio se presentan en la figura 3, incluyendo el título, tipo de estudio/país/año de publicación, objetivo, dimensiones evaluadas y muestra.

Entre los artículos analizados, la mayoría (80,65%) presentó un enfoque cuantitativo. Respecto a los continentes de origen de la publicación, 19 estudios se realizaron en América del Norte (A3, A4, A5, A6, A8, A9, A10, A11, A12, A13, A16, A17, A19, A20, A22, A23, A25, A27, A31), seis en Europa (A1, A2, A7, A18, A28, A29), cuatro en América del Sur (A14, A15, A21, A24) y dos en Asia (A26, A30). El año con mayor número de publicaciones fue 2021, con nueve artículos, mientras que 2014 no registró ninguna publicación. En los demás años, el promedio de publicaciones fue de 2,38 artículos.

**Figura 3 -:** Caracterización de los estudios incluidos. Belo Horizonte, MG, Brasil, 2024

**Código del artículo**	**Título**	**Tipo de estudio, país, (año de publicación)**	**Objetivo**	**Dimensiones evaluadas**	**Muestra**
A01 ^( 22 )^	*Psychological distress and early lactation performance in mothers of late preterm infants*	Caso control, Italia, (2011).	Examinar la relación entre el sufrimiento psicológico de madres que tuvieron parto pretérmino tardío y el desempeño en el inicio de la lactancia.	Factores conductuales y de estilo de vida del individuo; características del individuo.	Grupo caso: 42 madres de RNPT* nacidos con EG ^†^ entre 34 y 36 semanas. Grupo control: 42 madres de RN ^‡^ a término.
A02 ^( 23 )^	*Parenting stress in mothers after very preterm birth and the effect of the Infant* *Behavioural Assessment and Intervention Program*	Caso control, Holanda, (2011).	Examinar el estrés parental materno como un resultado secundario del *Infant Behavioral Assessment and Intervention Program* (IBAIP) ^§^ .	Condiciones económicas, sociales, culturales y ambientales; condiciones de vida y trabajo; redes sociales y de apoyo de la comunidad; factores conductuales y de estilo de vida del individuo; características del individuo.	Grupo intervención: 86 RNPT* (nacidos con < 32 semanas de EG ^†^ y/o peso al nacer <1500 gramos) y 72 padres. Grupo control: 90 RNPT* (nacidos con < 32 semanas de IG ^†^ y/o peso al nacer <1500 gramos) y 79 padres.
A03 ^( 24 )^	*Evaluation of the ability of neurobiological, neurodevelopmental and socio-economic variables to predict cognitive outcome in premature infants*	Longitudinal, Estados Unidos, (2011).	Reevaluar la eficacia del *Neurobiologic Risk Score* (NBRS) ^||^ y del *Neurodevelopmental Risk Exam* (NRE) ^¶^ y evaluar el papel predictivo relativo de factores de riesgo socioeconómicos, tanto de forma aislada como combinados con factores de riesgo biológicos y de desarrollo conocidos.	Condiciones de vida y trabajo; redes sociales y de apoyo de la comunidad; características del individuo.	129 RNPT* (nacidos con < 32 semanas de EG ^†^ ).
A04 ^( 25 )^	*Effect of ethnicity and race on cognitive and language testing at 18 – 22 months in extremely preterm infants*	Cohorte retrospectiva, Estados Unidos, (2012).	Evaluar la relación de raza/etnia con los puntajes cognitivos y de lenguaje en la *Bayley Scales of Infant and Toddler Development, Third Edition* (BSID-III)** en bebés prematuros extremos (<28 semanas de IG ^†^ ).	Condiciones de vida y trabajo; características del individuo.	865 RN ^‡^ extremadamente prematuros (nacidos con < 28 semanas de EG ^†^ ).
A05 ^( 26 )^	*Parenting stress, social support, and mother-child interactions in families of multiple and singleton preterm toddlers*	Longitudinal, Estados Unidos, (2012).	Investigar el apoyo familiar como un potencial moderador del estrés parental materno en familias con hijos prematuros, tanto múltiples como únicos.	Condiciones económicas, sociales, culturales y ambientales; condiciones de vida y trabajo; redes sociales y de apoyo de la comunidad; factores conductuales y de estilo de vida del individuo; características del individuo.	153 familias y RNPT* (nacidos con < 37 semanas de EG ^†^ ).
A06 ^( 27 )^	*Impact of maternal education on cognitive and language scores at 18 to 24 months among extremely preterms neonates*	Cohorte, Canadá, (2013).	Explorar la asociación entre los niveles de escolaridad materna y los puntajes compuestos cognitivos y de lenguaje de la Escala Bayley III, a los 18 a 24 meses de edad corregida en bebés prematuros extremos nacidos con <29 semanas de IG ^†^ .	Condiciones de vida y trabajo; redes sociales y de apoyo de la comunidad; características del individuo.	457 familias y RN ^‡^ extremadamente prematuros (nacidos con < 29 semanas de EG ^†^ ).
A07 ^( 28 )^	*One-year neurodevelopmental outcome of very and late preterm infants: risk factors and correlation with maternal stress*	Longitudinal, Italia, (2015).	Describir el perfil de desarrollo de lactantes pretérmino tardío, comparar el desarrollo cognitivo, lingüístico y motor en lactantes pretérmino tardío y muy pretérmino a los 12 meses de edad corregida; explorar la relación entre las medidas de resultados del desarrollo y el estrés materno en ambos subgrupos; analizar la influencia de los factores de riesgo neonatales y de las características sociodemográficas maternas en las medidas de los resultados del desarrollo.	Condiciones de vida y trabajo; características del individuo.	Madres y 79 RNPT* - 39 prematuros tardíos (nacidos entre 33-36 semanas de EG ^†^ ) - 40 muy prematuros (nacidos con ≤ 32 semanas de EG ^†^ ).
A08 ^( 29 )^	*Neurodevelopmental profile, growth, and psychosocial environment of preterm infants with difficult feeding behavior at age 2 years*	Cohorte, Estados Unidos, (2015).	Examinar la asociación de comportamientos alimentarios difíciles en bebés muy prematuros a los dos años con resultados de crecimiento y neurodesarrollo, y factores familiares y de funcionamiento.	Condiciones de vida y trabajo; redes sociales y de apoyo de la comunidad; características del individuo.	104 RNPT* (nacidos con ≤ 30 semanas de EG ^†^ ).
A09 ^( 30 )^	*Developmental outcomes of extremely preterm infants born to adolescent mothers*	Cohorte retrospectiva, Estados Unidos, (2015).	Evaluar los resultados cognitivos, de lenguaje y de comportamiento de bebés extremadamente prematuros nacidos de madres adolescentes (<20 años) en comparación con bebés extremadamente prematuros nacidos de madres adultas (≥20 años); identificar los factores de riesgo socioeconómicos que afectan los resultados.	Condiciones económicas, sociales, culturales y ambientales; condiciones de vida y trabajo; redes sociales y de apoyo de la comunidad; factores conductuales y de estilo de vida del individuo; características del individuo.	1.934 RNPT* (nacidos con 27 semanas) - 211 RNPT* de madres adolescentes - 1.723 RNPT* de madres adultas.
A10 ^( 31 )^	*Health care use outcomes of an integrated hospital-to-home mother-preterm infant intervention*	Estudio controlado aleatorizado, Estados Unidos, (2016).	Comparar los cuidados de salud desde el alta hospitalaria hasta las seis semanas de edad corregida en dos grupos de díadas madre-bebé prematuro: aquellos que recibieron una intervención, *Hospital to Home: Optimizing Premature Infant’s Environment* (H-HOPE) ^††^ y un grupo de control de atención.	Condiciones económicas, sociales, culturales y ambientales; condiciones de vida y trabajo; redes sociales y de apoyo de la comunidad; características del individuo.	147 madres y RNPT* (nacidos entre 29 y 34 semanas de EG ^†^ ).
A11 ^( 32 )^	*Life after discharge: what parents of preterm infants say about their transition to home*	Descriptivo y exploratorio, Estados Unidos, (2016).	Explorar y describir las experiencias de los padres de bebés prematuros después del alta hospitalaria.	Condiciones económicas, sociales, culturales y ambientales; condiciones de vida y trabajo; redes sociales y de apoyo de la comunidad; factores conductuales y de estilo de vida del individuo; características del individuo.	52 padres de RNPT* (nacidos < 37 semanas).
A12 ^( 33 )^	*A qualitative study: mothers of late preterm infants relate their experiences of community-based care*	Descriptivo con enfoque fenomenológico, Canadá, (2017).	Explorar la experiencia de las madres al cuidar de sus bebés pretérmino tardíos en la comunidad.	Condiciones económicas, sociales, culturales y ambientales; condiciones de vida y trabajo; redes sociales y de apoyo de la comunidad; factores conductuales y de estilo de vida del individuo; características del individuo.	11 madres de RNPT* tardíos (nacidos entre 34 y 37 semanas de EG ^†^ ).
A13 ^( 34 )^	*Homecare and healthcare utilization errors post–neonatal intensive care unit discharge*	Cohorte prospectiva, Estados Unidos, (2017).	Identificar y describir los errores en los cuidados domiciliarios y en la utilización de los servicios de salud en bebés de alto riesgo tras el alta de la UTIN ^‡‡^ .	Condiciones económicas, sociales, culturales y ambientales; condiciones de vida y trabajo; redes sociales y de apoyo de la comunidad; factores conductuales y de estilo de vida del individuo; características del individuo.	241 RNPT* - 163 nacidos con <32 semanas de EG ^†^ o 1.500 gramos - 78 nacidos con >32 semanas de EG ^†^ y más de 1500 gramos.
A14 ^( 35 )^	Situación socioeconómica familiar y neurodesarrollo de prematuros de muy bajo peso al nacer a los dos años	Cohorte retrospectiva, Chile, (2018).	Caracterizar las familias en las que crecen los prematuros de muy bajo peso al nacer con nivel socioeconómico bajo y medio-bajo; explorar la posible asociación entre las características sociofamiliares y el neurodesarrollo a los dos años.	Condiciones económicas, sociales, culturales y ambientales; condiciones de vida y trabajo; redes sociales y de apoyo de la comunidad; factores conductuales y de estilo de vida del individuo; características del individuo.	162 familias de RNPT* de muy bajo peso al nacer (nacidos < 32 semanas de EG ^†^ o < 1500 gramos).
A15 ^( 36 )^	Intervención basada en rutinas como apoyo a la participación familiar para el desarrollo del lenguaje en bebés prematuros	Estudio de caso, Ecuador, (2020).	Presentar el impacto que una Intervención Basada en Rutinas (IBR) ^§§^ puede tener en la participación de la familia para el desarrollo del lenguaje de sus bebés prematuros.	Condiciones económicas, sociales, culturales y ambientales; condiciones de vida y trabajo; redes sociales y de apoyo de la comunidad; factores conductuales y de estilo de vida del individuo; características del individuo.	11 familias y 11 RNPT* (nacidos con < 37 semanas de EG ^†^ ).
A16 ^( 37 )^	*Disparities in preterm infant emergency room utilization and rehospitalization by maternal immigrant status*	Exploratorio, Estados Unidos, (2020).	Evaluar los efectos de la condición de madre inmigrante y de los factores de riesgo en las tasas de visitas al servicio de urgencias y readmisiones de bebés prematuros hasta 90 días después del alta.	Condiciones económicas, sociales, culturales y ambientales; condiciones de vida y trabajo; redes sociales y de apoyo de la comunidad; factores conductuales y de estilo de vida del individuo; características del individuo.	732 madres y 866 RNPT* (nacidos con < 37 semanas de EG ^†^ ). - 176 madres inmigrantes y 203 RNPT* - 556 madres nativas y 663 RNPT*.
A17 ^( 38 )^	*Home visiting for Neonatal Intensive Care Unit (NICU)* ^||||^ *graduates: impacts of Following Baby Back Home*	Cohorte, Estados Unidos, (2021).	Examinar si la intervención *Following Baby Back Home* (FBBH) ^¶¶^ , en comparación con el grupo control, demostró un aumento en los cuidados de salud e inmunizaciones y menor mortalidad infantil desde el alta de la UCIN ^‡‡^ hasta el primer año de vida.	Condiciones económicas, sociales, culturales y ambientales; condiciones de vida y trabajo; redes sociales y de apoyo de la comunidad; factores conductuales y de estilo de vida del individuo; características del individuo.	498 RNPT* de bajo peso al nacer (< 2500 gramos y < 37 semanas de EG ^†^ ).
A18 ^( 39 )^	*The effects of prematurity and socioeconomic deprivation on* *early speech perception: a story of two different delays*	Longitudinal, Reino Unido, (2020).	Comprender la interacción entre la maduración física/cognitiva y la exposición ambiental al lenguaje en el desarrollo inicial del lenguaje.	Condiciones económicas, sociales, culturales y ambientales; condiciones de vida y trabajo.	38 RNPT* (nacidos ≤ 33 semanas de EG ^†^ ).
A19 ^( 40 )^	*Maternal post-traumatic stress and depression symptoms and outcomes after NICU* ^||||^ *discharge in a low-income sample: a cross-sectional study*	Transversal, Estados Unidos, (2021).	Describir la prevalencia de exámenes positivos para estrés postraumático (PTS)*** agudo y síntomas de depresión entre las familias de bajos ingresos después del alta de la UCIN ^‡‡^ . Examinar la asociación ajustada del PTS*** y los síntomas de depresión con los resultados en salud y neurodesarrollo infantil. Evaluar la asociación ajustada del PTS*** y los síntomas de depresión con la calidad de vida materna.	Condiciones económicas, sociales, culturales y ambientales; condiciones de vida y trabajo; características del individuo.	150 familias de RNPT* (nacidos < 37 semanas de EG ^†^ ).
A20 ^( 41 )^	*The association of care transitions measure-15 score and outcomes after discharge from the NICU* ^||||^	Transversal, Estados Unidos, (2021).	Describir los puntajes del *Care Transitions Measure* (CTM) ^†††^ entre los cuidadores de prematuros después del alta de la UCIN ^‡‡^ . Describir la asociación de las puntuaciones del CTM ^†††^ con readmisiones, inscripciones en programas de asistencia pública y puntajes de calidad de vida del cuidador.	Condiciones económicas, sociales, culturales y ambientales; condiciones de vida y trabajo; características del individuo.	169 padres de RNPT* (nacidos con <37 semanas de EG ^†^ ).
A21 ^( 42 )^	*Factors associated with the socioemotional development of preterm infants*	Observacional longitudinal, Brasil, (2021).	Comparar la diferencia en el desarrollo socioemocional entre el alta hospitalaria y a los seis meses de edad corregida en lactantes nacidos ≤32 semanas y evaluar el impacto de factores maternos e infantiles en la tasa de desarrollo socioemocional en un período de seis meses.	Condiciones económicas, sociales, culturales y ambientales; condiciones de vida y trabajo; redes sociales y de apoyo de la comunidad; factores conductuales y de estilo de vida del individuo; características del individuo.	72 madres y RNPT* (nacidos con ≤ 32 semanas de EG ^†^ ).
A22 ^( 11 )^	*Maternal depressive symptoms, poverty, and young motherhood increase the odds of early depressive and anxiety disorders for children born prematurely*	Observacional longitudinal, Estados Unidos, (2021).	Identificar la prevalencia de trastornos depresivos y de ansiedad a los dos años entre los niños nacidos prematuramente y determinar en qué medida la pobreza, los síntomas depresivos maternos o la maternidad joven aumentan la probabilidad de dichos trastornos.	Condiciones económicas, sociales, culturales y ambientales; condiciones de vida y trabajo; redes sociales y de apoyo de la comunidad; factores conductuales y de estilo de vida del individuo; características del individuo.	105 madres y RNPT* (nacidos con < 37 semanas de EG ^†^ ).
A23 ^( 43 )^	*Risk factors for re-hospitalization following neonatal discharge of extremely preterm infants in Canada*	Cohorte prospectiva, Canadá, (2021).	Identificar las características neonatales, sociodemográficas y geográficas que predicen la readmisión en recién nacidos prematuros extremos canadienses.	Condiciones económicas, sociales, culturales y ambientales; condiciones de vida y trabajo; características del individuo.	2.275 RNPT* (nacidos entre 22 y 28 semanas de EG ^†^ ).
A24 ^( 44 )^	*Risk factors for fine and gross motor development in preterm and term infants*	Observacional, Brasil, (2021).	Investigar la asociación de factores sociodemográficos, obstétricos y psicosociales con el retraso en el desarrollo motor fino y grueso en lactantes prematuros y a término, en el rango de edad de tres meses y un día a 12 meses y 29 días.	Condiciones económicas, sociales, culturales y ambientales; condiciones de vida y trabajo; redes sociales y de apoyo de la comunidad; factores conductuales y de estilo de vida del individuo; características del individuo.	RN ^‡^ a término y pretérmino (< 37 semanas). Etapa 1 (3 – cuatro meses y 29 días): 103 a término y 62 RNPT*; Etapa 2 (8 – nueve meses y 29 días): 84 a término y 46 RNPT*; Etapa 3 (11- 12 meses y 29 días): 69 a término y 33 RNPT*.
A25 ^( 45 )^	*Parent-reported health status of preterm survivors in a Canadian cohort*	Cohorte prospectiva, Canadá, (2022).	Describir el estado de salud reportado por los padres en una gran cohorte nacional de niños nacidos prematuramente extremos en edad preescolar e identificar las variables clínicas y sociodemográficas asociadas al estado de salud.	Redes sociales y de apoyo de la comunidad; factores conductuales y de estilo de vida del individuo; características del individuo.	811 RNPT* (nacidos < 29 semanas de EG ^†^ ).
A26 ^( 14 )^	*Neurodevelopmental outcomes at 6, 12, and 24 months of age in preterm infants with very low birth weights in Taiwan*	Cohorte, Taiwán, (2021).	Identificar los antecedentes perinatales asociados a la deficiencia en el neurodesarrollo en bebés prematuros de muy bajo peso a los 6, 12 y 24 meses de edad y la estabilidad de las evaluaciones del neurodesarrollo.	Condiciones económicas, sociales, culturales y ambientales; condiciones de vida y trabajo; redes sociales y de apoyo de la comunidad; características del individuo.	8.517 RNPT* de muy bajo peso (entre 24 y 32 semanas de EG ^†^ ).
A27 ^( 46 )^	*Association of inotrope use with neurodevelopmental outcomes in infants <29 weeks gestation: a retrospective cohort study*	Cohorte retrospectiva, Canadá, (2021).	Comparar los resultados del neurodesarrollo a los 18-24 meses en bebés prematuros <29 semanas de edad gestacional que recibieron versus aquellos que no recibieron inotrópicos en la primera semana de vida.	Condiciones de vida y trabajo; factores conductuales y de estilo de vida del individuo; características del individuo.	2.615 RNPT* (<29 semanas de EG ^†^ ).
A28 ^( 47 )^	*Association of preterm birth and socioeconomic status with neonatal brain structure*	Cohorte, Reino Unido, (2023).	Investigar asociaciones entre la edad gestacional al nacer y el estatus socioeconómico con la morfología cerebral neonatal.	Condiciones económicas, sociales, culturales y ambientales; condiciones de vida y trabajo; factores conductuales y de estilo de vida del individuo; características del individuo.	170 RNPT* (< 33 semanas de EG ^†^ ) y 91 RN ^‡^ a término.
A29 ^( 48 )^	*Cortisol reactivity and negative affect among preterm infants at 12 months during a mother-infant interaction task*	Longitudinal, Portugal, (2023).	Investigar las correlaciones de la reactividad al cortisol del bebé prematuro y la asociación con el afecto negativo del bebé.	Condiciones económicas, sociales, culturales y ambientales; condiciones de vida y trabajo; factores conductuales y de estilo de vida del individuo; características del individuo.	48 RNPT*
A30 ^( 49 )^	*Early developmental support for preterm infants based on* *exploratory behaviors:a parallel randomized controlled study*	Estudio controlado aleatorizado, Turquía, (2023).	Investigar la eficiencia del nuevo programa de intervención temprana *Explorer Baby* en las habilidades motoras, cognitivas y de lenguaje de bebés prematuros.	Condiciones de vida y trabajo; factores conductuales y de estilo de vida del individuo; características del individuo.	57 RNPT* (< 33 semanas de EG ^†^ ).
A31 ^( 50 )^	*Language outcomes at 18–24 months of preterm infants from US Spanish- and English-speaking homes*	Cohorte retrospectiva, Estados Unidos, (2023).	Evaluar la asociación entre el retraso del lenguaje expresivo y receptivo, de leve a moderado, y el idioma principal (español e inglés) hablado en casa de niños prematuros utilizando el BSID-III ^**^ y examinar la asociación con los factores socioeconómicos.	Condiciones económicas, sociales, culturales y ambientales; condiciones de vida y trabajo; redes sociales y de apoyo de la comunidad; factores conductuales y de estilo de vida del individuo; características del individuo.	1.125 RNPT* (< 32 semanas de EG ^†^ ).

*RNPT = Recién Nacido Pretérmino; ^†^EG = Edad Gestacional; ^‡^RN = Recién Nacido; ^§^IBAIP = *Infant Behavioral Assessment and Intervention Program*; ^||^NBRS = *Neurobiologic Risk Score*; ^¶^NRE = N*eurodevelopmental Risk Exam*; **BSID-III = *Bayley Scales of Infant and Toddler Development, Third Edition*; ^††^H-HOPE = *Hospital to Home: Optimizing Premature Infant’s Environment*; ^‡‡^UCIN = Unidad de Cuidados Intensivos Neonatales; ^§§^IBR = Intervención Basada en Rutinas; ^||||^NICU = *Neonatal Intensive Care Unit*; ^¶¶^FBBH = *Following Baby Back Home*; ***PTS = Estrés Postraumático; ^†††^CTM = *Care Transitions Measure*

El proceso de codificación de los estudios permitió identificar las variables congruentes con las dimensiones de los DSS (Tabla 1) de acuerdo con los códigos y subcódigos descritos en la sección de análisis de los datos.

Los artículos contemplaron todas las macrodimensiones de los DSS: condiciones de vida y trabajo, factores conductuales y de estilo de vida, y características del individuo relativas a los niños nacidos pretérmino en los primeros dos años de vida, siendo este último explorado por todos los artículos.

**Tabla 1 -:** Variables por subcódigos y cuantificadas para los estudios. Belo Horizonte, MG, Brasil, 2024

**Subcódigo**	n*	**%** ^†^ (n*/total)
**Condiciones económicas, sociales, culturales y ambientales**	**22**	**70,97%** ^†^
Factores socioeconómicos	18	58,06% ^†^
Factores culturales	15	48,39% ^†^
Factores ambientales	0	0,00% ^†^
Factores políticos y políticas públicas	7	22,58% ^†^
**Condiciones de vida y trabajo**	**29**	**93,55%** ^†^
Acceso a la alimentación	0	0,00% ^†^
Acceso a servicios esenciales	14	45,16% ^†^
Educación	27	87,10% ^†^
Condición de la vivienda	10	32,26% ^†^
Condición de trabajo	10	32,26% ^†^
**Redes sociales y de apoyo de la comunidad**	**21**	**67,74%** ^†^
Redes de apoyo	21	67,74% ^†^
**Factores conductuales y de estilo de vida del individuo**	**19**	61,29% ^†^
Cuidado familiar	12	38,71% ^†^
Lactancia materna	7	22,58% ^†^
Hábitos no saludables	8	25,81% ^†^
**Características del individuo**	**31**	**100%** ^†^
Condición de salud	31	100% ^†^
Edad, sexo y factores hereditarios	31	100% ^†^
Factores psicológicos	15	48,39% ^†^
Factores étnicos y raciales	16	51,61% ^†^
**Resultados en salud infantil**	**31**	**100%** ^†^
Desarrollo	19	61,29% ^†^
Conductual	5	16,13% ^†^
Crecimiento	4	12,90% ^†^
Afecciones	5	16,13% ^†^
Mortalidad infantil	1	3,23% ^†^
Demandas por servicios de salud	9	29,03% ^†^

*n = Número; ^†^% = Porcentaje de estudios

Las variables investigadas en los artículos analizados se encuentran representadas específicamente de acuerdo con las macro y micro dimensiones de los DSS en la Tabla 2.

**Tabla 2 –:** Variables abordadas en los estudios y resultados en salud de los niños relacionados con los códigos y subcódigos. Belo Horizonte, MG, Brasil, 2024

**Subcódigo**	**Art** ícul **os**	Cant* (n ^†^ **)**	**%** ^‡^ **(n** ^†^ **/total)**
**Factores socioeconómicos**			
Pobreza	A05, A15, A16, A22, A23	5	16,13% ^‡^
Ingreso familiar	A05, A10, A11, A12, A15, A17, A18, A19, A20, A22, A24, A28, A29	13	41,94% ^‡^
Condiciones socioeconómicas	A14, A18, A21, A26, A28	5	16,13% ^‡^
Bienes de consumo duraderos	A05	1	3,23% ^‡^
Índice de riesgo sociodemográfico	A05	1	3,23% ^‡^
**Factores culturales**			
Nacionalidad	A11, A12, A16, A22, A23, A24	6	19,35% ^‡^
Lengua materna	A02, A09, A10, A13, A16, A18, A19, A20, A31	9	29,03% ^‡^
Calidad de vida	A19, A20, A28	3	9,68% ^‡^
**Factores políticos y políticas públicas**			
Programa de cuidado de transición	A11	1	3,23% ^‡^
Programa de suplementación alimentaria	A17, A20	2	6,45% ^‡^
Beneficios sociales	A14, A20, A22	3	9,68% ^‡^
Servicio de protección al niño	A16	1	3,23% ^‡^
Abuso doméstico	A16	1	3,23% ^‡^
Supervisión del Estado	A09	1	3,23% ^‡^
**Acceso a los servicios esenciales**			
Acceso a los servicios de salud (prenatal, maternidad, intervención, especialización)	A09, A11, A16, A20, A24, A31	6	19,35% ^‡^
Acceso a la intervención de salud	A02, A10, A13, A17, A20, A30, A31	7	22,58% ^†^
Asistencia social	A23	1	3,23% ^‡^
Seguro de salud (público o privado)	A09, A11, A16, A17, A31	5	16,13% ^‡^
Acceso a atenciones especializadas o seguimiento ambulatorio	A09, A10, A03, A12, A31	5	16,13% ^‡^
Seguimiento post-alta en la UCIN ^§^	A13	1	3,23% ^‡^
**Educación**			
Nivel de escolaridad de los cuidadores	A02, A03, A04, A05, A06, A07, A08, A09, A10, A11, A12, A13, A14, A16, A17, A18, A19, A20, A22, A23, A24, A26, A27, A28, A29, A30, A31	27	87,10% ^‡^
**Condición de la vivienda**			
Lugar de residencia/vivienda	A09, A10, A14, A17, A23, A28	6	19,35% ^‡^
Número de habitantes/niños en la vivienda/saturación	A09, A10, A14, A16, A23, A24, A29	7	22,58% ^†^
Distancia entre el lugar de residencia y el servicio de salud	A23	1	3,23% ^‡^
Hogar adoptivo	A08	1	3,23% ^‡^
**Condiciones de trabajo**			
Situación de empleo	A08, A10, A14, A15, A23, A24, A25, A27, A28, A29	10	32,26% ^‡^
Ocupación del proveedor	A08	1	3,23% ^‡^
**Redes de apoyo**			
Situación marital del cuidador	A02, A03, A05, A06, A08, A09, A11, A12, A13, A14, A16, A17, A21, A24, A25, A26, A27, A31	18	58,06% ^‡^
Composición/funcionamiento familiar	A09, A22	2	6,45% ^‡^
Apoyo de familiares/amigos	A05, A09, A12, A15	4	12,90% ^‡^
Tipo de cuidador (remunerado/nana o primario, guardería)	A09, A11, A14, A21	4	12,90% ^‡^
Soporte social	A10, A24	2	6,45% ^‡^
**Cuidado familiar**			
Organización/rutina de cuidado	A11, A15, A24, A30	4	12,90% ^‡^
Soporte materno para la resolución de problemas por el niño	A05	1	3,23% ^‡^
Cuidados inadecuados relacionados con la consulta, administración de medicamentos, alimentación, uso de equipos	A13	1	3,23% ^‡^
Cuidado familiar y profesional (ej: guardería, cuidado en casa, cuidadora profesional o nana)	A09, A31	2	6,45% ^‡^
Cuidado canguro	A21, A30	2	6,45% ^‡^
Apego (interacción materno-infantil)	A02, A05, A21, A22, A29, A30	6	19,35% ^‡^
**Lactancia materna**			
Situación de la lactancia	A01, A16, A17, A21, A28, A31	6	19,35% ^‡^
Desafíos en la lactancia (ej: problemas de lactancia, agarre, otros)	A11	1	3,23% ^‡^
Lactancia materna al alta hospitalaria	A16	1	3,23% ^‡^
**Hábitos no saludables**			
Consumo de alcohol	A12, A14	2	6,45% ^‡^
Uso de sustancias/medicamentos durante el embarazo	A16, A24, A25	3	9,68% ^‡^
Tabaquismo	A01, A17, A28	3	9,68% ^‡^
**Condición de salud**			
Peso al nacer	A01, A02, A03, A04, A05, A06, A07, A08, A09, A10, A11, A12, A13, A14, A16, A17, A18, A19, A20, A21, A23, A24, A26, A27, A28, A29, A30, A31	28	90,32% ^‡^
Medidas antropométricas (peso, estatura, perímetro cefálico)	A08, A14, A28, A31	4	12,90% ^‡^
Alteraciones en el crecimiento	A19, A20, A26	3	9,68% ^‡^
Puntaje de Apgar	A01, A05, A06, A10, A12, A17, A26, A27, A29, A31	10	32,26% ^‡^
Vía de parto	A01, A06, A07, A12, A24	5	16,13% ^‡^
Grado de prematuridad	A16, A31	2	6,45% ^‡^
Transferencia neonatal tras el nacimiento	A26	1	3,23% ^‡^
Ictericia	A12	1	3,23% ^‡^
Gemelaridad	A02, A04, A05, A06, A07, A08, A10, A11, A15, A16, A23, A28, A29	13	41,94% ^‡^
Uso de dispositivos (traqueostomía/gastrostomía, soporte para locomoción, uso de oxígeno y ventilación mecánica, CPAP ^||^ )	A03, A05, A14, A17, A19, A20, A23, A24, A26, A29	10	32,26% ^‡^
Reflujo gastroesofágico	A29	1	3,23% ^‡^
Orden de nacimiento	A02, A05	2	6,45% ^‡^
Orden de nacimiento	A26	1	3,23% ^‡^
Uso de oxígeno	A02, A14, A16, A19, A20, A23, A29, A31	8	25,81% ^‡^
Sepsis	A02, A04, A07, A09, A12, A14, A16, A26, A27	9	29,03% ^‡^
Lesión neurológica (ej.: hemorragia intracerebral y/o intraventricular y/o periventricular; leucomalacia periventricular; parálisis cerebral)	A02, A03, A04, A06, A07, A08, A09, A14, A16, A17, A19, A20, A21, A23, A25, A26, A27, A31	18	58,06% ^‡^
Días de vida al alta	A13	1	3,23% ^‡^
Deterioro circulatorio	A26	1	3,23% ^‡^
Tiempo de ventilación mecánica	A09	1	3,23% ^‡^
pH sanguíneo	A03	1	3,23% ^‡^
Convulsiones	A03, A14, A17, A26, A31	5	16,13% ^‡^
Hipotermia	A12,	1	3,23% ^‡^
Hipoglucemia	A03, A12	2	6,45% ^‡^
Deterioro del neurodesarrollo	A03, A25	2	6,45% ^‡^
Evaluación neurológica	A03, A31	2	6,45% ^‡^
Ceguera	A04, A31	2	6,45% ^‡^
Función motora	A04, A30	2	6,45% ^‡^
Tiempo de hospitalización	A05, A07, A09, A13, A15, A16, A21, A23, A24, A26, A27, A29, A30, A31	14	45,16% ^‡^
Alteraciones respiratorias (apnea, enfermedad respiratoria, problema respiratorio, síndrome de dificultad respiratoria, enfermedad pulmonar crónica, displasia broncopulmonar, deficiencia de surfactante)	A04, A05, A06, A07, A09, A12, A16, A17, A19, A20, A23, A25, A26, A27, A29, A30, A31	17	54,84% ^‡^
Alteraciones cardiovasculares	A07, A12, A17	3	9,68% ^‡^
ROP ^¶^ /ROP ^¶^ III-V	A07, A09, A15, A19, A20, A25, A26, A27	8	25,81% ^‡^
Condición de salud al alta hospitalaria	A05, A06, A07, A25	4	12,90% ^‡^
Tipo de alimentación	A24	1	3,23% ^‡^
Dificultades en la alimentación	A08, A12, A24	3	9,68% ^‡^
Uso de esteroides pre/postnatal	A01, A04, A06, A09, A26, A27	6	19,35% ^‡^
Hidrocefalia	A14, A26, A31	3	9,68% ^‡^
Anormalidad cromosómica	A17	1	3,23% ^‡^
Demanda por servicio de salud (≥ 2 citas programadas/mes, atención de rutina o enfermedad, readmisión hospitalaria, cirugías, tiempo de reingreso, diagnóstico post-alta)	A10, A11, A17, A19, A20	5	16,13% ^‡^
Alimentación por sonda	A19, A20, A23	3	9,68% ^‡^
Nivel de desarrollo cognitivo y motor	A22, A30, A31	1	3,23% ^‡^
Nivel de habilidad lingüística	A30, A31	2	6,45% ^‡^
Mortalidad	A27	1	3,23% ^‡^
Morbilidad neonatal o infantil	A10, A22, A26	3	9,68% ^‡^
Alteraciones infecciosas (infección, neumonía, enterocolitis)	A03, A09, A14, A16, A19, A20, A23, A26, A27, A31	10	32,26% ^‡^
Pérdida auditiva/sordera	A04, A31	1	3,23% ^‡^
Enfermedades maternas e historia obstétrica	A01	1	3,23% ^‡^
Enfermedades crónicas	A01	1	3,23% ^‡^
Ruptura prematura de membranas	A09, A26	2	6,45% ^‡^
Desprendimiento prematuro de placenta	A26	1	3,23% ^‡^
Paridad	A01, A09, A10, A12, A13, A16, A21, A24, A26, A31	10	32,26% ^‡^
Aborto	A24	1	3,23% ^‡^
Complicaciones pre y postparto	A24	1	3,23% ^‡^
Hipertensión	A06, A09, A26, A27, A31	5	16,13% ^‡^
*Diabetes Mellitus* / *Diabetes Mellitus* Gestacional	A26, A27	2	6,45% ^‡^
Preeclampsia	A09, A26	2	6,45% ^‡^
Corioamnionitis	A06, A09, A26, A27, A31	5	16,13% ^‡^
Crecimiento intrauterino restringido	A26	1	3,23% ^‡^
Vía de parto	A09, A26, A24, A27, A31	5	16,13% ^‡^
Administración antenatal de corticosteroides	A09, A23, A26, A27	4	12,90% ^‡^
Gemelaridad	A09, A12, A26, A27	4	12,90% ^‡^
Hemorragia preparto	A26	1	3,23% ^‡^
Placenta previa	A26	1	3,23% ^‡^
Administración de sulfato de magnesio prenatal	A27	1	3,23% ^‡^
Tecnología reproductiva asistida	A27	1	3,23% ^‡^
Oligohidramnios y polihidramnios	A26	1	3,23% ^‡^
**Edad, sexo y factores hereditarios**			
Edad/edad gestacional	A01, A02, A03, A04, A05, A06, A07, A08 A09, A10, A11, A12, A13, A14, A15, A16, A17, A18, A19, A20, A21, A22, A23, A24, A25, A26, A27, A28, A29, A30, A31	31	100% ^‡^
Sexo	A02, A03, A04, A05, A06, A07, A08, A09, A10, A12, A13, A14, A15, A16, A17, A18, A21, A22, A23, A24, A25, A26, A27, A28, A29, A30, A31	27	87,10% ^‡^
**Factores psicológicos**			
Depresión y ansiedad	A08, A22	2	6,45% ^‡^
Ansiedad/rasgo	A01, A08, A10, A12, A21, A29	5	16,13% ^‡^
Depresión/síntomas	A01, A05, A08, A10, A12, A19, A21, A22, A29	8	25,81% ^‡^
Estrés/estrés postraumático	A01, A02, A05, A07, A08, A11, A12, A19, A29, A30	10	32,26% ^‡^
Fatiga/exhaustión	A11	1	3,23% ^‡^
Culpa	A11	1	3,23% ^‡^
Dificultad para compartir sentimientos	A11	1	3,23% ^‡^
Aislamiento social/soledad	A11	1	3,23% ^‡^
Diagnóstico de salud mental	A16,	1	3,23% ^‡^
Factores psicológicos (historia de depresión, embarazo planificado, sentimientos sobre la maternidad)	A24	1	3,23% ^‡^
Sufrimiento psicológico	A02	1	3,23% ^‡^
**Factores étnicos y raciales**			
Raza	A04, A05, A09, A10, A13, A16, A17, A19, A20, A22, A31	11	35,49% ^‡^
Etnia	A02, A04, A05, A08, A09, A10, A12, A13, A16, A17, A19, A20, A22, A23, A28, A31	16	51,61% ^‡^
**Resultado en salud de los niños**			
**Desarrollo**	-	**19**	-
Cognitivo	A03, A04, A06, A07, A08, A09, A19, A25, A27, A30, A31	11	-
Lingüístico	A04, A06, A07, A08, A09, A18, A25, A30, A31	9	-
Motor	A07, A08, A09, A19, A24, A25, A27	7	-
Neurológico	A09, A14, A25	3	-
Socioemocional	A08, A09, A21, A22, A29	5	-
General	A15	1	-
Comunicación, socialización y habilidades de la vida diaria	A19, A30, A31	3	-
Desarrollo mental	A26	1	-
Desarrollo psicomotor	A26	1	-
Cerebral	A28	1	-
**Conductual**		**5**	-
Comportamiento infantil	A02, A05, A08, A09, A21	5	-
**Crecimiento**		**4**	-
Perímetro cefálico	A09	1	-
Crecimiento/peso	A09, A11, A12, A13	4	-
**Afecciones**		**5**	-
Dificultades alimentarias	A08	1	-
Parálisis cerebral	A09	1	-
Deficiencias visuales o neurosensoriales/auditivas	A09, A25, A27	3	-
Estado de salud (saludable o no)	A01	1	-
**Mortalidad infantil**	A17	1	-
**Demandas por servicio de salud** (consulta de rutina, inmunización, readmisión, emergencia, sala de urgencias o cuidados intensivos)	A09, A10, A11, A13, A16, A17, A19, A20, A23	9	-

*Cant. = Cantidad; ^†^n = Número; ^‡^% = Porcentaje de estudios; ^§^UCIN = Unidad de Cuidados Intensivos Neonatales; ^||^CPAP = *Continuous Positive Airway Pressure*; ^¶^ROP = Retinopatía de la Prematuridad

En relación con la dimensión de Características Individuales, todos los estudios investigaron las “Condiciones de Salud”, con énfasis en la variable peso al nacer, que fue abordada en 28 artículos (90,32%). Todos los estudios también utilizaron las variables para investigar la dimensión Edad, Sexo y Factores Hereditarios. En esta dimensión, los aspectos menos abordados fueron los “factores psicológicos” en 15 estudios (48,39%) y los “Factores Étnicos y Raciales” abordados en 16 estudios (51,61%).

En cuanto a la dimensión de Condiciones de Vida y Trabajo, 14 artículos (45,16%) abordaron los aspectos relacionados con el “acceso a los servicios esenciales”, mientras que 10 (32,26%) abordaron las “condiciones de habitabilidad”. También se identificaron variables que contemplan los Factores Conductuales y de Estilo de Vida del Individuo, con siete (22,58%) artículos abordando la “lactancia materna” y 12 (38,71%) el “cuidado familiar”. No se identificaron variables que permitieran explorar las dimensiones de los DSS referentes a los “factores ambientales” y al “acceso a la alimentación”.

Los resultados en salud infantil investigados (Tabla 2) estuvieron en su mayoría (n=19) relacionados con el desarrollo, medido a partir de diferentes escalas, tales como la *Bayley Scales of Infant and Toddler Development*, *Third Edition* (BSID-III) (desarrollo lingüístico, cognitivo y motor), *Cognitive Adaptive Test/Clinical Linguistic and Auditory Milestone Scale* (CAT/CLAMS) (desarrollo cognitivo), DENVER (desarrollo motor), *Gross Motor Function Classification System* (GMFCS) y *Bayley Scales of Infant Development*, *Second Edition* (BSID-II) (desarrollo neurológico), *Brief Infant-Toddler Social and Emotional Assessment* (BITSEA) y *Mother-baby Interaction Observation Protocol* (POIMB) (desarrollo socioemocional/conductual), *Ages and Stages Questionnaires* 3 (ASQ-3) (desarrollo general). Del total de estudios, solo cuatro investigaron los resultados relacionados con el crecimiento (peso, perímetro cefálico y crecimiento).

## Discusión

El análisis de los estudios permitió evidenciar los aspectos que se están abordando en las producciones científicas que contribuyen al conocimiento sobre los niños nacidos pretérmino en los primeros dos años de vida y que consideran las dimensiones de los DSS^(6)^. Aunque los estudios abordan variables que contribuyen a conocer los DSS, ninguno emplea el referente teórico^(6)^ para su desarrollo. La utilización de este referente puede contribuir a la comprensión de los modos de vivir y enfermar de los niños. Se sabe que comprender el contexto en el que el niño está inserto es fundamental para un abordaje integral, ya que posibilita una visión ampliada y permite la formulación de cuidados individualizados más eficaces y el planeamiento de políticas públicas.

Con relación a la dimensión Condiciones Económicas, Sociales, Culturales y Ambientales, se identifica que la variable factor socioeconómico fue abordada en 18 artículos, buscando medir especialmente el ingreso familiar, la pobreza y las condiciones socioeconómicas. Uno de los estudios analizados señaló la importancia de elaborar estrategias para mitigar los efectos adversos de niveles familiares socioeconómicos desfavorables, que impactaban directamente en el neurodesarrollo de niños prematuros y en sus resultados educativos^(47)^. Asimismo, las condiciones sociales desfavorables, como el bajo ingreso familiar y la pobreza, se asociaron a síntomas de ansiedad y a mayores síntomas de depresión, respectivamente^(11)^, influyendo en el proceso de enfermedad infantil.

Los factores culturales se examinaron predominantemente a partir del idioma y la nacionalidad, evidenciándose una mayor probabilidad de readmisión y un tiempo de permanencia hospitalaria más prolongado entre los hijos de madres inmigrantes en comparación con los de madres nativas^(37)^. Se sabe que las personas que viven como inmigrantes pueden estar expuestas a situaciones de vulnerabilidad e inseguridad, y en ese contexto de cuidado, sus niños pueden presentar un mayor riesgo en salud.

Las cuestiones relacionadas con los factores políticos y las políticas públicas se identificaron a través de beneficios sociales, programas y servicios de protección, entre otros. Dichos programas y beneficios sociales facilitan el acceso a la información y afectan positivamente la salud infantil, como lo demostró el programa *Following Baby Back Home*, que optimiza la búsqueda de cuidados de salud, disminuyendo las tasas de mortalidad de niños nacidos pretérmino con bajo peso al nacer^(38)^. Por otro lado, la supervisión estatal, señalada como indicador de abuso y negligencia predominante entre las madres adolescentes, condujo a un aumento de los riesgos de efectos adversos en lo cognitivo, el lenguaje y lo motor^(30)^.

Aunque los estudios de esta revisión no identificaron aspectos relacionados con el acceso a la alimentación, las investigaciones mostraron que las familias en situación de inseguridad alimentaria requieren, al menos, dos salarios mínimos para cubrir sus gastos mensuales^(51)^. Explorar las condiciones socioambientales a las que están sometidos los niños es un aspecto que debe ser abordado en estudios posteriores, ya que estos determinantes pueden modificar el crecimiento y el desarrollo en la prematuridad^(17)^. La vulnerabilidad relacionada con las condiciones de saneamiento y el acceso a la alimentación aumenta los riesgos de desnutrición, infecciones gastrointestinales y, en consecuencia, la necesidad de acudir a los servicios de salud^(52-53)^.

Siguiendo con la dimensión Condiciones de Vida y de Trabajo, el acceso a los servicios esenciales se investigó desde la perspectiva del seguro de salud, del acceso a intervenciones y a servicios, tales como maternidad y seguimiento ambulatorio. Los servicios especiales, como el programa de visitas domiciliarias o el acceso al servicio social, se señalaron como grandes aliados para la salud de los niños nacidos pretérmino, ya que incrementan los cuidados de salud, reducen el riesgo de mortalidad y mitigan los efectos adversos en el desarrollo^(30,38)^. La limitación en el acceso a los servicios de salud puede estar relacionada con problemas del propio sistema, como la escasez de profesionales y la organización inadecuada, además de cuestiones sociales como la escolaridad y los factores socioeconómicos^(54)^. Así, se infiere que, desde el prenatal, los profesionales deben identificar las barreras que puedan dificultar el acceso de la familia a los servicios, para desarrollar estrategias que mejoren los resultados perinatales.

De este modo, se sabe que los niños egresados de la UCIN, especialmente los pretérmino, tienen un potencial riesgo de alteración en el crecimiento, el desarrollo y una necesidad aumentada de acudir a los servicios de salud por encima de lo habitual debido a las intercurrencias^(12,31)^. Por ello, es necesario que el seguimiento post-alta en servicios ambulatorios esté bien establecido, con el fin de proporcionar una evaluación multidisciplinaria al niño y, en consecuencia, minimizar los posibles daños mediante la identificación precoz de alteraciones y orientaciones de cuidado.

La educación evidenció importantes desenlaces, como el impacto del nivel de escolaridad materna en los niveles de neurodesarrollo de los niños nacidos pretérmino, destacándose el cognitivo y el del lenguaje^(27)^. Otro estudio de la literatura identificó que los niveles económicos y la disponibilidad de recursos en el ambiente domiciliario, como juguetes y actividades realizadas con los padres, mejoraron el desarrollo cognitivo entre los 24 y 42 meses^(55)^. Estos resultados refuerzan la importancia de explorar el entorno en el que el niño está inserto.

En cuanto a cómo viven los niños, se destacan estudios que exploran la habitabilidad^(43)^ y la organización familiar para el cuidado^(44)^. Respecto a las Condiciones de Habitación, los estudios de esta revisión señalaron que un mayor número de habitantes en la residencia aumentó las probabilidades de readmisión^(43)^, lo cual puede estar asociado con la transmisión de enfermedades. Por otro lado, la presencia de más personas en el hogar y tener hermanos se asoció a una menor probabilidad de retraso en la motricidad fina y gruesa, respectivamente^(44)^, ya que los demás miembros de la familia estimulan el desarrollo del niño^(36)^. Sin embargo, en lo que respecta a la condición laboral materna, el hecho de que la niña sea cuidada por una madre que ejerce alguna actividad profesional se relacionó con una mayor probabilidad de que el hijo presente retraso en la motricidad fina^(44)^.

En esta revisión de alcance, la dimensión Redes Sociales y de Apoyo de la Comunidad abarcó 21 artículos e identificó componentes como la situación marital, la composición familiar, el apoyo de familiares y amigos, el cuidador remunerado, entre otros. Se destaca la importancia de la red de apoyo, ya que respalda a la madre y, en consecuencia, proporciona contextos favorables para el desarrollo infantil^(36)^. Además, figuras maternas familiares, como los abuelos, se consideran referentes de conocimiento basados en sus vivencias y pueden influir a partir de sus creencias y valores^(56)^.

La dimensión Factores Conductuales y de Estilo de Vida del Individuo fue la menos explorada por los estudios. Estos resultados pueden haber sido influenciados por el contexto domiciliario de las investigaciones; sin embargo, la literatura señala que el apoyo al cuidado canguro mediante visitas domiciliarias se relacionó con la reducción de la mortalidad neonatal e infantil en bebés con bajo peso al nacer^(57)^. Además, los niños nacidos pretérmino precoces presentan riesgos para el establecimiento favorable de la lactancia materna^(22)^, aumentando las probabilidades de destete precoz, que pueden contrarrestarse mediante orientaciones respecto al inicio y al establecimiento de la amamantación^(57-58)^.

El cuidado familiar interfiere en el desarrollo y el crecimiento adecuado del niño, y puede ampliar o limitar la promoción de la salud de acuerdo con los DSS presentes en la realidad familiar^(36)^. Por lo tanto, considerar la variable cuidado familiar en futuras investigaciones podría permitir una mejor comprensión de los sentimientos vividos y los desafíos enfrentados por los cuidadores, evidenciando la necesidad de elaborar estrategias de apoyo a las familias^(59)^. El cuidado también puede investigarse desde la perspectiva de un cuidador profesional en instituciones educativas, como las guarderías, que, al asociarse al cuidado familiar en el hogar, favorecen el desarrollo infantil.

El apego se refiere a los vínculos establecidos entre los cuidadores y los niños, identificado en esta revisión como la interacción materno-infantil. Algunos estudios revelaron que dicha interacción fue influenciada por la salud emocional materna en casos de estrés, ansiedad y depresión, lo que llevó a ofrecer menos estímulo al niño y, en consecuencia, al aumento de los riesgos para el desarrollo^(10,26)^. Ante estos impactos, se torna indispensable una mayor investigación del apego, permitiendo identificar los factores de riesgo en los cuales los profesionales puedan crear estrategias que orienten y apoyen a las familias, así como los factores de protección que respalden el crecimiento y el desarrollo saludable infantil.

En relación con los hábitos no saludables, en esta revisión quedó evidenciado que los niños que conviven con personas que consumen alcohol de forma abusiva tienen cinco veces más probabilidades de presentar retraso en el neurodesarrollo^(35)^. Considerando que estos hábitos son factores modificables, un estudio apuntó a la necesidad de desarrollar políticas enfocadas en la reducción del consumo de alcohol y tabaco, ya que estos han sido asociados con un desarrollo inadecuado en la primera infancia^(60)^.

Todos los estudios investigaron la dimensión de Características Individuales, empleando las variables edad, sexo y factores hereditarios; condiciones de salud frecuentemente asociadas a la historia obstétrica, la condición al nacer, el crecimiento, el desarrollo y las necesidades de servicios de salud. Los compromisos causados debido a estas variables tienen el potencial de aumentar la demanda de cuidados a lo largo de la vida del niño. Así, la identificación de las variables que se están estudiando permite comprender el impacto de cada una, como es el caso de la asociación entre un mayor tiempo de hospitalización y el deterioro del desarrollo socioemocional, cognitivo y motor^(42,61)^.

Otro punto que se hace necesario discutir frente al contexto de la prematuridad es el abordaje de los factores psicológicos, tratados en 15 artículos. La maternidad se presenta como una fase de vulnerabilidad. Sumado a la prematuridad, un contexto que impone desafíos a la familia y, principalmente, a la madre, se convierte en un factor de riesgo para casos de depresión^(10)^. Además, el soporte limitado de los profesionales a estas familias después del alta resulta en fatiga y aislamiento social, aumentando los costos de salud frente a las complicaciones evitables por un cuidado deficiente en el prematuro^(32)^. Sin embargo, una revisión de literatura reveló que, en ocasiones, el sufrimiento mental materno no es identificado y puede generar repercusiones en la salud integral del niño^(62)^. Frente a ello, se resalta la necesidad de adoptar un cuidado centrado en el paciente y la familia, con el fin de acompañar los factores con potencial influencia, así como establecer estrategias que promuevan el bienestar. Un estudio evidenció que el cuidado centrado en el paciente y la familia genera impactos positivos, tales como un aumento en la satisfacción familiar, mejores resultados y calidad de vida para los niños^(63)^.

Los factores étnicos y raciales exponen una brecha social frente a las desigualdades estructurales, trayendo consecuencias para el proceso de desarrollo y crecimiento infantil en el contexto de condiciones socioeconómicas y raciales. Algunos estudios revisados ejemplificaron esto al demostrar la asociación entre raza, madres adolescentes y lenguaje; por ejemplo, niños negros e hispano-blancos tienen mayor riesgo de retraso en el lenguaje en comparación con los niños blancos^(30,25)^.

Se observa que los desenlaces en salud infantil más relacionados con los DSS y más estudiados han sido el desarrollo, en especial el cognitivo, lingüístico y motor, seguido por las demandas de servicios de salud, relacionadas o no con las afecciones. Estos resultados reflejaron la relevancia del seguimiento del niño en el contexto en que se encuentra inserto, especialmente considerando la criticidad de los primeros dos años de vida debido a la neuroplasticidad, momento oportuno para intervenciones y estímulos al desarrollo^(12)^. De esta forma, se refuerza la necesidad de intervenciones preventivas sobre los factores modificables que representan un riesgo para el crecimiento y el desarrollo pleno, comprometiendo la salud integral infantil.

En este estudio se comprendió que las variables contribuyen a la identificación de las dimensiones de los DSS exploradas en las investigaciones científicas; sin embargo, se reconoce su limitación para representar estas dimensiones en su totalidad, lo cual se consideró en la discusión de los datos analizados. Además, el estudio presenta limitaciones relacionadas con la edad de los niños, ya que excluye aquellos estudios que ampliaron la investigación a mayores de dos años y restringe la comprensión de los DSS a esta franja etaria. Otros contextos de cuidado más allá del domiciliario y la inclusión de literatura gris podrían ofrecer información importante sobre la salud infantil.

## Conclusión

La identificación sobre la salud del niño prematuro a través del referente teórico de los DSS evidenció los aspectos protectores y amenazantes del ambiente en el que viven los niños. Los estudios analizados mostraron un mayor interés en explorar las variables relacionadas con las características del individuo, destacándose las condiciones de salud, así como la edad, el sexo y los factores hereditarios, resaltando la búsqueda de factores de impacto directo en la salud infantil. Por otro lado, pocos estudios investigaron las variables de las dimensiones *Condiciones Económicas, Sociales, Culturales y Ambientales; Redes Sociales y de Apoyo de la Comunidad; y Factores Conductuales y de Estilo de Vida del Individuo*, lo que indica las lagunas existentes en la comprensión de los DSS en la salud infantil.

Cabe destacar que los estudios investigaron las variables de forma aislada dentro de las dimensiones de los DSS, lo que dificulta la identificación de inequidades y el análisis de su repercusión en la salud de los prematuros. Es fundamental que los futuros estudios aborden los DSS de forma integrada, permitiendo una comprensión de las condiciones de vida de la población estudiada y de los impactos del modo de vivir y enfermar de los niños nacidos pretérmino en los primeros dos años de vida sobre el crecimiento, el desarrollo y el comportamiento infantil. Este enfoque ampliado facilitará la elaboración de cuidados centrados en el individuo y permitirá intervenciones precoces frente a los desenlaces en salud infantil.
